# Silver‐Catalyzed Stereoselective Aminosulfonylation of Alkynes

**DOI:** 10.1002/anie.201705122

**Published:** 2017-07-07

**Authors:** Yongquan Ning, Qinghe Ji, Peiqiu Liao, Edward A. Anderson, Xihe Bi

**Affiliations:** ^1^ Jilin Province Key Laboratory of Organic Functional Molecular Design & Synthesis Department of Chemistry Northeast Normal University Changchun 130024 China; ^2^ State Key Laboratory of Elemento-Organic Chemistry Nankai University Tianjin 300071 China; ^3^ Chemistry Research Laboratory University of Oxford 12 Mansfield Road Oxford OX1 3TA UK

**Keywords:** alkynes, aminosulfonylation, radical reactions, silver catalysis, stereoselectivity

## Abstract

A silver‐catalyzed intermolecular aminosulfonylation of terminal alkynes with sodium sulfinates and TMSN_3_ is reported. This three‐component reaction proceeds through sequential hydroazidation of the terminal alkyne and addition of a sulfonyl radical to the resultant vinyl azide. The method enables the stereoselective synthesis of a wide range of β‐sulfonyl enamines without electron‐withdrawing groups on the nitrogen atom. These enamines are found to be suitable for a variety of further transformations.

Alkynes are one of the most common and versatile functional groups in organic synthesis, and catalytic methods that enable their efficient transformation into other useful functionalities are therefore highly appealing in both academic research and industrial applications.^1^ Direct difunctionalization reactions of alkynes, capable of affording tri‐ and tetrasubstituted alkenes, have attracted much attention in recent years.^2^ Among these, radical‐based 1,2‐difunctionalizations of alkynes offer a straightforward means to construct functionalized alkenes by reaction with both carbon‐3 and heteroatom‐centered radicals^4^ with excellent step‐ and atom‐economy.^5^ Mechanistically, a common reaction pathway is observed involving initiation of the reaction by radical addition to the alkyne to generate a vinyl radical intermediate, which is then coupled with another component to form the alkene product (Figure 1 a). However, such vinyl radical species are highly reactive and readily undergo hydrofunctionalization by H‐atom abstraction,^6^ which is a significant challenge in developing radical‐based alkyne difunctionalization reactions. Moreover, aliphatic alkynes are generally unreactive in these processes, which is most likely due to the lack of a π‐conjugation stabilizing effects of intermediate alkyl‐substituted vinyl radicals compared to aryl‐substituted analogues.^7^ Consequently, conceptually distinct approaches are in high demand. In the last years, the nitrogenation of alkynes with trimethylsilylazide (TMSN_3_) has attracted much attention, where carbon–carbon triple bond cleavage leads to a variety of nitrogen‐containing molecules.^8^ Building from our recent efforts on the activation of alkynes by silver catalysis,^9^ we herein report a new strategy to effect radical‐based difunctionalization of terminal alkynes through an unprecedented hydroazidation/ radical addition cascade (Figure 1 b). The key point for this successful transformation is that we discovered a mild and efficient approach to generate sulfonyl radical from sodium sulfinate, thus avoiding the initial competitive radical addition to alkynes.^6^ To the best of our knowledge, this is the first example of intermolecular alkyne aminosulfonylation,^10^ resulting in stereoselective synthesis of β‐sulfonyl *N*‐unprotected enamines, which are useful synthetic intermediates whose applications are currently limited by a lack of practical synthetic methods for their preparation.^11^ Moreover, only one report regarding the synthesis of *N*‐unprotected enamines by alkyne difunctionalization has been reported.^12^

**Figure 1:**
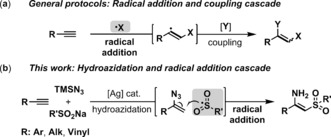
Strategies for radical difunctionalization of alkynes.

Initial optimization of the reaction was performed using alkyne **1 a**, TMSN_3_, and sodium *p*‐toluenesulfinate **2 a**, with variation of reaction parameters including metal catalyst, solvent, and temperature (Table 1). Silver salts proved highly effective in promoting the hydroazidation/ sulfonation cascade; Ag_2_CO_3_, Ag_3_PO_4_, and AgF all gave the aminosulfonylated product **3 a** in high yields, with Ag_3_PO_4_ delivering an optimum yield of 85 % (entries 1–3). In contrast, other metal catalysts such as Pd(OAc)_2_, CuI, and Mn(OAc)_3_ were ineffective, which is presumably due to their inability to catalyze the hydroazidation reaction (entries 4–6). Water was found to be an essential additive, as a poor yield (30 %) was obtained in its absence (entry 7); the use of less than two equivalents of TMSN_3_ also resulted in a decrease in product yield. The reaction solvent also proved important, with DCE and 1,4‐dioxane giving only trace amounts of the desired product using Ag_3_PO_4_ as catalyst (entries 8 and 9), compared to polar aprotic solvents such as NMP (65 %, entry 10) and DMSO. Finally, increasing or reducing the reaction temperature led to a decrease in product yield (entries 11 and 12).

**Table 1:** Optimization of the reaction conditions.^[a]^ 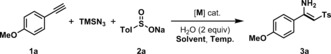

Entry	[M] cat.	Amount [mol %]	Solvent	*T* [°C]	Yield [%]^[b]^
1	Ag_2_CO_3_	20	DMSO	70	75
2	Ag_3_PO_4_	20	DMSO	70	85
3	AgF	20	DMSO	70	63
4	Pd(OAc)_2_	5	DMSO	70	0^[c]^
5	CuI	20	DMSO	70	0^[c]^
6	Mn(OAc)_3_	20	DMSO	70	0^[c]^
7	Ag_3_PO_4_	20	DMSO	70	30^[d]^
8	Ag_3_PO_4_	20	DCE	70	trace
9	Ag_3_PO_4_	20	1,4‐dioxane	70	trace
10	Ag_3_PO_4_	20	NMP	70	65
11	Ag_3_PO_4_	20	DMSO	100	62
12	Ag_3_PO_4_	20	DMSO	50	55

[a] Standard reaction conditions: **1 a** (0.5 mmol), TMSN_3_ (1.0 mmol), **2 a** (1.0 mmol), H_2_O (1.0 mmol), in DMSO (2 mL) at 70 °C under open‐to‐air conditions for 4 h. [b] Yield of isolated product. [c] No **3 a** was detected by ^1^H NMR spectroscopic analysis of crude reaction mixture. [d] Without H_2_O. DCE=1,2‐dichloroethane, DMSO=dimethylsulfoxide, NMP=*N*‐methyl pyrrolidone.

The scope of the reaction with respect to the alkyne proved broad, with a wide range of aryl‐ and heteroaryl‐functionalized terminal alkynes being suitable for this silver‐catalyzed cascade reaction, affording the corresponding β‐sulfonyl enamines in good to excellent yields (Scheme 1). For instance, a variety of *para*‐substituted phenylacetylenes **1 a**–**1 j** underwent smooth reaction with TMSN_3_ and **2 a** to give the products **3 a**–**3 j** in 69–87 % yields. Pleasingly, common functional groups such as alkoxy, alkyl, aryl, halogen, cyano, trifluoromethyl, aldehyde, and ester were all well‐tolerated, with X‐ray diffraction analysis of **3 c** confirming the (*Z*)‐configuration of the alkene. Similarly, *ortho*‐, *meta*‐, and 3,4‐disubstituted phenylacetylenes gave the desired enamine products **3 k**–**3 p** in high yields (79–83 %). Heteroaryl acetylenes including 2‐ and 3‐pyridyl, 2‐ and 3‐thienyl, as well as ferrocenyl acetylene were also evaluated, and the corresponding products **3 s**–**3 w** were obtained with high efficiency. A more elaborate estrone‐derived terminal alkyne could also be successfully transformed into the corresponding β‐sulfonyl enamine **3 x** (79 %), underlining the robust nature of the method.

**Scheme 1:**
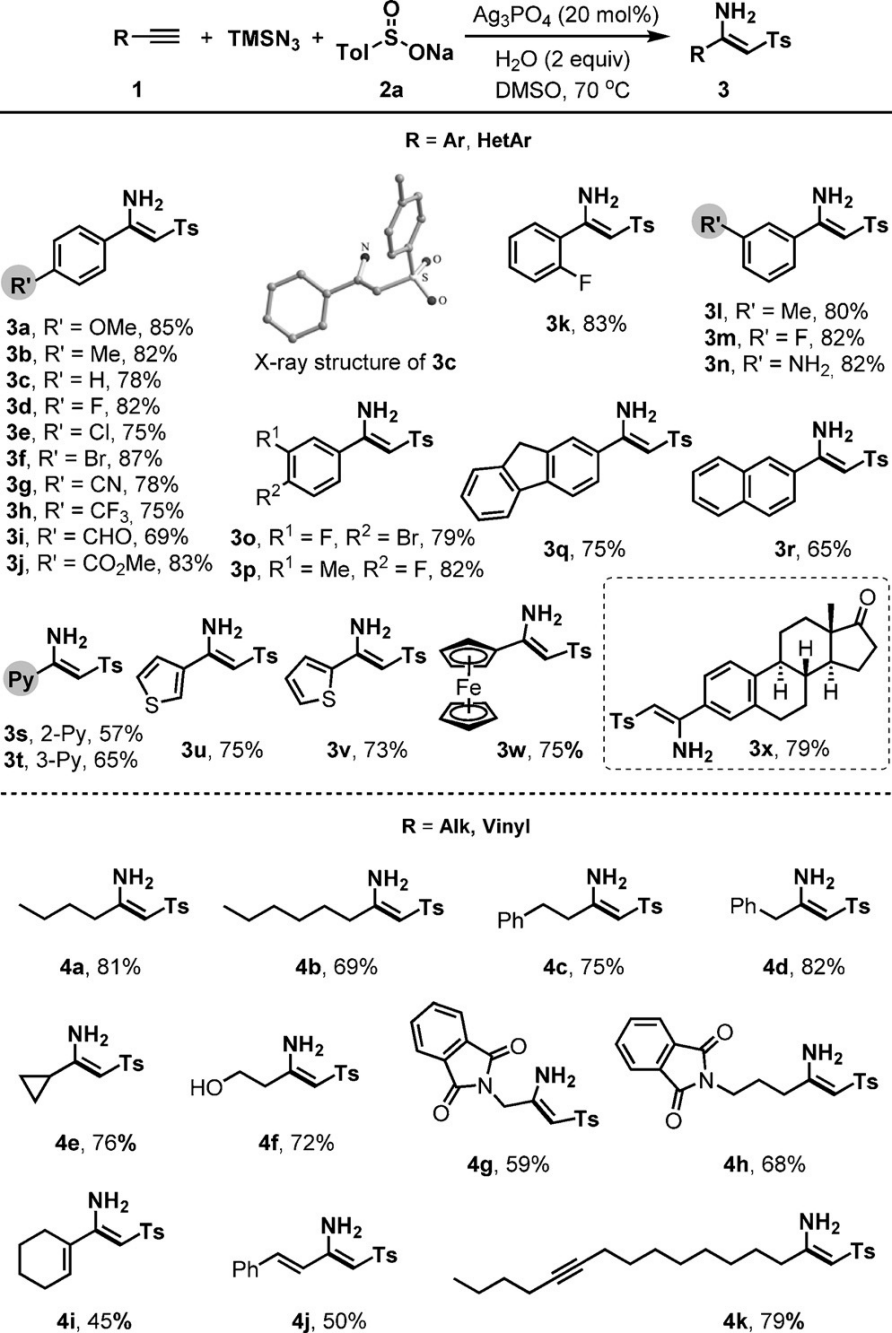
Reaction scope: aromatic and aliphatic alkynes.

Following this success with aryl‐substituted alkynes, we turned our attention to the reactivity of aliphatic alkynes, where it is notable that alkyl‐substituted β‐sulfonyl enamines have not been prepared by other methods. In the event, we were delighted to find that reactions of alkyl‐substituted terminal alkynes generally proceeded with equal efficiency to aryl alkynes, affording a number of functionalized β‐sulfonyl enamines **4** in high yields. Apart from simple alkyl acetylenes (**4 a**–**4 d**, 69–81 %), cyclopropyl, hydroxyl, and phthalimide substituents were all tolerated, giving the functionalized sulfonyl enamines **4 e**–**4 h** (59–82 %). Further, enyne systems such as 1‐cyclohexenyl and styryl acetylenes also participated efficiently in this three‐component reaction, giving the 1,3‐butadienes **4 i** and **4 j** in reasonable yields. The presence of an internal alkyne did not affect the formation of β‐sulfonyl enamine **4 k** (79 %), illustrating the exquisite chemoselectivity between internal and terminal alkynes. All of these heteroaryl‐ and alkyl‐substituted β‐sulfonyl enamines in Scheme 1 are novel compounds that could prove useful as intermediates in organic and medicinal chemistry research.

We next set out to evaluate the reaction scope with respect to the sulfinate component in reactions with 4‐phenyl phenylacetylene **1 y** (Table 2). Whether electron‐rich or electron‐deficient, aryl sulfinates afforded the corresponding β‐sulfonyl enamines in high yields (**5 a**–**5 c**, 75–82 %). Alkyl sulfinate salts also proved suitable reaction partners, giving products **5 d**–**5 f** with similar efficiency.

**Table 2:** Reaction scope: sodium sulfinates. 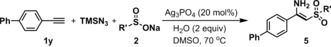

Entry	R′	Product **5**	Isolated yield [%]
1	4‐MeC_6_H_4_	**5 a**	75
2	C_6_H_5_	**5 b**	82
3	4‐ClC_6_H_4_	**5 c**	75
4	Me	**5 d**	75
5	Et	**5 e**	85
6	cyclopropyl	**5 f**	87

To gain insight into the reaction mechanism, the reaction of 4‐ethynyltoluene **1 b** was monitored by ^1^H NMR spectroscopy under standard reaction conditions in [D_6_]DMSO (Figure 2 a). By comparison with authentic samples, signal **A** at 4.1 ppm was assigned as the acetylenic proton of **1 b**. After 40 min, this signal had almost completely disappeared, and new doublets at 4.97 and 5.61 ppm were assigned as the olefinic hydrogens of vinyl azide **VA**, which reached a maximum intensity after 20 min. The singlet at 5.07 ppm was assigned as the olefinic hydrogen of product **3 b**, which appeared after about 20 min, and was almost the sole reaction component by 180 min. This result clearly suggests initial rapid alkyne hydroazidation, followed by slower addition of sulfonyl radical to the vinyl azide generated in situ, and also illustrates the clean conversion of the terminal alkyne to the β‐sulfonyl enamine. Further information on the radical addition step was obtained by submission of vinyl azide (**VA**) to various reaction conditions (Figure 2 b), which revealed a dual role of TMSN_3_: in the absence of TMSN_3_, no reaction took place, whereas in the presence of 1.5 equivalents, product **3 b** was afforded in high yield (85 %) after a short reaction time (40 min). This implies that TMSN_3_ plays a critical role in generation of the sulfonyl radical. That this second step indeed involves a radical intermediate was supported by the formation of only a trace amount of **3 b** in the presence of TEMPO.

**Figure 2:**
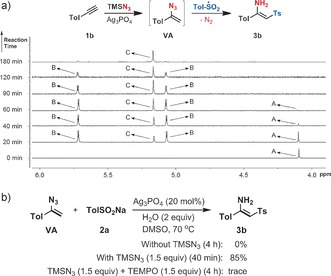
Mechanistic investigations. a) Reaction monitoring by ^1^H NMR spectroscopy. The reaction process of **1 b** was monitored by ^1^H NMR spectroscopy (600 MHz, [D_6_]DMSO). **A**: acetylenic hydrogen of **1 b**; **B**: two olefinic hydrogens of vinyl azide (**VA**); **C**: olefinic hydrogen of product **3 b**. b) The crucial role of TMSN_3_ in the sulfonyl radical addition to vinyl azides.

On the basis of above experimental results, a possible mechanism is illustrated in Scheme 2. Initially, AgN_3_ is generated by anion exchange of TMSN_3_ with Ag_3_PO_4_.^13^ Its subsequent addition to the terminal alkyne **1 a** produces vinylsilver intermediate **A′**.8a Meanwhile, TolSO_2_TMS (**A**) is generated from sulfinate salt **2 a** (possibly promoted by Ag^I^). Such intermediates are known to be somewhat unstable,^14^ and could be oxidized by Ag^I^ to give a radical cation **B**,^15^ which could then release sulfonyl radical **C** and a trimethylsilyl cation.9g The latter is captured by water to produce trimethylsilanol with release of a proton, which could affect protodemetalation of intermediate **A′** to give the observed vinyl azide (**VA**). This in turn is readily attacked by the sulfonyl free radical **C**, leading to carbon‐centered radical **D**,^16^ which rapidly converts to iminyl radical **E** with release of N_2_. Following sequential reduction and protonation, an imine intermediate (**G**) is formed. Product **3 b** is obtained by tautomerization of this imine.^17^ Stereochemistry of the product should be ascribed to the intramolecular hydrogen‐bonding effect. Note that the screening of a variety of potential radical precursors, such as Togni's reagent, diphenylphosphine oxide, and potassium 2‐oxo‐2‐phenylacetate,^18^ were not successful in the desired aminofunctionalization of terminal alkynes, and therefore demonstrated the generation of sulfonyl radical under above mild conditions appears to play a crucial role in controlling the reaction sequence.9b, 19

**Scheme 2:**
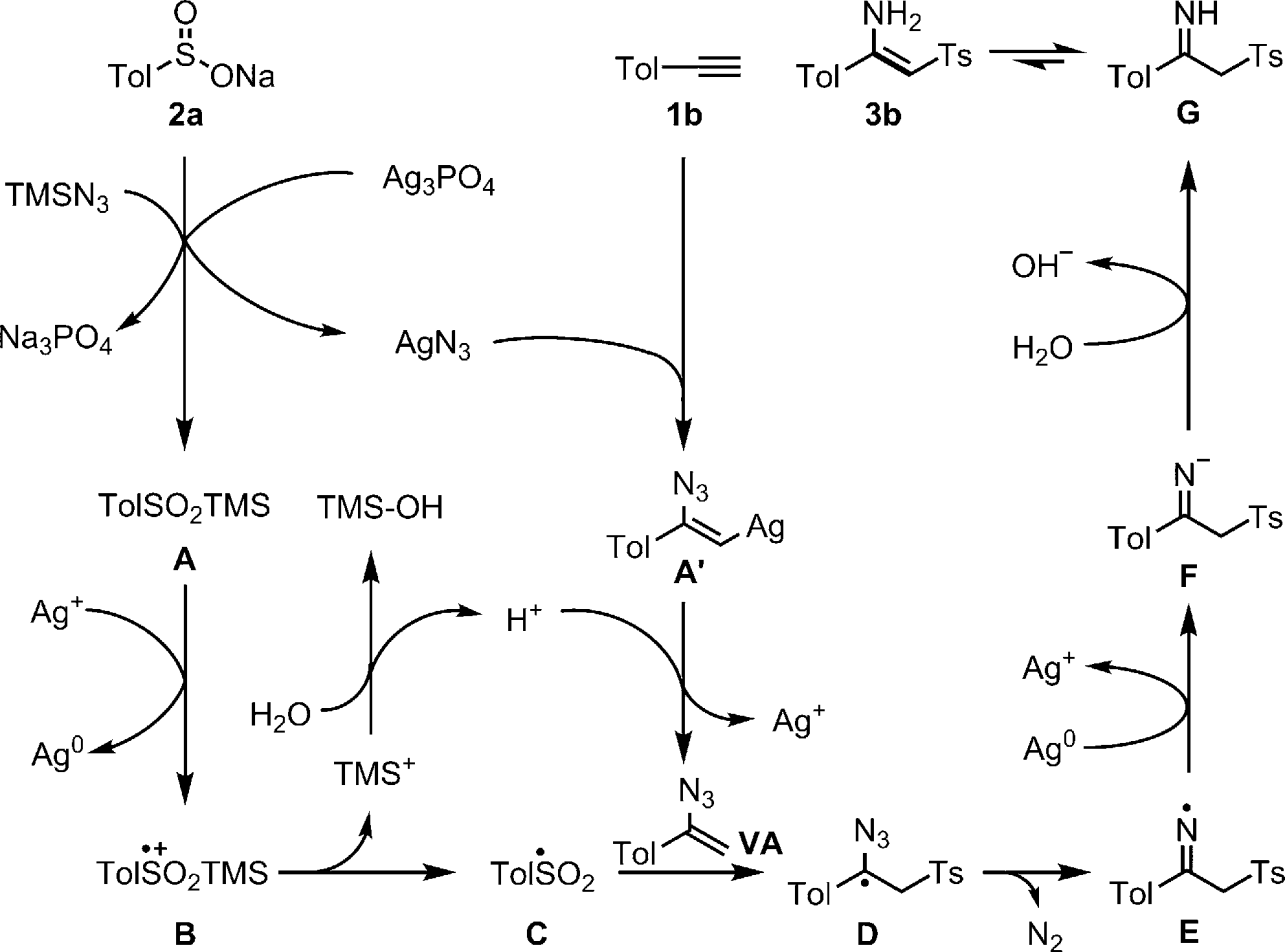
Proposed reaction mechanism.

Finally, a gram scale reaction of phenylacetylene **1 c**, TMSN_3_ and sodium *p*‐toluenesulfinate **2 a** was tested; delightfully, this reaction could be performed on 20 mmol scale and proceeded smoothly to give product **3 c** with only a modest decrease in yield (3.11 g, 57 %; Scheme 3). Next, further synthetic manipulations were conducted to explore its reactivity. As reported by Jiang,^11^ β‐ketosulfone **6** could be prepared by treatment of **3 c** with silica gel (95 %), while 2*H*‐azirine **7** was obtained in 65 % yield through hypervalent iodine‐induced oxidative cyclization.^20^ Unexpectedly, dihydropyrrole **8** was obtained under Bao and Guan's K_2_S_2_O_8_‐mediated oxidative cyclization conditions, instead of a pyrrole as observed with analogous β‐keto or β‐ester enamines.^21^ Similarly, a new reaction pattern was discovered on treatment of **3 c** with NBS, which afforded polybrominated imine **9** in 72 % yield. *N*‐brominated imines have rarely been reported.^22^

**Scheme 3:**
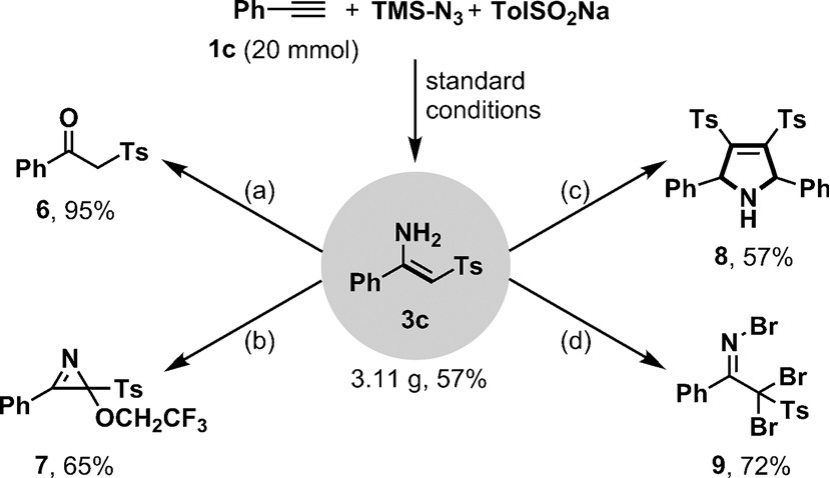
Gram‐scale synthesis and further transformations. Reaction conditions: a) Stir in CH_2_Cl_2_ with silica gel at room temperature, overnight; b) PhIO (2.0 mmol) in TFE (5 mL) stirred at rt for 15 min, then substrate **3 c** (1 mmol in 5 mL of TFE) was added dropwise; c) **3 c** (0.5 mmol), K_2_S_2_O_8_ (0.6 mmol), DMSO (5 mL), 5 h; d) **3 c** (0.5 mmol), NBS (1.65 mmol ), DCE (3 mL), at rt for 12 h. Yields of isolated product [%] are given.

In summary, a convenient and functional‐group‐tolerant silver‐catalyzed three‐component reaction of terminal alkynes, TMSN_3_, and sodium sulfinates has been developed, which shows broad substrate scope with respect to both the alkyne and sulfinate. The reaction proceeds through an unprecedented sequence of alkyne hydroazidation, and radical addition of a sulfonyl radical to the in situ generated vinyl azide. This strategy represents an appealing means to achieve alkyne aminofunctionalization under mild reaction conditions; extension to other radical species is under way, and will be reported in due course.

## Conflict of interest

The authors declare no conflict of interest.

## Supporting information

As a service to our authors and readers, this journal provides supporting information supplied by the authors. Such materials are peer reviewed and may be re‐organized for online delivery, but are not copy‐edited or typeset. Technical support issues arising from supporting information (other than missing files) should be addressed to the authors.

Supplementary
