# Supplementary material for: Silver‐Catalyzed Stereoselective Aminosulfonylation of Alkynes
Source: Angew Chem Int Ed Engl. 2017 Jul 7;56(44):13805–8. doi: 10.1002/anie.201705122 (PMC5655761; doi:10.1002/anie.201705122)
Supplement: Supplementary file 1 — Supplementary [file ANIE-56-13805-s001.pdf]

Angewandte Chemie
Eine Zeitschrift der Gesellschaft Deutscher Chemiker

Supporting Information

Silver-Catalyzed Stereoselective Aminosulfonylation of Alkynes

*Yongquan Ning, Qinghe Ji, Peiqiu Liao, Edward A. Anderson, and Xihe Bi**

anie_201705122_sm_miscellaneous_information.pdf

Supporting Information

Contents

I. General information S3

II. Screening of radical species S3

III. Synthesis and analytical data of **3a-3x**, **4a-4f**, **5-9**... .... S3

IV. Crystallography of **3c** S17

V. NMR spectra copies S19

I. General information

All reagents were purchased from commercial sources and used without treatment, unless otherwise indicated. The products were purified by column chromatography over silica gel. **^{1}H-NMR** and **^{13}C-NMR** spectra were recorded at 25 ºC on a Varian 500 MHz and 125 MHz, respectively, and TMS was used as internal standard. Mass spectra were recorded on BRUKER AutoflexIII Smartbeam MS-spectrometer. High resolution mass spectra (HRMS) were recorded on Bruck microTof by using ESI method.

II. Screening of Radical Species[1, 2, 3.]

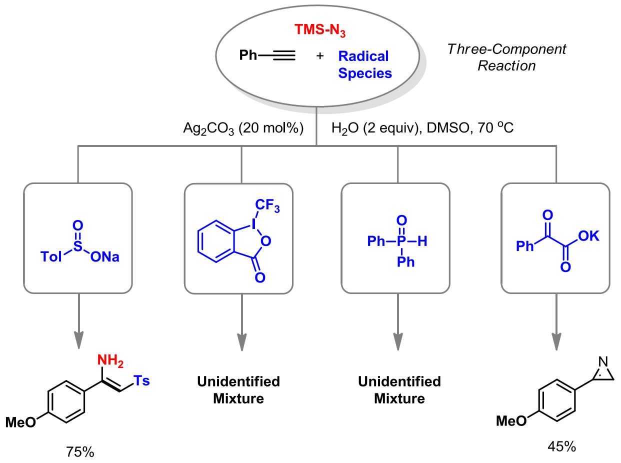

III. Synthesis and analytical data of 3a-3x, 4a-4f, 5-9.

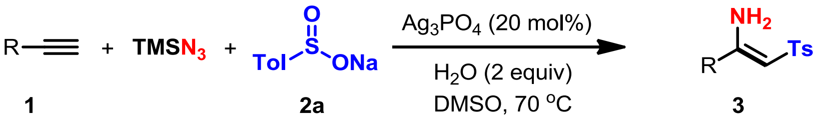

Typical synthetic procedure (with 3a as an example): To a solution of 1-ethynyl-4-methoxybenzene (**1a**) (66 mg, 0.5 mmol) and TMS-N_3 (0.1 mL, 0.75 mmol) in DMSO (2 mL) at 70 ^{o} C, TsNa (178mg, 1.0 mmol), Ag_3PO_4(41.8 mg, 0.10 mmol) was added. The mixture was then stirred for 3.0 h until substrate **1a** disappeared. The resulting mixture was concentrated and taken up by dichloromethane (3 x 15 mL). The organic layer was washed with brine (3 x 40 mL), dried over $MgSO_4$ and concentrated. Purification of the crude product with flash column chromatography (silica gel; petroleum ether: ethyl acetate = 10:1) gave **3a** in 85% yield as a white solid.

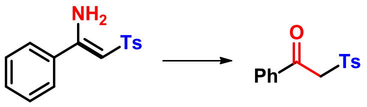

3c **6**, 95%

General Procedure for the Synthesis of *β*-ketosulfones 6[1] **:** Accoding to Jiang's work, **3c** (0.5 mol) , in CH_2Cl_2 with silica gel at room temperature overnight. The resulting mixture was filtered and concentrated, and then the crude product was purified by column chromatography on silica gel using petroleum ether/EtOAc as eluent to provide the pure target product **6** in 95% as a white solid.

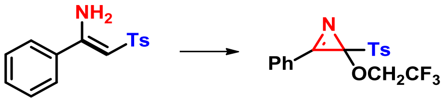

3c **7**, 65%

General Procedure for the Synthesis of 2H-Azirines 7[2] **:** To a suspension of iodosobenze (PhIO, 2.1 mmol) in TFE (5 mL), stirred at room temperature for about

15 min, was added dropwise a solution of enamine **3c** (0.5 mmol) in TFE (5 mL). The reaction mixture was kept at the same temperature for about 15-30 min until TLC indicated the total consumption of enamine **3c**. The reaction mixture was then quenched with saturated aqueous $NaHCO_3$ (10 mL) and extracted with DCM (10 mL x 3). The organic layers were combined and dried over anhydrous Na_2SO_4. The solvent was evaporated, and the residue was passed through a silica gel column, using a mixture of PE and EtOAc as eluent, to give the desired products 7 in 65% as a yellow oil..

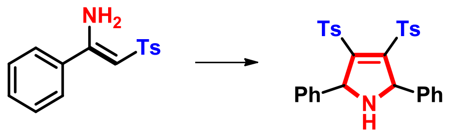

3c **8**, 57%

General Procedure for the Synthesis of dihydropyrrole 8[3] **:** A sealed tube was charged with enamine **3c** (0.5 mmol), DMSO (5 mL), and then $K_2S_2O_8$ (0.6 mmol) was added. The reaction mixture was stirred at 100 °C for 5 h. Then the mixture was cooled to room temperature, and 20 mL water was added to the mixture, which was extracted with ethyl acetate (3 x 10 mL). The organic layer was combined and dried with anhydrous Na_2SO_4. After removal of the solvent under reduced pressure, the residue was separated by flash column chromatography to afford the pure product **8** in 57% as yellow solid.

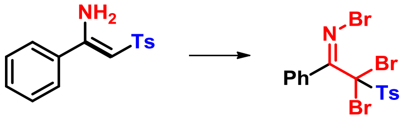

3c **9**, 72%

General Procedure for the Synthesis of poly-brominated imine 9[4] **:** A sealed tube was charged with enamine **3c** (0.5 mmol), DCM (5 mL), and then NBS (3.3 mmol) was added. The reaction mixture was stirred at room temperature overnight.Then 20 mL water was added to the mixture, which was extracted with ethyl acetate (3 × 10 mL). The organic layer was combined and dried with anhydrous Na_2SO_4. After removal of the solvent under reduced pressure, the residue was separated by flash column chromatography to afford the pure product **9** in 72% as white solid.

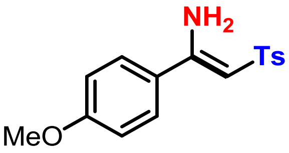

(3a) White Solid; mp: 137-138 °C; **^{1}H NMR** (600 MHz, $CDCl_3$) δ 7.83 (d, J = 8.4 Hz, 2H), 7.41 (d, J = 8.4 Hz, 2H), 7.29 (d, J = 8.4 Hz, 2H), 6.88 (d, J = 8.4 Hz, 2H), 5.90 (s, br, 2H), 5.06 (s, 1H), 3.82 (s, 3H), 2.41 (s, 3H). **^{13}C NMR** (150 MHz, $CDCl_3$) δ 161.67, 155.75, 142.91, 141.87, 129.55, 129.12, 127.76, 126.01, 114.26, 91.44, 55.43, 21.51. **HRMS** (ESI) m/z calcd. For $C_{16}H_{17}NO_3SNa$ $[M+Na]^+$ 326.0827, found 326.0830.

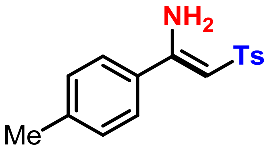

(3b) White Solid; mp: 125-127 °C; **^{1}H NMR** (600 MHz, $CDCl_3$) δ 7.83 (d, *J* = 7.8 Hz, 2H), 7.35 (d, *J* = 7.8 Hz, 2H), 7.29 (d, *J* = 7.8 Hz, 2H), 7.18 (d, *J* = 7.8 Hz, 2H), 5.92 (s, br, 2H), 5.08 (s, 1H), 2.41 (s, 3H), 2.36 (s, 3H). **^{13}C NMR** (150 MHz, $CDCl_3$) δ 156.03, 142.90, 141.73, 141.09, 134.04, 129.55, 129.50, 126.13, 125.97, 91.76, 21.46, 21.23. **HRMS** (ESI) m/z calcd. For $C_{16}H_{17}NO_2SNa$ $[M+Na]^+$ 310.0878, found 310.0880.

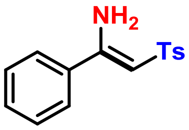

(3c) Yellow Solid; mp: 111-112 °C; **^{1}H NMR** (500 MHz, $CDCl_3$) δ 7.82 (d, *J* = 8.0 Hz, 2H), 7.47-7.43 (m, 2H), 7.43-7.39 (m, 1H), 7.36 (t, *J* = 8.0 Hz, 2H), 7.28 (d, *J* = 8.0 Hz, 2H), 5.92 (s, br, 2H), 5.07 (s, 1H), 2.40 (s, 3H). **^{13}C NMR** (150 MHz, $CDCl_3$) δ 156.12, 143.05, 141.67, 137.00, 130.75, 129.59, 128.94, 126.33, 126.04, 92.27, 21.51. **HRMS** (ESI) m/z calcd. For $C_{15}H_{15}NO_2SNa$ $[M+Na]^+$ 296.0721, found 296.0725.

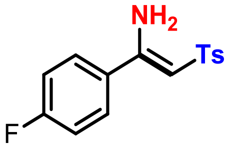

(3d) White Solid; mp: 122-123 °C; **^{1}H NMR** (500 MHz, $CDCl_3$) δ 7.81 (d, *J* = 8.0 Hz, 2H), 7.49-7.42 (m, 2H), 7.29 (d, *J* = 8.0 Hz, 2H), 7.08-7.02 (m, 2H), 5.90 (s, br, 2H), 5.03 (s, 1H), 2.41 (s, 3H). **^{13}C NMR** (150 MHz, $CDCl_3$) δ 164.12 (d, *J* = 250.5 Hz), 154.97, 143.18, 141.50, 133.12 (d, *J* = 3.0 Hz), 129.62, 128.42 (d, *J* = 7.5 Hz), 126.07, 116.03 (d, *J* = 22.5 Hz), 92.68, 21.52. **HRMS** (ESI) m/z calcd. For $C_{15}H_{14}FNO_2SNa$ $[M+Na]^+$ 314.0627, found 314.0632.

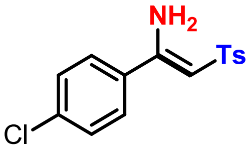

(3e) White Solid; mp: 118-119 °C; **^{1}H NMR** (600 MHz, $CDCl_3$) δ 7.84 (d, J = 8.4 Hz, 2H), 7.42 (d, J = 8.4 Hz, 2H), 7.37 (d, J = 8.4 Hz, 2H), 7.32 (d, J = 8.4 Hz, 2H), 5.96 (s, br, 2H), 5.08 (s, 1H), 2.44 (s, 3H). **^{13}C NMR** (150 MHz, $CDCl_3$) δ 154.82, 143.25, 141.39, 136.79, 135.41, 129.65, 129.18, 127.73, 126.08, 92.86, 21.53. **HRMS** (ESI) m/z calcd. For $C_{15}H_{14}ClNO_2SNa$ $[M+Na]^+$ 330.0331, found 330.0335.

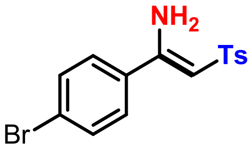

(3f) White Solid; mp: 121-122 °C; **^{1}H NMR** (600 MHz, $CDCl_3$) δ 7.82 (d, J = 8.4 Hz, 2H), 7.51 (d, J = 8.4 Hz, 2H), 7.33 (d, J = 8.4 Hz, 2H), 7.30 (d, J = 8.4 Hz, 2H), 5.91 (s, br, 2H), 5.06 (s, 1H), 2.42 (s, 3H). **^{13}C NMR** (150 MHz, $CDCl_3$) δ 154.78, 143.28, 141.34, 135.94, 132.19, 129.66, 127.92, 126.12, 125.07, 93.08, 21.54. **HRMS** (ESI) m/z calcd. For $C_{15}H_{14}BrNO_2SNa$ $[M+Na]^+$ 373.9826, found 373.3830.

---.

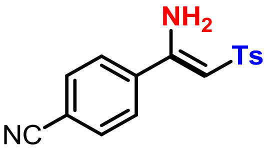

(3g) White Solid; mp: 137-138 °C; **^{1}H NMR** (500 MHz, $CDCl_3$) δ 7.84-7.79 (m, 2H), 7.71-7.62 (m, 2H), 7.57 (d, J = 7.5Hz, 2H), 7.31(d, J = 7.5Hz, 2H), 5.90 (s, br, 2H), 5.09 (s, 1H), 2.42 (s, 3H). **^{13}C NMR** (150 MHz, $CDCl_3$) δ 153.71, 143.62, 141.30, 140.91, 132.73, 129.75, 127.25, 126.20, 117.86, 114.40, 94.63, 21.56. **HRMS** (ESI) m/z calcd. For $C_{16}H_{14}N_2O_2SNa$ $[M+Na]^+$ 321.0674, found 321.0680.

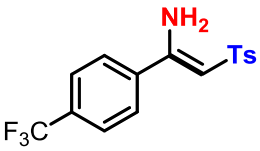

(3h) White Solid; mp: 108-109 °C; **^{1}H NMR** (600 MHz, $CDCl_3$) δ 7.83 (d, J = 7.8 Hz, 2H), 7.64 (d, J = 8.4 Hz, 2H), 7.58 (d, J = 7.8 Hz, 2H), 7.31 (d, J = 8.4 Hz, 2H), 5.91 (s, br, 2H), 5.10 (s, 1H), 2.42 (s, 3H). **^{13}C NMR** (150 MHz, $CDCl_3$) δ 154.32, 143.45, 141.13, 140.54, 132.61 (q, J = 33.0 Hz), 129.70, 126.91, 126.16, 126.01 (q, J = 3.5 Hz), 123.61 (q, J = 271.5 Hz), 94.07, 21.53. **HRMS** (ESI) m/z calcd. For $C_{16}H_{14}F_3NO_2SNa$ $[M+Na]^+$ 364.0595, found 364.0599.

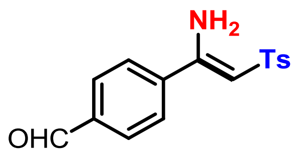

(3i) White Solid; mp: 115-116 °C; ^{1}**H NMR** (600 MHz, DMSO) δ 10.05 (s, 1H), 7.94 (d, *J* = 7.8 Hz, 2H), 7.83 (d, *J* = 7.8 Hz, 2H), 7.74 (d, *J* = 7.8 Hz, 2H), 7.40 (d, *J* = 7.8 Hz, 2H), 7.14 (s, 2H), 5.23 (s, 1H), 2.39 (s, 3H). ^{13}**C NMR** (150 MHz, DMSO) δ 193.15, 155.61, 143.15, 142.29, 141.88, 137.86, 130.07, 130.03, 128.13, 126.14, 91.40, 21.45. **HRMS** (ESI) m/z calcd. For $C_{16}H_{15}NO_3SNa$ $[M+Na]^+$ 324.0670, found 324.0673.

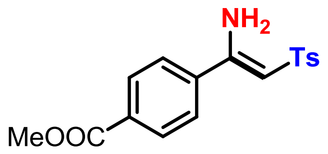

(3j) White Solid; mp: 124-125 °C; ^{1}**H NMR** (600 MHz, $CDCl_3$) δ 8.03 (d, *J* = 8.4 Hz, 2H), 7.83 (d, *J* = 7.8 Hz, 2H), 7.53 (d, *J* = 8.4 Hz, 2H), 7.30 (d, *J* = 7.8 Hz, 2H), 5.92 (s, br, 2H), 5.12 (s, 1H), 3.92 (s, 3H), 2.42 (s, 3H). ^{13}**C NMR** (150 MHz, $CDCl_3$) δ 166.14, 154.76, 143.36, 141.26, 141.18, 132.15, 130.16, 129.68, 126.46, 126.15, 93.69, 52.38, 21.54. **HRMS** (ESI) m/z calcd. For $C_{17}H_{17}NO_4SNa$ $[M+Na]^+$ 354.0776, found 354.0779.

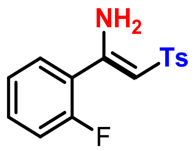

(3k) White Solid; mp: 108-109 °C; ^{1}**H NMR** (600 MHz, $CDCl_3$) δ 7.84 (d, *J* = 7.8 Hz, 2H), 7.43-7.35 (m, 2H), 7.30 (d, *J* = 8.4 Hz, 2H), 7.15 (t, *J* = 7.8Hz, 1H), 7.05-7.00 (m, 1H), 5.93 (s, br, 2H), 4.96 (s, 1H), 2.35 (s, 3H). ^{13}**C NMR** (150 MHz, $CDCl_3$) δ 159.37 (d, *J* = 250.5 Hz), 151.21, 143.16, 141.40, 131.90 (d, *J* = 9.0 Hz), 129.60, 129.35 (d, *J* = 3.0 Hz), 126.05, 124.62 (d, *J* = 4.5 Hz), 124.48 (d, *J* = 12.0 Hz), 116.53 (d, *J* = 22.5 Hz), 94.28, 21.50. **HRMS** (ESI) m/z calcd. For $C_{15}H_{14}FNO_2SNa$ $[M+Na]^+$ 314.0627, found 314.0630.

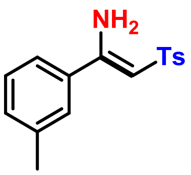

(3l) White Solid; mp: 138-139 °C; **^{1}H NMR** (600 MHz, $CDCl_3$) δ 7.75 (d, *J* =7.8 Hz, 2H), 7.21 (d, *J* = 7.8 Hz, 2H), 7.20-7.14 (m, 4H), 5.82 (s, br, 2H), 5.00 (s, 1H), 2.34 (s, 3H), 2.27 (s, 3H). **^{13}C NMR** (150 MHz, $CDCl_3$) δ 156.20, 142.94, 141.67, 138.76, 136.95, 131.43, 129.50, 128.78, 126.87, 125.98, 123.33, 92.09, 21.46, 21.27. **HRMS** (ESI) m/z calcd. For $C_{16}H_{17}NO_2SNa$ $[M+Na]^+$ 310.0878, found 310.0880.

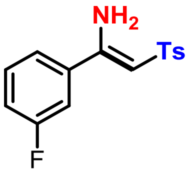

(3m) White Solid; mp: 132-133 °C; **^{1}H NMR** (600 MHz, $CDCl_3$) δ 7.76 (d, *J* = 7.8 Hz, 2H), 7.33-7.26 (m, 1H), 7.23 (d, *J* = 7.8 Hz, 2H), 7.26-7.21 (m, 1H), 7.14-7.02 (m, 2H), 5.84 (s, br, 2H), 5.02 (s, 1H), 2.35 (s, 3H). **^{13}C NMR** (150 MHz, $CDCl_3$) δ 162.77, (d, *J* = 248.0 Hz,), 154.47, 143.31, 141.31, 139.22 (d, *J* = 8.5 Hz), 130.71 (d, *J* = 7.5 Hz), 129.66, 126.14, 122.06, (d, *J* = 3.0 Hz,), 117.67 (d, *J* = 21.0 Hz), 113.63 (d, *J* = 23.5 Hz), 93.45, 21.54. **HRMS** (ESI) m/z calcd. For $C_{15}H_{14}FNO_2SNa$ $[M+Na]^+$ 314.0627, found 314.0629.

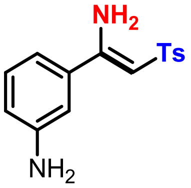

(3n) White Solid; mp: 108-109 °C; **^{1}H NMR** (600 MHz, $CDCl_3$) δ 7.82 (d, *J* = 8.4 Hz, 2H), 7.29 (d, *J* = 8.4 Hz, 2H), 7.14 (t, *J* = 7.8 Hz, 1H), 6.82 (d, *J* = 7.8 Hz, 1H), 6.74 (s, 1H), 6.72 (d, *J* = 7.8 Hz, 1H), 5.06 (s, 1H), 3.76 (s, 2H), 2.41 (s, 3H). **^{13}C NMR** (150 MHz, $CDCl_3$) δ 156.42, 146.96, 142.97, 141.76, 138.23, 129.91, 129.55, 126.01, 117.16, 116.19, 112.55, 91.87, 21.52. **HRMS** (ESI) m/z calcd. For $C_{15}H_{16}N_2O_2SNa$ $[M+Na]^+$ 311.0830, found 311.0835.

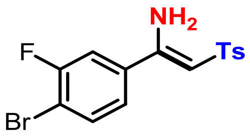

(**3o**) White Solid; mp: 127-128 °C;**^{1}H NMR** (600 MHz, $CDCl_3$) δ 7.84 (d, *J* = 7.8 Hz, 2H), 7.59 (t, *J* = 7.8 Hz, 1H), 7.33 (d, *J* = 7.8 Hz, 2H), 7.25 (d, *J* = 8.4 Hz, 1H), 7.18

(d, J = 8.4 Hz, 1H), 5.93 (s, br, 2H), 5.10 (s, 1H), 2.45 (s, 3H). **^{13}C NMR** (150 MHz, $CDCl_3$) δ 159.12, (d, J = 247.5 Hz), 153.46, 143.48, 141.07, 138.25 (d, J =7.5 Hz), 134.22, 129.71, 126.17, 123.10 (d, J =3.5 Hz), 114.63 (d, J = 24.0 Hz), 111.68 (d, J = 21.0 Hz), 93.86, 21.55. **HRMS**(ESI) m/z calcd. For $C_{15}H_{13}BrFNO_2SNa$ $[M+Na]^+$ 391.9732, found 391.9379.

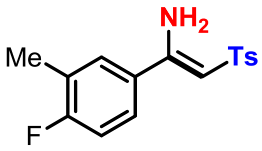

(3p) White Solid; mp: 141-142 °C; **^{1}H NMR** (600 MHz, $CDCl_3$) δ 7.82 (d, J = 8.4 Hz, 2H), 7.30 (d, J = 8.4 Hz, 2H), 7.19 (t, J = 7.8 Hz, 1H), 7.15 (dd, J = 7.8 Hz, J = 1.8 Hz, 1H), 7.11 (dd, J = 10.0 Hz, J = 1.8 Hz, 1H), 5.08 (s, 1H), 2.42 (s, 3H), 2.28 (d, J = 1.8 Hz, 3H). **^{13}C NMR** (150 MHz, $CDCl_3$) 161.20 (d, J = 244.5 Hz), 154.59, 143.17, 141.45, 136.36 (d, J = 7.5 Hz), 132.00 (d, J = 7.5 Hz), 129.60, 127.92 (d, J = 17.5 Hz), 126.09, 121.63(d, J = 3.5 Hz), 113.09 (d, J = 24.0 Hz), 92.78, 21.50, 14.46. **HRMS** (ESI) m/z calcd. For $C_{16}H_{16}FNO_2SNa$ $[M+Na]^+$ 328.0783, found 328.0785.

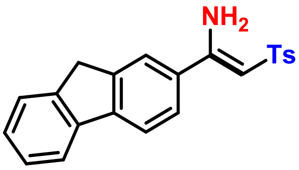

(3q) White Solid; mp: 135-136 °C; **^{1}H NMR** (500 MHz, $CDCl_3$) δ 7.86 (d, J = 8.0 Hz, 2H), 7.77 (d, J = 7.5 Hz, 1H), 7.73 (d, J = 8.0 Hz, 1H), 7.63 (s, 1H), 7.54 (d, J = 7.5 Hz, 1H), 7.47 (d, J = 8.0 Hz, 1H), 7.39 (t, J = 7.5 Hz, 1H), 7.34 (t, J = 7.5 Hz, 1H), 7.29 (d, J = 8.0 Hz, 2H), 5.82 (s, br, 2H), 5.16 (s, 1H), 3.86 (s, 2H), 2.41 (s, 3H). **^{13}C NMR** (125 MHz, $CDCl_3$) δ 156.37, 144.26, 143.67, 143.58, 142.91, 141.61, 140.41, 135.03, 129.49, 127.55, 126.92, 125.92, 125.13, 125.08, 122.95, 120.32, 120.00, 91.77, 36.74, 21.45. **HRMS** (ESI) m/z calcd. For $C_{22}H_{19}NO_2SNa$ $[M+Na]^+$ 384.1034, found 384.1038.

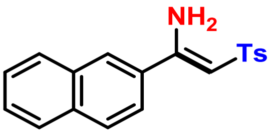

(3r) Yelloow oil; **^{1}H NMR** (600 MHz, $CDCl_3$) δ 7.97 (s, 1H), 7.87 (d, J = 7.8 Hz, 2H), 7.85-7.80 (m, 3H), 7.57-7.48 (m, 3H), 7.30 (d, J = 7.8 Hz, 2H), 6.09 (s, br, 2H), 5.22 (s, 1H), 2.42 (s, 3H). **^{13}C NMR** (150 MHz, $CDCl_3$) δ 155.98, 143.04, 141.61, 134.24, 134.16, 132.80, 129.56, 128.83, 128.46, 127.69, 127.37, 126.93, 126.13, 126.05, 123.34, 92.82, 21.48. **HRMS** (ESI) m/z calcd. For $C_{19}H_{17}NO_2SNa$ $[M+Na]^+$ 346.0878, found 346.0882.

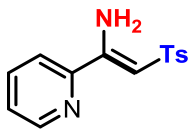

(3s) Yellow Solid; mp: 155-156 °C; **^{1}H NMR** (600 MHz, $CDCl_3$) δ 8.62-8.59 (m, 1H), 7.86 (d, *J* = 8.4 Hz, 2H), 7.75-7.70 (m, 1H), 7.64-7.60 (m, 1H), 7.37-7.33 (m, 1H), 7.28 (d, *J* = 8.4 Hz, 2H), 6.94 (m, 2H), 5.52 (s, 1H), 2.40 (s, 3H). **^{13}C NMR** (150 MHz, $CDCl_3$) δ 150.12, 150.31, 148.82, 143.18, 141.65, 137.02, 129.60, 126.14, 125.20, 120.52, 90.87, 21.53. **HRMS** (ESI) m/z calcd. For $C_{14}H_{14}N_2O_2SNa$ $[M+Na]^+$ 297.0674, found 297.0676.

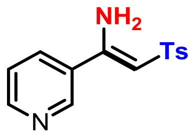

(3t) White Solid; mp: 145-146 °C; **^{1}H NMR** (600 MHz, $CDCl_3$) δ 8.72 (d, *J* = 1.8 Hz, 1H), 8.66 (d, *J* = 4.2 Hz, 1H), 7.84 (d, *J* = 8.4 Hz, 2H), 7.78 (d, *J* = 7.8 Hz, 1H), 7.35-7.30 (m, 3H), 6.02 (s, br, 2H), 5.10 (s, 1H), 2.44 (s, 3H). **^{13}C NMR** (150 MHz, $CDCl_3$) δ 152.93, 151.69, 147.46, 143.46, 141.10, 134.01, 132.90, 129.71, 126.16, 123.59, 93.97, 21.55. **HRMS** (ESI) m/z calcd. For $C_{14}H_{14}N_2O_2SNa$ $[M+Na]^+$ 297.0674, found 297.0676.

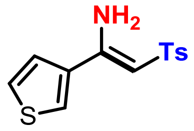

(3u) Yellow Solid; mp: 92-93 °C; **^{1}H NMR** (600 MHz, $CDCl_3$) δ 7.82 (d, *J* = 7.8 Hz, 2H), 7.37 (d, *J* = 5.4 Hz, 1H), 7.35-7.32 (m, 1H), 7.29 (d, *J* = 7.8 Hz, 2H), 7.06-7.02 (m, 1H), 5.92 (s, br, 2H), 5.27 (s, 1H), 2.41 (s, 3H). ^{13}**C NMR** (150 MHz, $CDCl_3$) δ 148.74, 143.21, 141.47, 139.01, 129.63, 127.99, 127.91, 126.58, 126.13, 92.12, 21.53. **HRMS** (ESI) m/z calcd. For $C_{13}H_{13}NO_2SNa$ $[M+Na]^+$ 302.0285, found 302.0287.

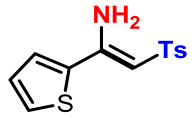

(3v) Yellow Solid; mp: 92-93 °C; **^{1}H NMR** (600 MHz, $CDCl_3$) δ 7.82 (d, *J* = 7.8 Hz, 2H), 7.53 (d, *J* = 1.8 Hz, 1H), 7.35-7.31 (m, 1H), 7.29 (d, *J* = 7.8 Hz, 2H), 7.16 (d, *J* = 1.8 Hz, 1H), 5.92 (s, br, 2H), 5.17 (s, 1H), 2.41 (s, 3H). **^{13}C NMR** (150 MHz, $CDCl_3$) δ 150.22, 143.10, 141.65, 138.13, 129.60, 127.15, 126.07, 125.39, 124.58, 92.13, 21.55. **HRMS** (ESI) m/z calcd. For $C_{13}H_{13}NO_2SNa$ $[M+Na]^+$ 302.0285, found 302.0287.

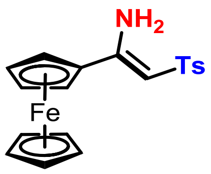

(3w) Red Solid; mp: 161-162 °C; **^{1}H NMR** (500 MHz, $CDCl_3$) δ 7.82 (d, *J* = 8.0 Hz, 2H), 7.28 (d, *J* = 8.0 Hz, 2H), 5.83 (s, br, 2H), 5.10 (s, 1H), 4.47 (t, *J* = 1.8 Hz, 2H), 4.33 (t, *J* = 1.8 Hz, 2H), 4.13 (s, 5H), 2.40 (s, 3H). **^{13}CNMR** (125 MHz, $CDCl_3$) δ 156.32, 142.77, 141.99, 129.47, 125.80, 89.20, 79.51, 70.22, 69.92, 66.65, 21.50. **HRMS** (ESI) m/z calcd. For $C_{19}H_{19}FeNO_2SNa$ $[M+Na]^+$ 404.0384, found 404.0386.

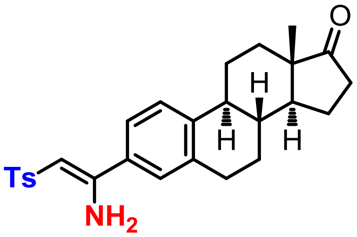

(3x) Yellow Solid; mp: 185-186 °C; **^{1}H NMR** (600 MHz, DMSO) δ 7.79 (d, *J* = 8.4 Hz, 2H), 7.37 (d, *J* = 8.4 Hz, 2H), 7.31 (d, *J* = 8.4 Hz, 1H), 7.27 (dd, *J* = 8.4 Hz, *J* = 1.8 Hz, 1H), 7.25 (d, *J* = 1.8 Hz, 1H), 7.00 (s, 2H), 5.07 (s, 1H), 2.90-2.80 (m, 2H), 2.47-2.39 (m, 1H), 2.37 (s, 3H), 2.29-2.22 (m, 1H), 2.10-2.02 (m, 1H), 1.98-1.91 (m, 2H), 1.79-1.73 (m, 1H), 1.59-1.46 (m, 3H), 1.42-1.33 (m, 3H), 0.88-0.83 (m, 1H), 0.81 (s, 3H). **^{13}CNMR** (150 MHz, DMSO) δ 219.96, 159.89, 142.94, 142.81, 142.78, 137.13, 133.63, 129.99, 127.51, 125.99, 125.97, 124.34, 89.11, 50.04, 47.71, 44.32, 37.84, 35.80, 31.74, 29.23, 26.24, 25.64, 21.58, 21.43, 13.93. **HRMS** (ESI) m/z calcd. For $C_{27}H_{31}NO_3SNa$ $[M+Na]^+$ 472.1922, found 472.1924.

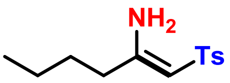

(4a) Yellow Oil ; **^{1}H NMR** (600 MHz, $CDCl_3$) δ 7.76 (d, *J* = 8.4 Hz, 2H), 7.27 (d, *J* = 8.4 Hz, 2H), 4.71 (s, 1H), 2.41 (s, 3H), 2.77 (t, *J* = 7.2 Hz, 2H), 1.52-1.45 (m, 2H), 1.33-1.28 (m, 2H), 0.88 (t, *J* = 7.2 Hz, 3H). **^{13}C NMR** (150 MHz, $CDCl_3$) δ 158.80, 142.74, 142.02, 129.46, 125.84, 90.64, 36.62, 29.66, 22.05, 21.50, 13.70. **HRMS** (ESI) m/z calcd. For $C_{13}H_{19}NO_2SNa$ $[M+Na]^+$ 276.1034, found 276.1036.

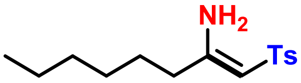

(4b) White Solid; mp: 72-73 °C; **^{1}H NMR** (600 MHz, $CDCl_3$) δ 7.76 (d, *J* = 7.8 Hz, 2H), 7.27 (d, *J* = 7.8 Hz, 2H), 4.72 (s, 1H), 2.41 (s, 3H), 2.07 (t, *J* = 7.2 Hz, 2H), 1.54 -1.45 (m, 2H), 1.31-1.20 (m, 6H), 0.85 (t, *J* = 6.6 Hz, 3H). **^{13}C NMR** (150 MHz, $CDCl_3$) δ 158.69, 142.74, 142.05, 129.46, 125.87, 90.87, 36.92, 31.41, 28.52, 27.54, 22.44, 21.49, 13.95. **HRMS** (ESI) m/z calcd. For $C_{15}H_{23}NO_2SNa$ $[M+Na]^+$ 304.1347, found 304.1349.

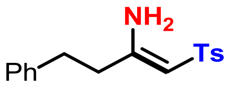

(4c) White Solid; mp: 122-123 °C; **^{1}H NMR** (600 MHz, $CDCl_3$) δ 7.71 (d, *J* = 8.4 Hz, 2H), 7.26 (d, *J* = 8.4 Hz, 2H), 7.23 (t, *J* = 7.2 Hz, 2H), 7.18 (t, *J* = 7.2 Hz, 1H), 7.09 (d, *J* = 7.2 Hz, 2H), 4.71 (s, 1H), 2.82 (t, *J* = 7.8 Hz, 2H), 2.42 (s, 3H), 2.38 (t, *J* = 7.8 Hz, 2H). **^{13}C NMR** (150 MHz, $CDCl_3$) δ 157.60, 142.80, 141.82, 139.74, 129.47, 128.63, 128.29, 126.53, 125.92, 91.38, 38.65, 33.98, 21.52. **HRMS** (ESI) m/z calcd. For $C_{17}H_{19}FNO_2SNa$ $[M+Na]^+$ 324.1034, found 324.1036.

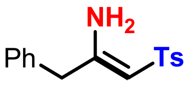

(4d) White Solid; mp: 118-119 °C; **^{1}H NMR** (600 MHz, $CDCl_3$) δ 7.77 (d, *J* = 8.4 Hz, 2H), 7.33-7.29 (m, 2H), 7.29-7.26 (m, 3H), 7.17 (d, *J* = 7.2 Hz, 2H), 4.83 (s, 1H), 3.41 (s, 2H), 2.42 (s, 3H). **^{13}C NMR** (150 MHz, $CDCl_3$) δ 156.49, 142.94, 141.79, 135.09, 129.52, 129.12, 128.99, 127.62, 125.96, 92.58, 42.61, 21.52. **HRMS** (ESI) m/z calcd. For $C_{16}H_{17}NO_2SNa$ $[M+Na]^+$ 310.0878, found 310.0880.

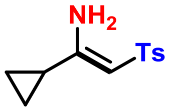

(4e) White Solid; mp: 82-83 °C; **^{1}H NMR** (600 MHz, $CDCl_3$) δ 7.75 (d, *J* = 8.4 Hz, 2H), 7.26 (d, *J* = 8.4 Hz, 2H), 5.92 (s, br, 2H), 4.65 (s, 1H), 2.40 (s, 3H), 1.47-1.31 (m, 1H), 0.88-0.76 (m, 2H), 0.73-0.63 (m, 2H). **^{13}C NMR** (150 MHz, $CDCl_3$) δ 160.08, 142.72, 142.07, 129.47, 125.83, 88.49, 21.49, 16.40, 6.91. **HRMS** (ESI) m/z calcd. For $C_{12}H_{15}NO_2SNa$ $[M+Na]^+$ 260.0721, found 260.0723.

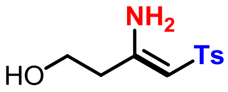

(4f) White Solid; mp: 95-96 °C; **^{1}H NMR** (600 MHz, $CDCl_3$) δ 7.77 (d, *J* = 8.4 Hz, 2H), 7.28 (d, *J* = 8.4 Hz, 2H), 4.69 (s, 1H), 3.87-3.81 (m, 2H), 2.41 (s, 3H), 2.31 (t, *J* = 5.4 Hz, 2H), 2.17 (s, 1H). **^{13}C NMR** (150 MHz, $CDCl_3$) δ 157.44, 142.96, 141.76, 129.56, 125.87, 90.72, 61.00, 39.00, 21.53. **HRMS** (ESI) m/z calcd. For $C_{11}H_{15}NO_3SNa$ $[M+Na]^+$ 264.0670, found 264.0672.

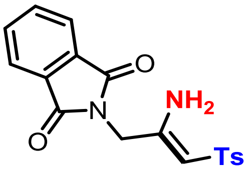

(4g) White Solid; mp: 142-143 °C; **^{1}H NMR** (500 MHz, DMSO) δ 7.92-7.87 (m, 2H), 7.87-7.83 (m, 2H), 7.69 (d, *J* = 8.0 Hz, 2H), 7.34 (d, *J* = 8.0 Hz, 2H), 6.92 (s, 2H), 4.73 (s, 1H), 4.21 (s, 2H), 2.37 (s, 3H). **^{13}C NMR** (125 MHz, DMSO) δ 167.96, 154.90, 142.84, 142.57, 134.92, 132.28, 129.92, 125.88, 123.70, 87.15, 40.41, 21.43. **HRMS** (ESI) m/z calcd. For $C_{18}H_{16}N_2O_4SNa$ $[M+Na]^+$ 379.0728, found 379.0728.

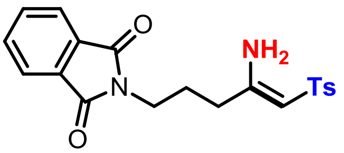

(4h) White Solid; mp: 165-166 °C; **^{1}H NMR** (600 MHz, $CDCl_3$) δ 7.84 (d, *J* = 8.4 Hz, 2H), 7.80-7.67 (m, 4H), 7.27 (d, *J* = 8.4 Hz, 2H), 4.73 (s, 1H), 3.72 (t, *J* = 7.2 Hz 2H), 2.40 (s, 3H), 2.12 (t, *J* = 7.8 Hz, 2H), 1.95-1.81 (m, 2H). **^{13}C NMR** (150 MHz, $CDCl_3$) δ 168.54, 157.58, 142.81, 141.89, 134.20, 131.93, 129.50, 125.88, 123.40, 90.99, 37.02, 33.83, 27.26, 21.50. **HRMS** (ESI) m/z calcd. For $C_{20}H_{20}N_2O_4SNa$ $[M+Na]^+$ 407.1041, found 407.1043.

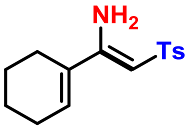

(4i) Yellow Solid; mp: 118-119 °C; **^{1}H NMR** (600 MHz, $CDCl_3$) δ 7.78 (d, *J* = 8.4 Hz, 2H), 7.27 (d, *J* = 8.4 Hz, 2H), 6.23-6.17 (m, 1H), 5.67 (s, br, 2H), 4.88 (s, 1H), 2.41 (s, 3H), 2.15-2.10 (m, 2H), 2.10-2.06 (m, 2H), 1.67-1.62 (m, 2H), 1.61-1.51 (m, 2H). **^{13}C NMR** (150 MHz, $CDCl_3$) δ 156.80, 142.80, 141.97, 133.99, 129.86, 129.49, 125.93, 90.19, 25.90, 25.50, 22.29, 21.57, 21.52. **HRMS** (ESI) m/z calcd. For $C_{15}H_{19}NO_2SNa$ $[M+Na]^+$ 300.1034, found 300.1036.

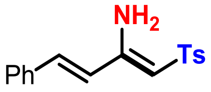

(4j) Yellow Solid; mp: 135-136 °C; **^{1}H NMR** (600 MHz, $CDCl_3$) δ 7.76 (d, *J* = 8.4 Hz, 2H), 7.50 (d, *J* = 7.2 Hz, 2H), 7.40-7.35 (m, 4H), 7.35-7.29 (m, 2H), 6.80 (s, br, 2H), 6.58 (d, *J* = 16.2 Hz, 1H), 5.11 (s, 1H), 2.37 (s, 3H). **^{13}C NMR** (150 MHz, $CDCl_3$) δ 157.55, 147.74, 147.37, 140.56, 139.43, 134.80, 134.31, 134.12, 132.40, 130.75, 129.43, 97.31, 26.19. **HRMS** (ESI) m/z calcd. For $C_{17}H_{17}NO_2SNa$ $[M+Na]^+$ 322.0878, found 322.0880.

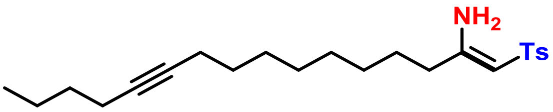

(4k) Yellow oil ; **^{1}H NMR** (600 MHz, $CDCl_3$) δ 7.76 (d, *J* = 8.4 Hz, 2H), 7.27 (d, *J* = 8.4 Hz, 2H), 4.71 (s, 1H), 2.41 (s, 3H), 2.17-2.10 (m, 4H), 2.09-2.04 (m, 2H), 1.53-1.48 (m, 2H), 1.47- 1.43 (m, 4H), 1.42-1.36 (m, 2H), 1.35-1.30 (m, 2H), 1.30 - 1.20 (m, 6H), 0.90 (t, *J* = 7.2 Hz, 3H). **^{13}C NMR** (150 MHz, $CDCl_3$) δ 158.60, 142.76, 141.97, 129.46, 125.86, 90.83, 80.27, 80.07, 36.90, 31.26, 29.12, 29.09, 28.93, 28.82, 28.71, 27.54, 21.93, 21.51, 18.72, 18.44, 13.65. **HRMS** (ESI) m/z calcd. For $C_{23}H_{35}NO_2SNa$ $[M+Na]^+$ 412.2286, found 412.2286.

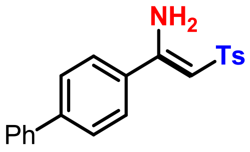

(5a) White Solid; mp: 125-125 °C; **^{1}H NMR** (500 MHz, $CDCl_3$) δ 7.85 (d, *J* = 8.0 Hz, 2H), 7.63-7.51(m, 6H), 7.45 (t, *J* = 7.0 Hz, 2H), 7.38 (t, *J* = 7.5Hz, 1H), 7.30 (d, *J* = 8.0 Hz, 2H), 6.00 (s, br, 2H), 5.16 (s, 1H), 2.42 (s, 3H). **^{13}C NMR** (125 MHz, $CDCl_3$) δ 155.57, 143.60, 143.03, 141.52, 139.72, 135.59, 129.54, 128.88, 127.94, 127.49, 127.02, 126.71, 125.98, 92.19, 21.49. **HRMS** (ESI) m/z calcd. For $C_{21}H_{19}NO_2SNa$ $[M+Na]^+$ 372.1034, found 372.1040.

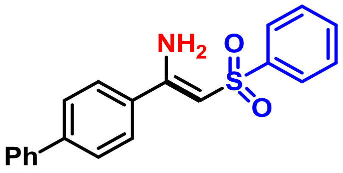

(5b) Yellow Solid; mp: 165-166 °C; **^{1}H NMR** (500 MHz, $CDCl_3$) δ 7.98 (d, *J* = 7.5 Hz, 2H), 7.64-7.48 (m, 9H), 7.45 (t, *J* = 8.0 Hz, 2H), 7.38 (t, *J* = 7.5 Hz, 1H), 6.06 (s, br, 2H), 5.16 (s, 1H). **^{13}C NMR** (125 MHz, $CDCl_3$) δ 155.96, 144.31, 143.58, 139.59, 135.38, 132.19, 128.83, 128.78, 127.86, 127.40, 126.92, 126.63, 125.80, 91.48. **HRMS** (ESI) m/z calcd. For $C_{20}H_{17}NO_2SNa$ $[M+Na]^+$ 358.0878, found 358.0882.

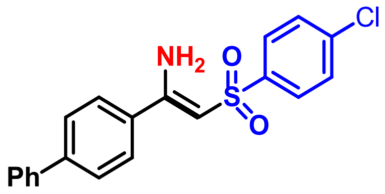

(5c) White Solid; mp: 160-161 °C; **^{1}H NMR** (500 MHz, $CDCl_3$) δ 7.90 (d, *J* = 8.5 Hz, 2H), 7.62 (d, *J* = 8.0 Hz, 2H), 7.57 (d, *J* = 7.5 Hz, 2H), 7.54 (d, *J* = 8.0 Hz, 2H), 7.50-7.43 (m, 4H), 7.38 (t, *J* = 7.1 Hz, 1H), 5.96 (s, br, 2H), 5.13 (s, 1H). **^{13}C NMR** (125 MHz, $CDCl_3$) δ 156.43, 143.91, 142.95, 139.65, 138.71, 135.32, 129.23, 128.93, 128.05, 127.61, 127.46, 127.06, 126.72, 91.25. **HRMS** (ESI) m/z calcd. For $C_{20}H_{16}ClNO_2SNa$ $[M+Na]^+$ 392.0488, found 392.0493.

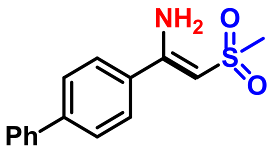

(5d) White Solid; mp: 154-155 °C; **^{1}H NMR** (600 MHz, $CDCl_3$) δ 7.58 (d, *J* = 8.4 Hz, 2H), 7.54-7.49 (m, 4H), 7.39 (t, *J* = 7.2 Hz, 2H), 7.32 (t, *J* = 7.2 Hz, 1H), 5.05 (s, 1H), 2.98 (s, 3H). **^{13}C NMR** (150 MHz, $CDCl_3$) δ 156.50, 143.84, 139.75, 135.48, 128.94, 128.03, 127.64, 127.08, 126.72, 91.19, 44.73. **HRMS** (ESI) m/z calcd. For $C_{15}H_{15}NO_2SNa$, $[M+Na]^+$ 296.0721, found 296.0725.

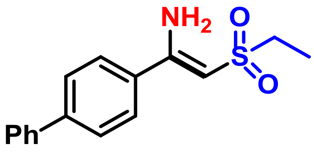

(5e) White Solid; mp: 154-155 °C; **^{1}H NMR** (600 MHz, $CDCl_3$) δ 7.66-7.63 (m, 2H), 7.61-7.57 (m, 4H), 7.46 (t, *J* = 7.2 Hz, 2H), 7.41-7.36 (m 1H), 5.90 (s, br, 2H), 4.98 (s, 1H), 3.09 (q, *J* = 7.8 Hz, 2H), 1.42 (t, *J* = 7.8 Hz, 3H). **^{13}C NMR** (150 MHz, $CDCl_3$) δ 157.54, 143.86, 139.82, 135.72, 128.98, 128.06, 127.68, 127.12, 126.76, 88.15, 51.12, 7.60. **HRMS** (ESI) m/z calcd. For $C_{16}H_{17}NO_2SNa$ $[M+Na]^+$ 310.0878, found 310.0876.

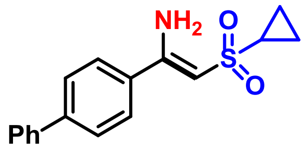

(5f) White Solid; mp: 147-148 °C; **^{1}H NMR** (500 MHz, $CDCl_3$) δ 7.66 (d, *J* = 8.5 Hz, 2H), 7.63-7.52 (m, 4H), 7.47 (t, *J* = 7.5 Hz, 2H), 7.40 (t, *J* = 7.5 Hz, 1H), 5.09 (s, 1H), 2.63-2.52 (m, 1H), 1.29-1.22 (m, 2H), 1.06-0.94 (m, 2H). **^{13}C NMR** (150 MHz, $CDCl_3$) δ 156.32, 143.74, 139.87, 135.88, 128.98, 128.04, 127.65, 127.13, 126.80, 90.18, 33.62, 4.81. **HRMS** (ESI) m/z calcd. For $C_{17}H_{17}NO_2SNa$ $[M+Na]^+$ 322.0878, found 322.0881.

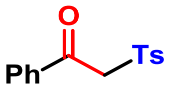

(6)[1] White Solid; mp: 107-108 °C; **^{1}H NMR** (500 MHz, $CDCl_3$) δ 7.93 (d, *J* = 8.0, Hz, 2H), 7.75 (d, *J* = 8.0 Hz, 2H), 7.64-7.57 (m, 1H), 7.46 (d, *J* = 8.0, 7.6 Hz, 2H), 7.32 (d, *J* = 8.0 Hz, 2H), 4.72 (s, 2H), 2.42 (s, 3H). **^{13}C NMR** (125 MHz, $CDCl_3$) δ 188.15, 145.35, 135.71, 134.30, 129.81, 129.30, 128.81, 128.57, 63.50, 21.70. **HRMS** (ESI) m/z calcd. For $C_{15}H_{14}O_3SNa$ $[M+Na]^+$ 297.0561, found 297.0566.

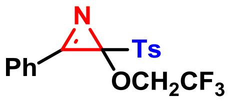

(7) Yellow oil; **^{1}H NMR** (600 MHz, $CDCl_3$) δ 7.91-7.88 (m, 2H), 7.87 (d, *J* = 8.4 Hz, 2H), 7.76-7.71 (m, 1H), 7.62 (t, *J* = 7.8 Hz, 2H), 7.43 (d, *J* = 8.4 Hz, 2H), 4.31-4.16 (m, 2H), 2.50 (s, 3H). **^{13}C NMR** (150 MHz, $CDCl_3$) δ 167.89, 145.86, 135.51, 133.88, 131.12, 130.06, 129.71, 129.26, 122.58 (q, *J* = 276.0 Hz), 120.19, 82.33, 65.85 (q, *J* = 36.0 Hz), 21.77. **^{19}F RMS** (565 MHz, $CDCl_3$) δ –74.41 (t, *J* = 8.3 Hz 3F), **HRMS** (ESI) m/z calcd. For $C_{17}H_{14}F_3NO_3SNa$ $[M+Na]^+$ 392.0544, found 392.0546.

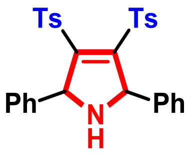

(8) White Solid; mp: 177-178 °C; **^{1}H NMR** (600 MHz, $CDCl_3$) δ 7.45 (d, *J* = 8.4 Hz, 4H), 7.4-7.38 (m, 2H), 7.34 (t, *J* = 7.8 Hz, 4H), 7.25-7.20 (m, 8H), 5.44 (s, 1H), 3.76 (s, 2H), 2.42 (s, 6H). **^{13}C NMR** (150 MHz, $CDCl_3$) δ 144.71, 143.73, 138.19, 130.01, 129.49, 128.71, 128.32, 127.50, 109.68, 26.04, 21.63. **HRMS** (ESI) m/z calcd. For $C_{30}H_{27}NO_4SNa$ $[M+Na]^+$ 552.1279, found 552.1281.

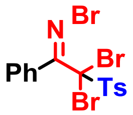

(9) White Solid; **^{1}H NMR** (500 MHz, $CDCl_3$) δ 8.02 (d, *J* = 8.0 Hz, 2H), 7.51-7.46 (m, 3H), 7.40 (d, *J* = 8.0 Hz, 2H), 7.38-7.35 (m, 2H), 2.50 (s, 3H). **^{13}C NMR** (125 MHz, $CDCl_3$) δ 176.54, 146.79, 137.74, 133.24, 130.18, 129.64, 129.16, 128.14, 127.51, 72.95, 21.90.

IV. Crystallography of 3c

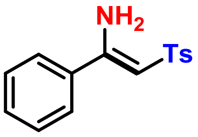

3c

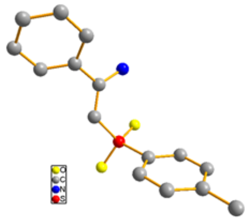

Empirical formula	C_{15} H_{14} N O_2 S
Temperature	298(2)K
Wavelength	0.71073 Å
Unit cell dimensions	a = 7.4955(18) A alpha = 90 deg.<br>b = 15.611(4) A beta = 94.333(4) deg.<br>c = 11.388(3) A gamma = 90 deg.
Volume	1226.7(2) Å ^{3}
Z	4
Calculated density	1.361 Mg/m^3
Absorption coefficient	0.240 mm^{-1}
F(000)	572
Crystal size	0.1 x 0.1x 0.1 m
Theta range for data collection	2.218 to 26.422 deg.
Reflections collected / unique	6952 / 2719 [R(int) = 0.0467]
Data / restraints / parameters	2719 / 0 / 172
Goodness-of-fit on F^2	1.173
Final R indices [I>2sigma(I)]	R1 = 0.0870, wR2 = 0.2274
R indices (all data)	R1 = 0.1089, wR2 = 0.2388

References:

1. Li, Y.; Xiang, Y.; Li, Z.; Wu, J. *Org. Chem. Front.* **2016**, *3*, 1493–1497.

2. Chen, Y.; Duan, W.-L. *J. Am. Chem. Soc.* **2013**, *135*, 16754–16757.

3. Ma, Y.;Yan, Z.; Bian, C.; Li, K.; Zhang, X.; Wang, M,.;Gao, X.; Zhang, H and Lei, A. *Chem. Commun.,* **2015**,*51*, 10524-10527.

4. Tang, X.; Huang, L.; Xu, Y.; Yang, J.; Wu, W.; Jiang, H. *Angew. Chem. Int. Ed.* **2014**, *53*, 4205−4208.

5. Sun, X.; Lyu, Y.; Zhang-Negrerie, D.; Du, Y.; Zhao, K. *Org. Lett.* **2013**, *15*, 6222–6225.

6. Gao, P.; Wang, J.; Bai, Z.; Shen, L.; Yan, Y.; Yang, D.; Fan, M.; Guan, Z. *Org. Lett.* **2016**, *18*, 6074–6077.

V. NMR Spectra Copies

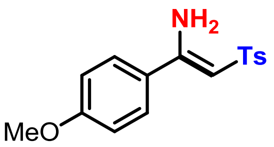
NH2
Ts
MeO

3a

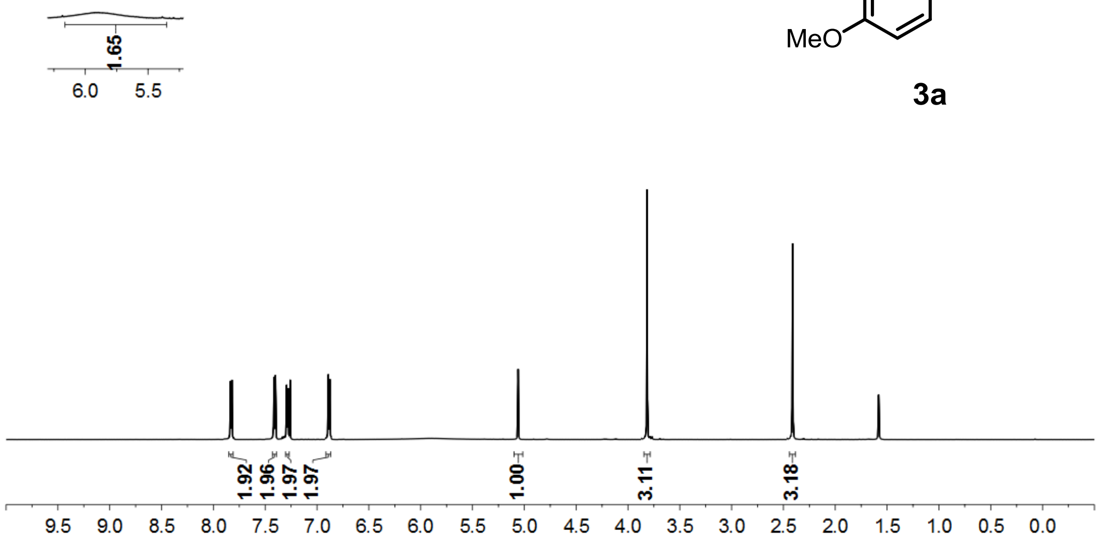
1.65
6.0
5.5
1.92
1.96
1.97
1.97
1.00
3.11
3.18
9.5 9.0 8.5 8.0 7.5 7.0 6.5 6.0 5.5 5.0 4.5 4.0 3.5 3.0 2.5 2.0 1.5 1.0 0.5 0.0

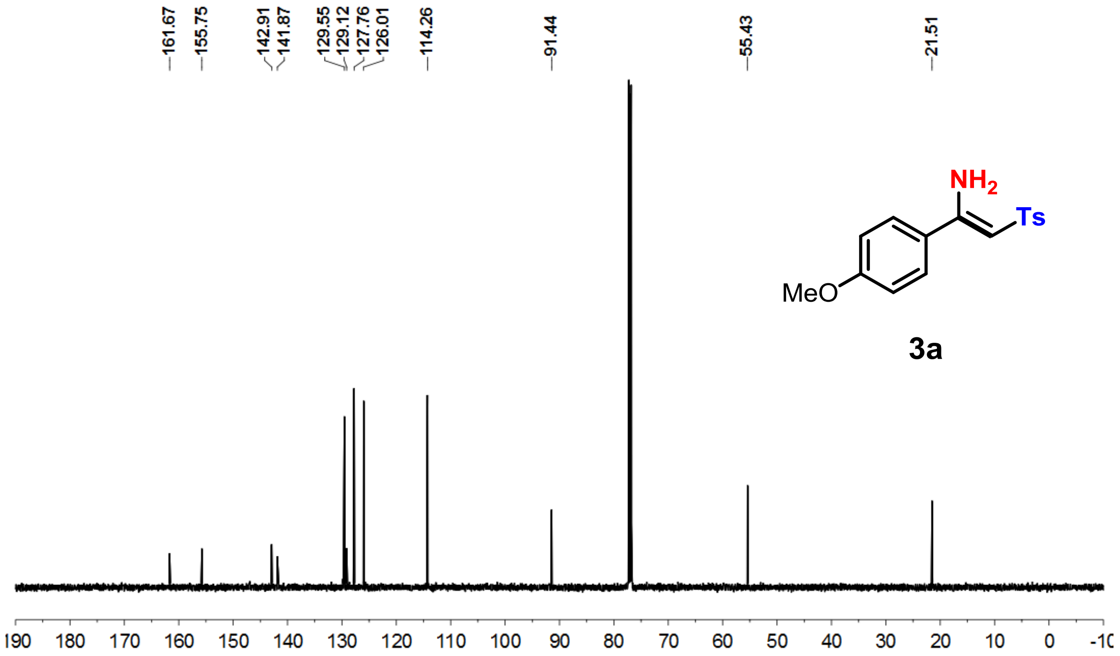
161.67
155.75
142.91
141.87
129.55
129.12
127.76
126.01
114.26
91.44
55.43
21.51
NH2
Ts
MeO
3a
190 180 170 160 150 140 130 120 110 100 90 80 70 60 50 40 30 20 10 0 -10

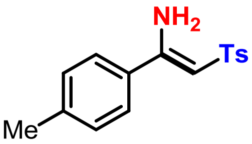
NH2
Ts
Me

3b

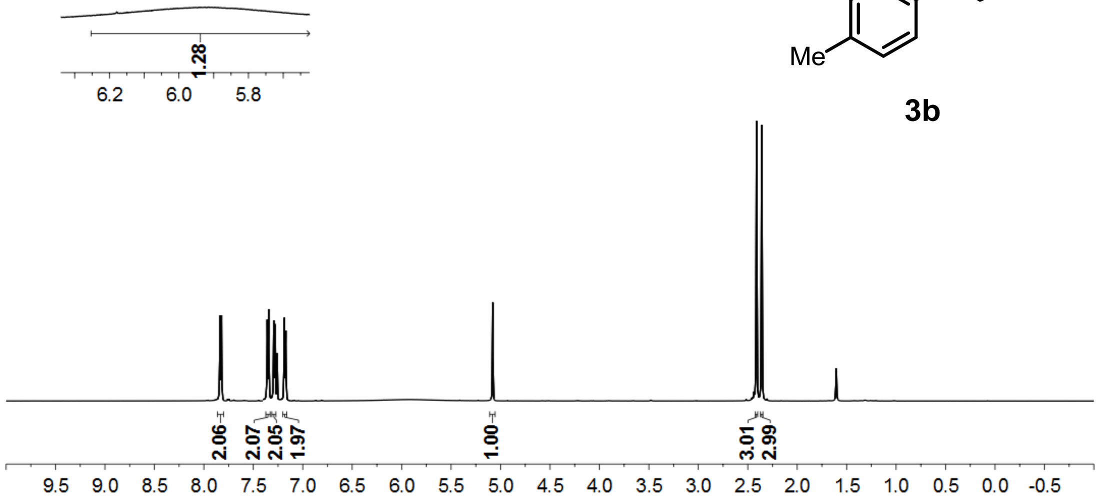
1.28
6.2 6.0 5.8
2.06
2.07
2.05
1.97
1.00
3.01
2.99
9.5 9.0 8.5 8.0 7.5 7.0 6.5 6.0 5.5 5.0 4.5 4.0 3.5 3.0 2.5 2.0 1.5 1.0 0.5 0.0 -0.5

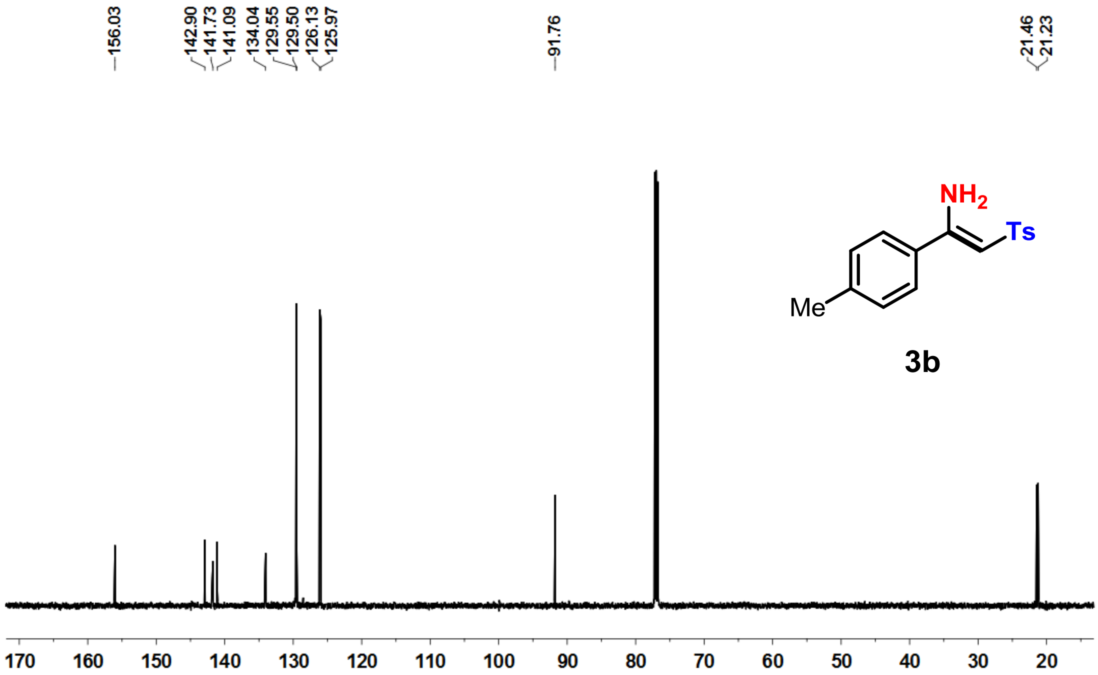
NH2
Ts
Me
3b
156.03
142.90
141.73
141.09
134.04
129.55
129.50
126.13
125.97
91.76
21.46
21.23
170 160 150 140 130 120 110 100 90 80 70 60 50 40 30 20

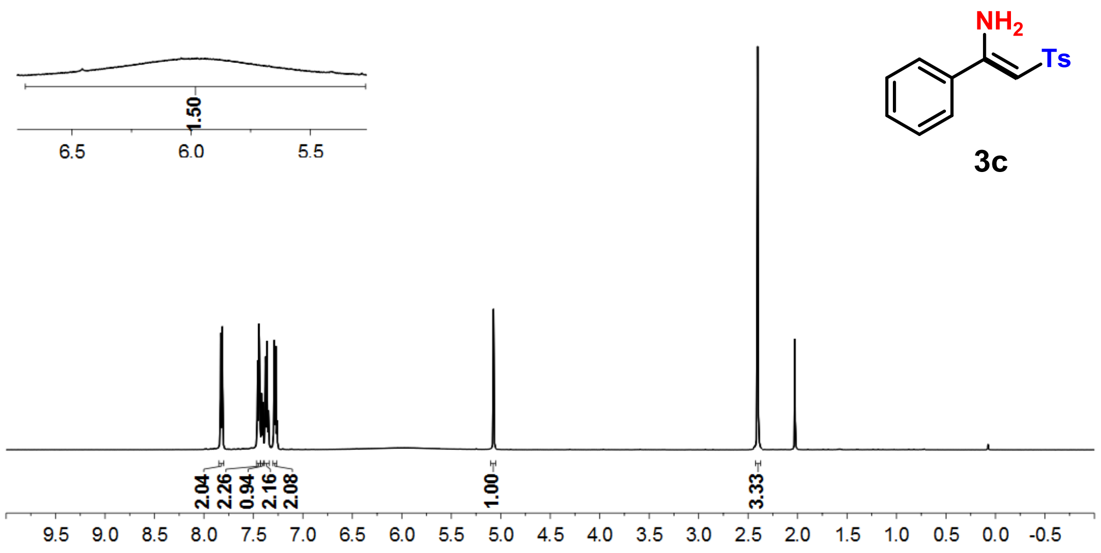
NH2
Ts
3c
1.50
6.5
6.0
5.5
2.04
2.26
0.94
2.16
2.08
1.00
3.33
9.5
9.0
8.5
8.0
7.5
7.0
6.5
6.0
5.5
5.0
4.5
4.0
3.5
3.0
2.5
2.0
1.5
1.0
0.5
0.0
-0.5

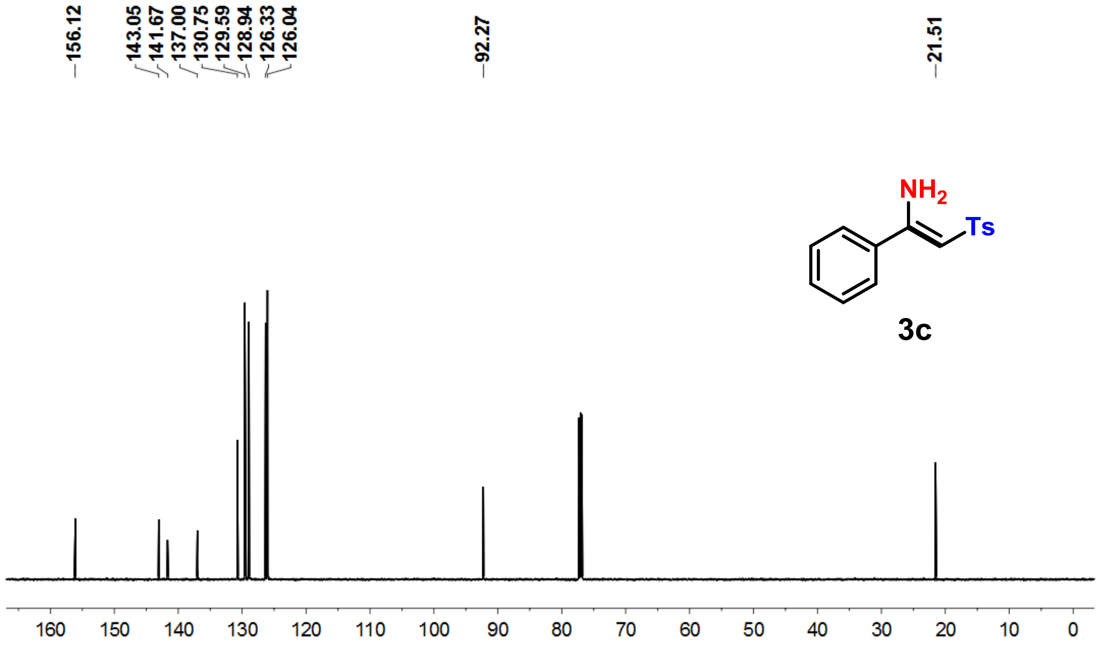
156.12
143.05
141.67
137.00
130.75
129.59
128.94
126.33
126.04
92.27
21.51
NH2
Ts
3c
160
150
140
130
120
110
100
90
80
70
60
50
40
30
20
10
0

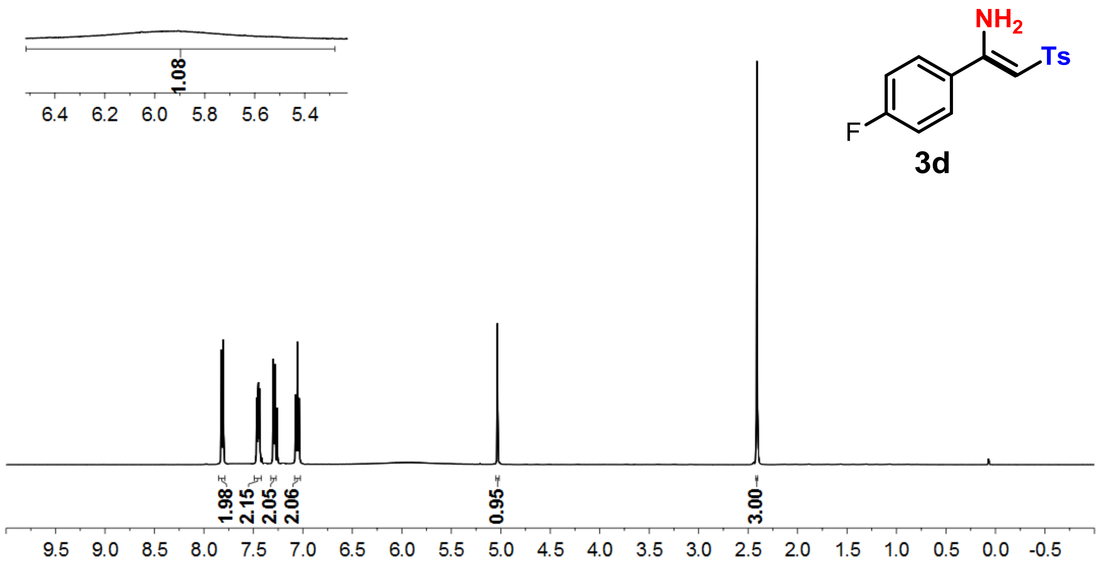

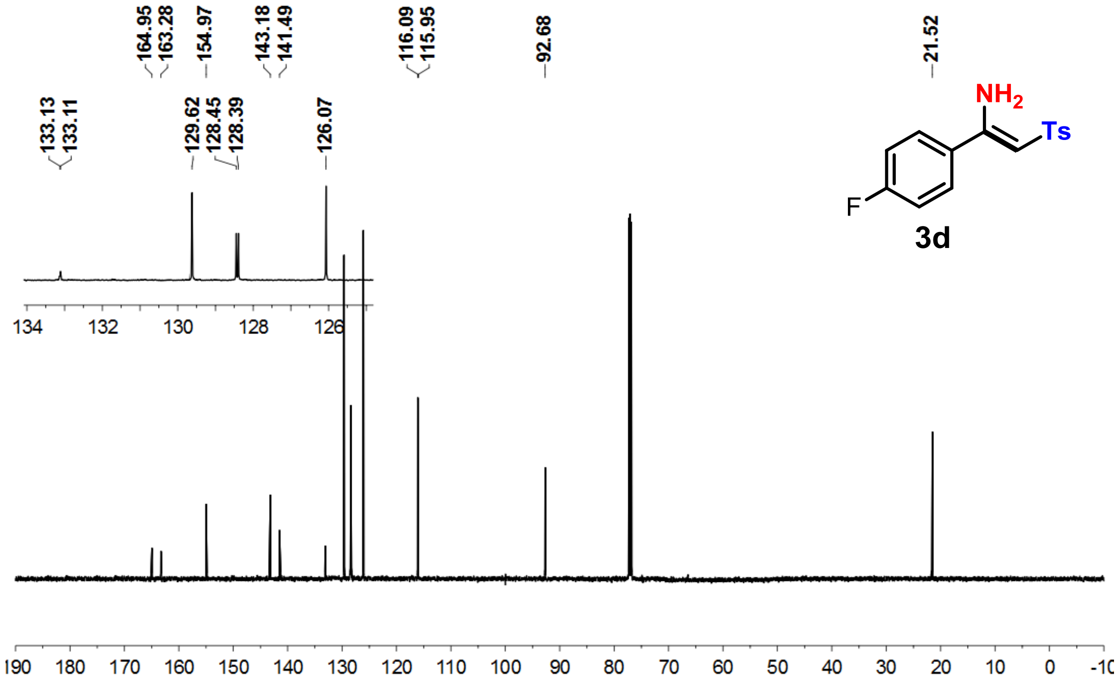

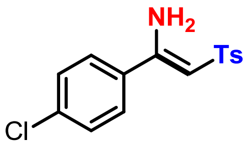

3e

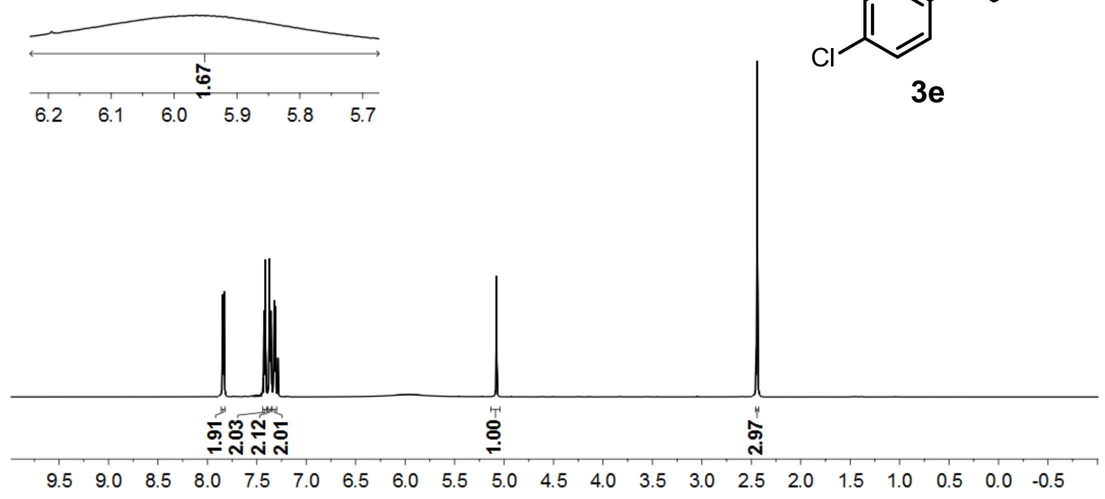
1.67
6.2 6.1 6.0 5.9 5.8 5.7
1.91 2.03 2.12 2.01
1.00
2.97
9.5 9.0 8.5 8.0 7.5 7.0 6.5 6.0 5.5 5.0 4.5 4.0 3.5 3.0 2.5 2.0 1.5 1.0 0.5 0.0 -0.5

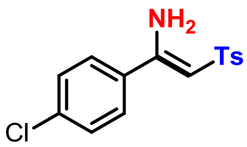

3e

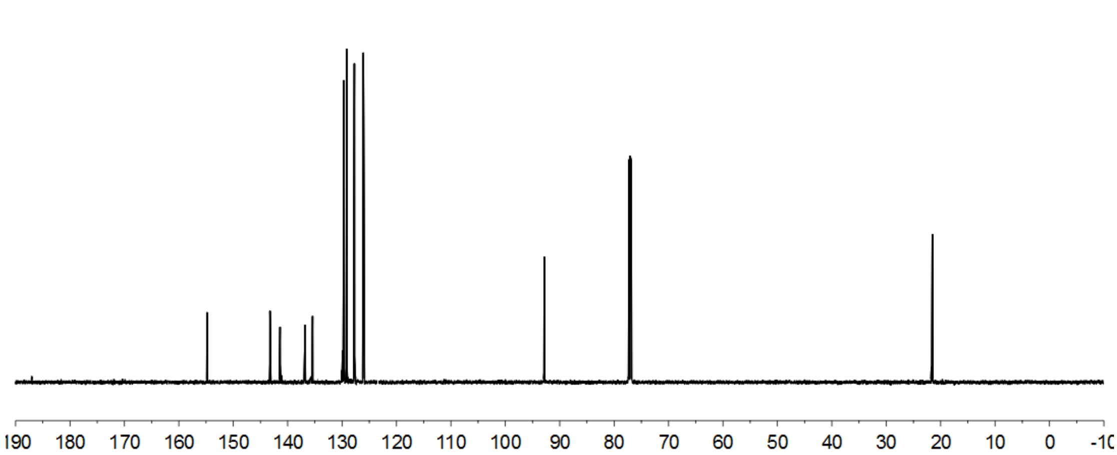
154.82
143.25 141.39 136.79 135.41 129.65 129.18 127.73 126.08
92.86
21.53
190 180 170 160 150 140 130 120 110 100 90 80 70 60 50 40 30 20 10 0 -10

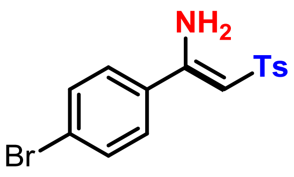

3f

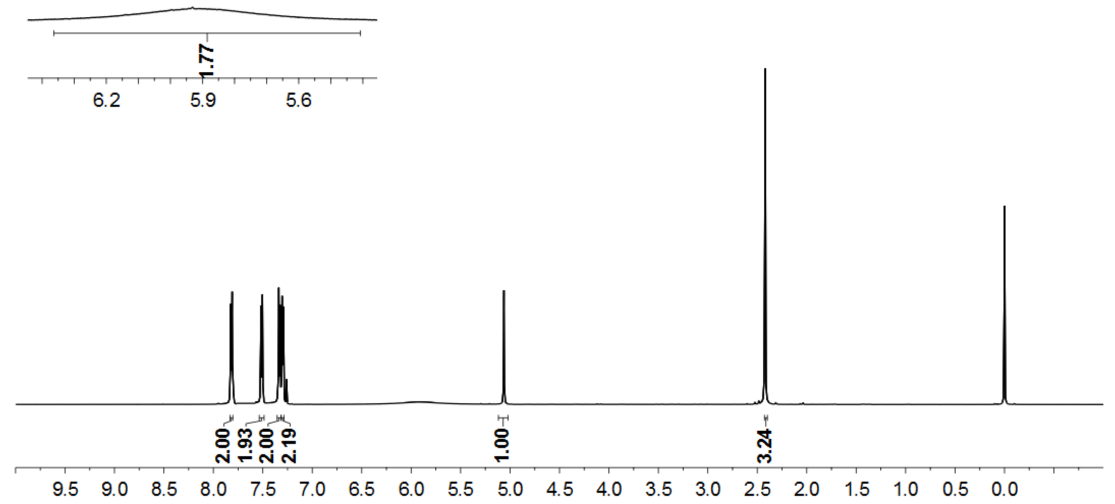
1.77
6.2
5.9
5.6
2.00
1.93
2.00
2.19
1.00
3.24
9.5 9.0 8.5 8.0 7.5 7.0 6.5 6.0 5.5 5.0 4.5 4.0 3.5 3.0 2.5 2.0 1.5 1.0 0.5 0.0

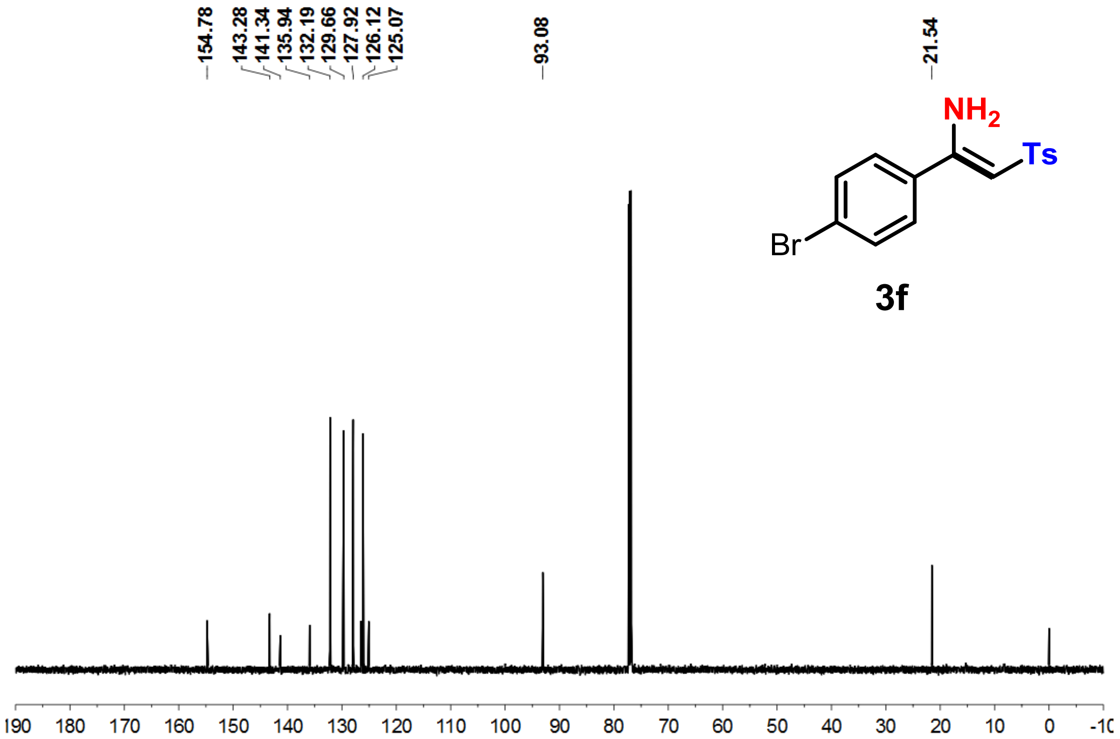
154.78
143.28
141.34
135.94
132.19
129.66
127.92
126.12
125.07
93.08
21.54
NH2
Ts
Br
3f
190 180 170 160 150 140 130 120 110 100 90 80 70 60 50 40 30 20 10 0 -10

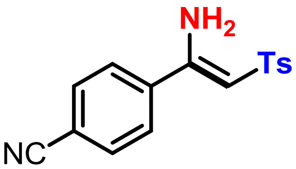
NH2
Ts
NC

3g

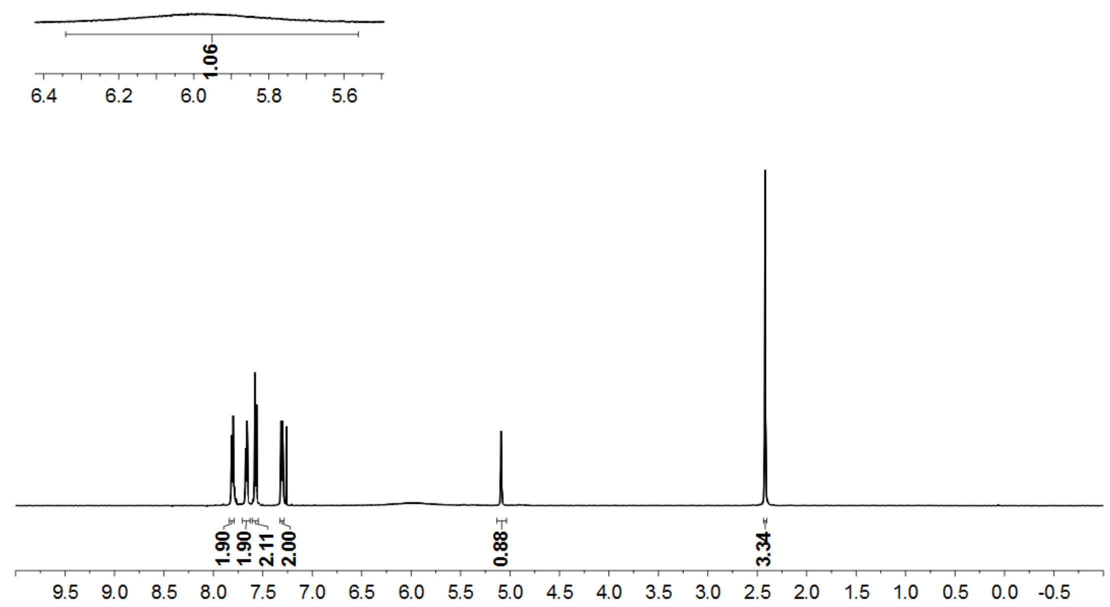
1.06
6.4 6.2 6.0 5.8 5.6
1.90 1.90 2.11 2.00
0.88
3.34
9.5 9.0 8.5 8.0 7.5 7.0 6.5 6.0 5.5 5.0 4.5 4.0 3.5 3.0 2.5 2.0 1.5 1.0 0.5 0.0 -0.5

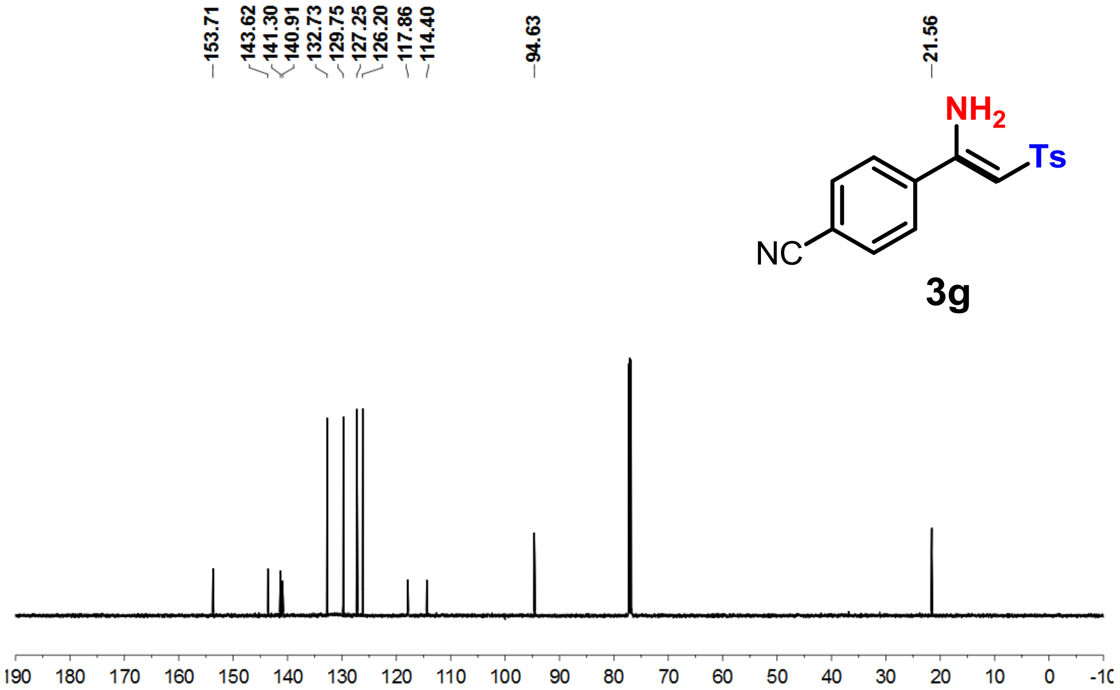
153.71
143.62 141.30 140.91 132.73 129.75 127.25 126.20 117.86 114.40
94.63
21.56
190 180 170 160 150 140 130 120 110 100 90 80 70 60 50 40 30 20 10 0 -10
NH2
Ts
NC

3g

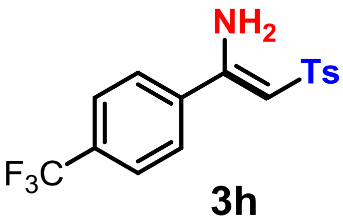
NH2
Ts
F3C

3h

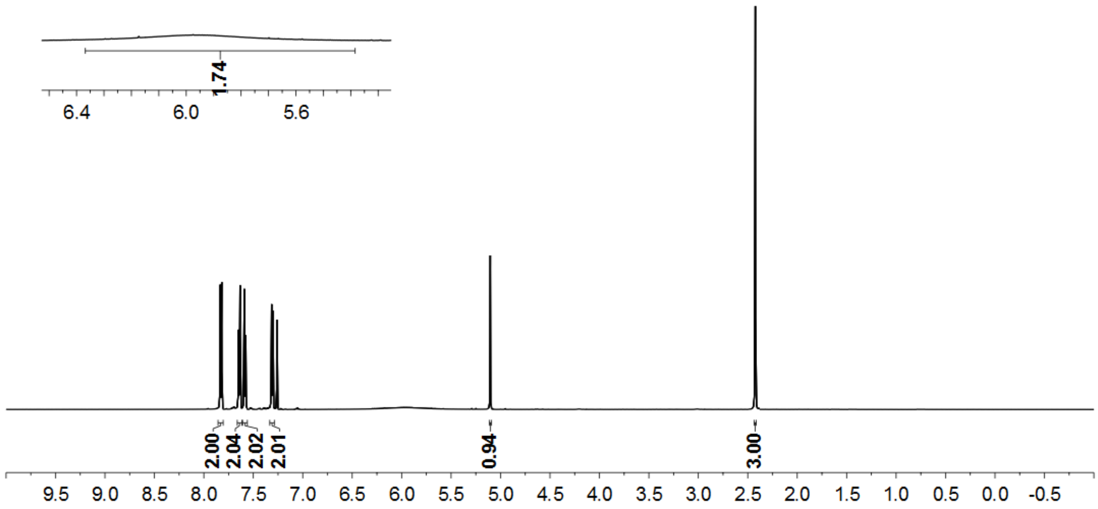
1.74
6.4
6.0
5.6
2.00
2.04
2.02
2.01
0.94
3.00
9.5
9.0
8.5
8.0
7.5
7.0
6.5
6.0
5.5
5.0
4.5
4.0
3.5
3.0
2.5
2.0
1.5
1.0
0.5
0.0
-0.5

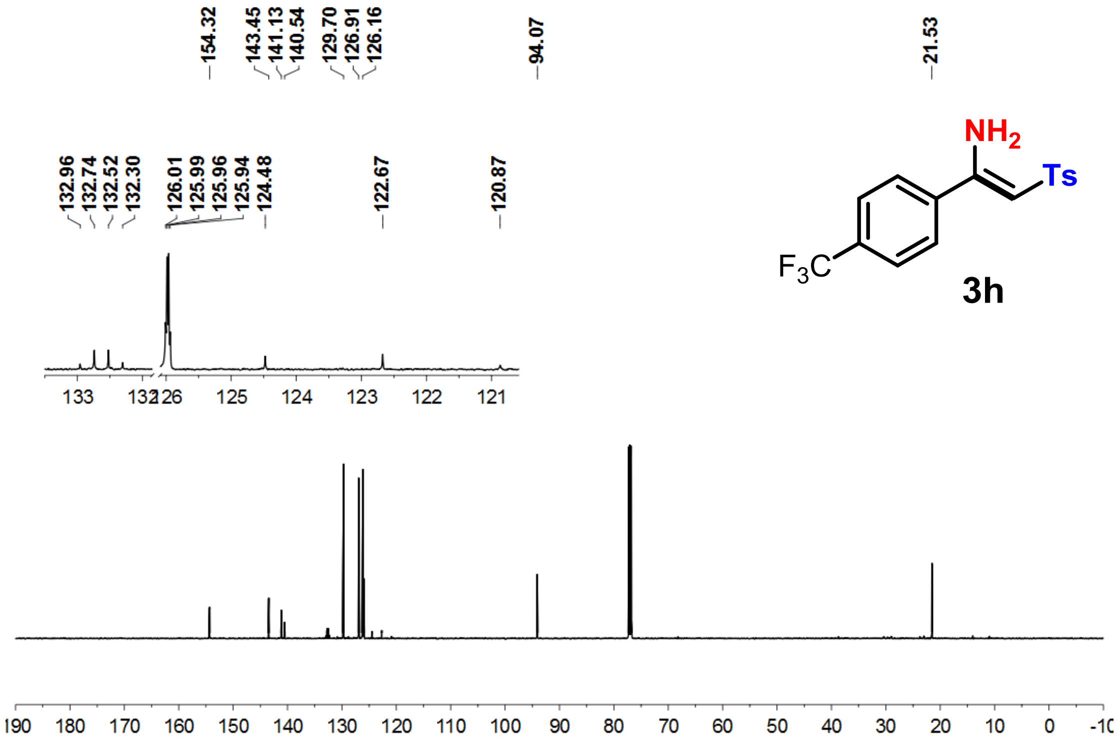
NH2
Ts
F3C
3h
154.32
143.45
141.13
140.54
129.70
126.91
126.16
94.07
21.53
132.96
132.74
132.52
132.30
126.01
125.99
125.96
125.94
124.48
122.67
120.87
133
132
126
125
124
123
122
121
190
180
170
160
150
140
130
120
110
100
90
80
70
60
50
40
30
20
10
0
-10

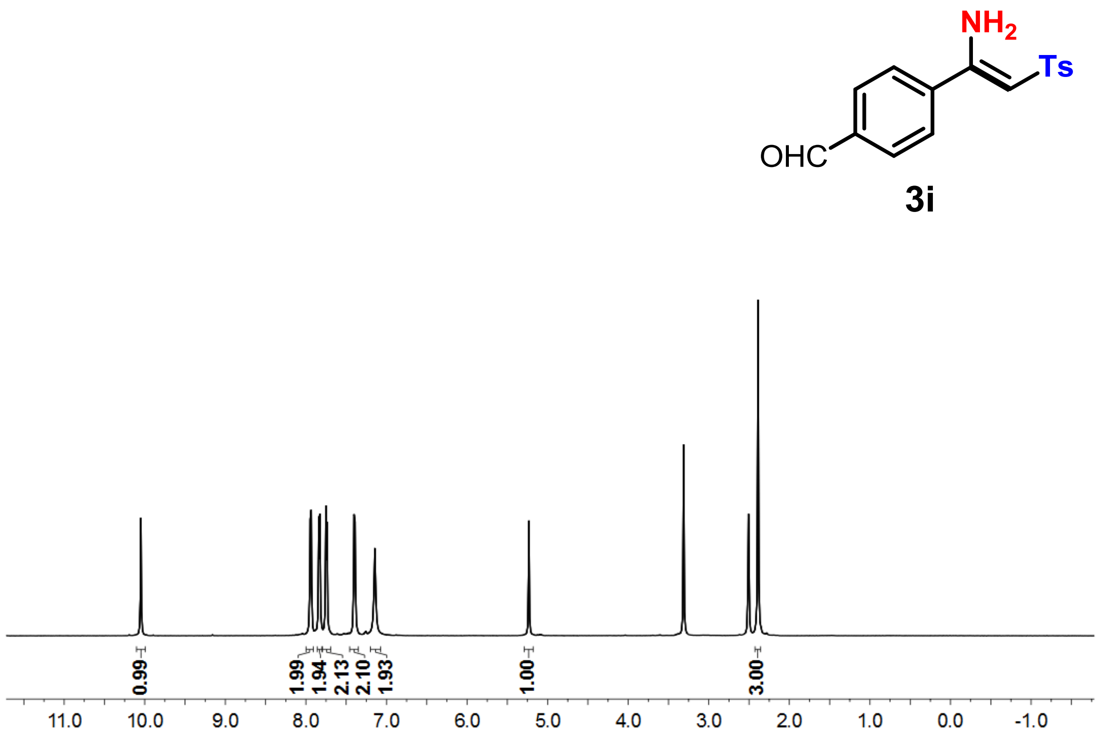
NH2
Ts
OHC
3i
0.99
1.99
1.94
2.13
2.10
1.93
1.00
3.00
11.0 10.0 9.0 8.0 7.0 6.0 5.0 4.0 3.0 2.0 1.0 0.0 -1.0

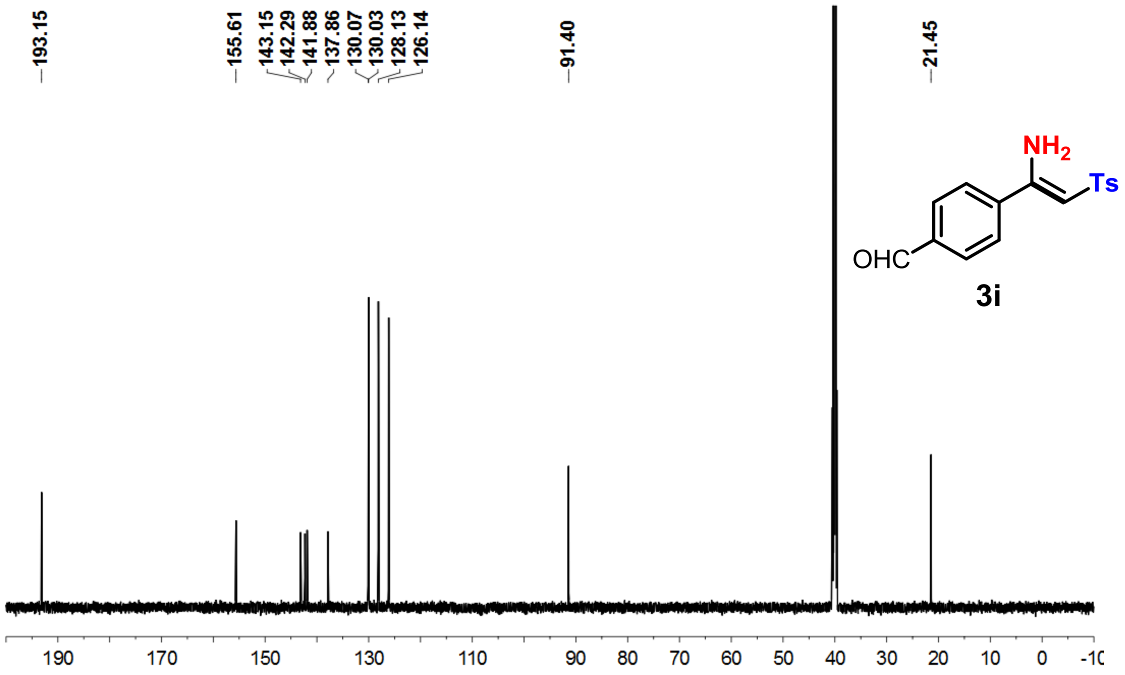
193.15
155.61
143.15
142.29
141.88
137.86
130.07
130.03
128.13
126.14
91.40
21.45
NH2
Ts
OHC
3i
190 170 150 130 110 90 80 70 60 50 40 30 20 10 0 -10

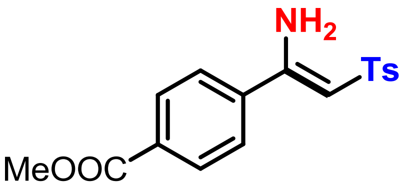
NH2
Ts
MeOOC

3j

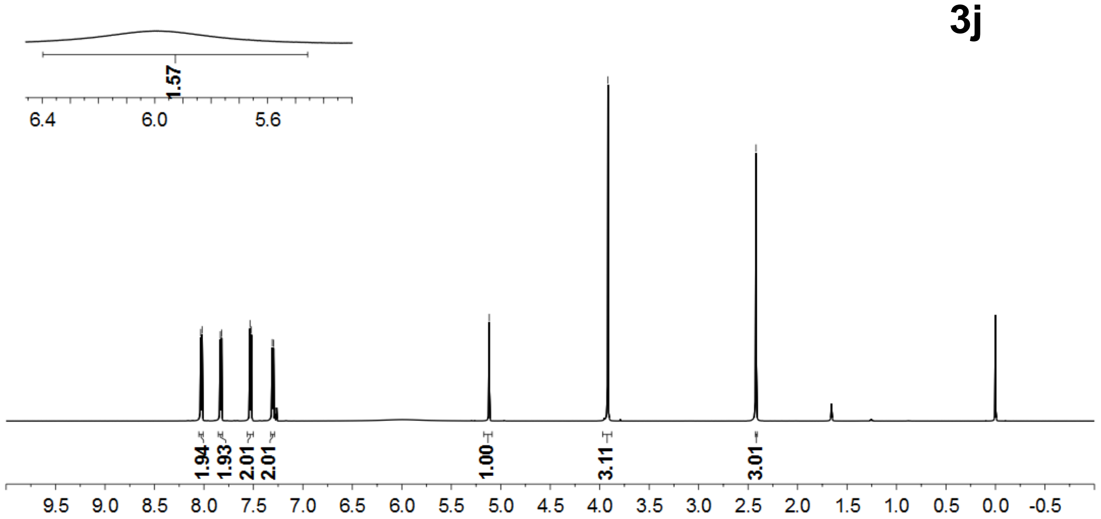
1.57
6.4
6.0
5.6
1.94
1.93
2.01
2.01
1.00
3.11
3.01
9.5 9.0 8.5 8.0 7.5 7.0 6.5 6.0 5.5 5.0 4.5 4.0 3.5 3.0 2.5 2.0 1.5 1.0 0.5 0.0 -0.5

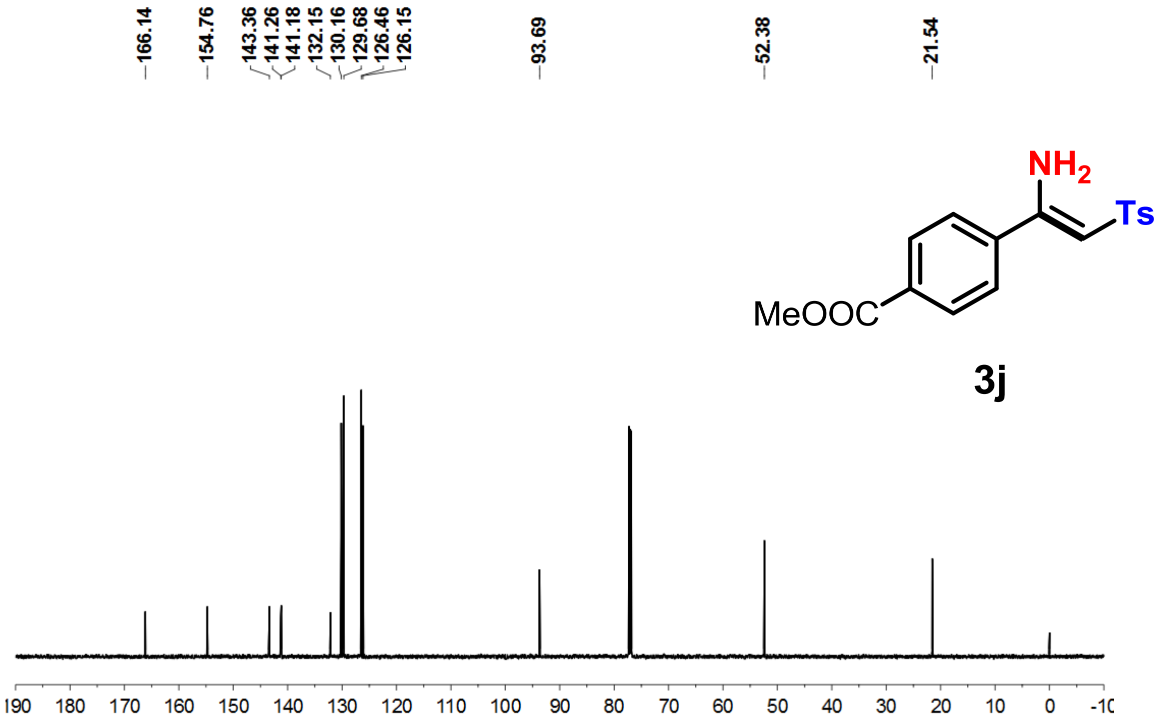
166.14
154.76
143.36
141.26
141.18
132.15
130.16
129.68
126.46
126.15
93.69
52.38
21.54
190 180 170 160 150 140 130 120 110 100 90 80 70 60 50 40 30 20 10 0 -10
NH2
Ts
MeOOC

3j

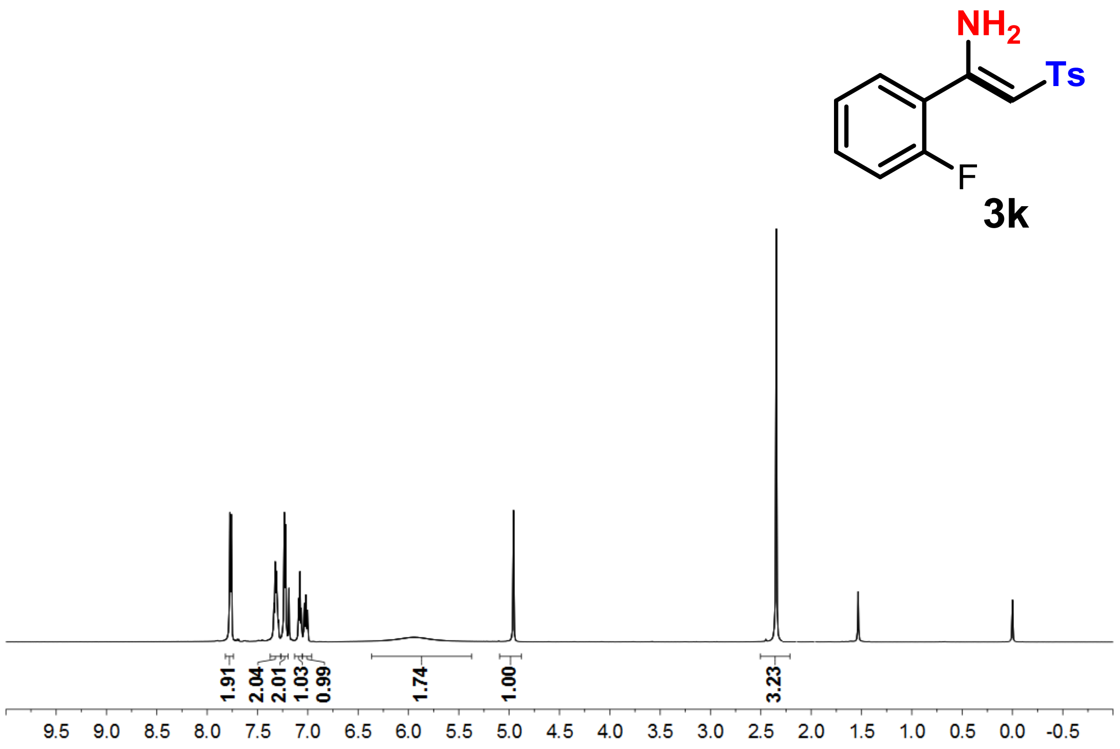
NH2
Ts
F
3k
1.91
2.04
2.01
1.03
0.99
1.74
1.00
3.23
9.5 9.0 8.5 8.0 7.5 7.0 6.5 6.0 5.5 5.0 4.5 4.0 3.5 3.0 2.5 2.0 1.5 1.0 0.5 0.0 -0.5

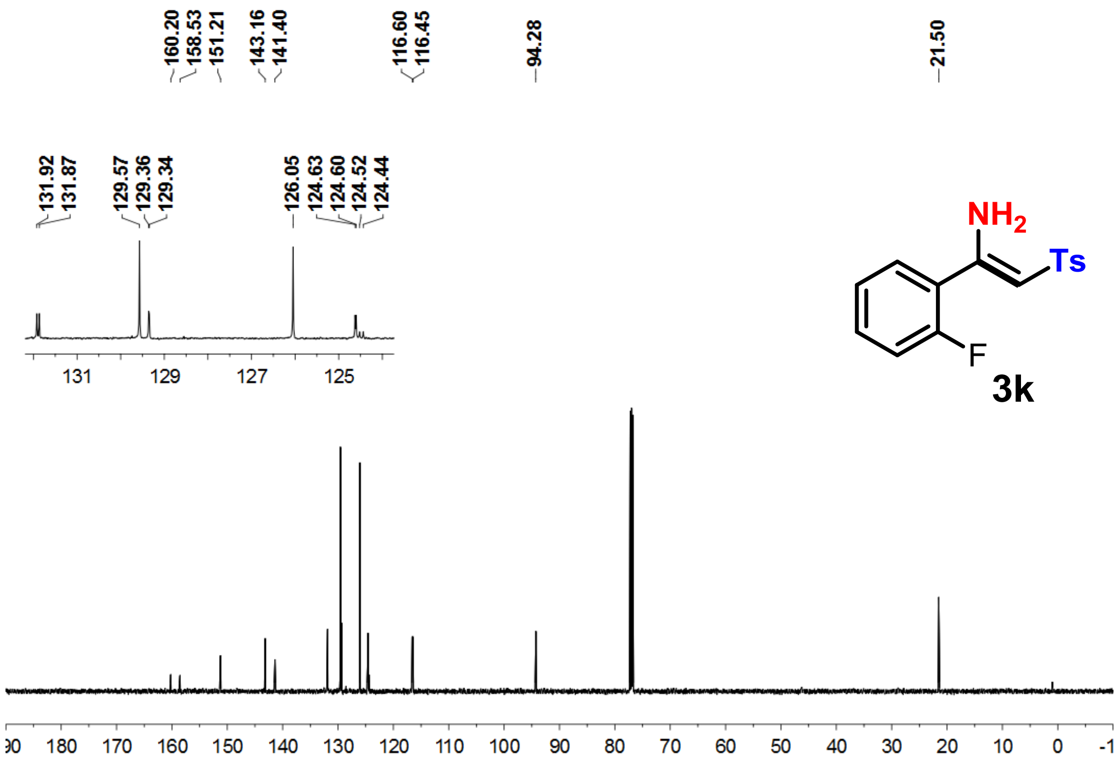
160.20
158.53
151.21
143.16
141.40
116.60
116.45
94.28
21.50
131.92
131.87
129.57
129.36
129.34
126.05
124.63
124.60
124.52
124.44
131 129 127 125
NH2
Ts
F
3k
180 170 160 150 140 130 120 110 100 90 80 70 60 50 40 30 20 10 0

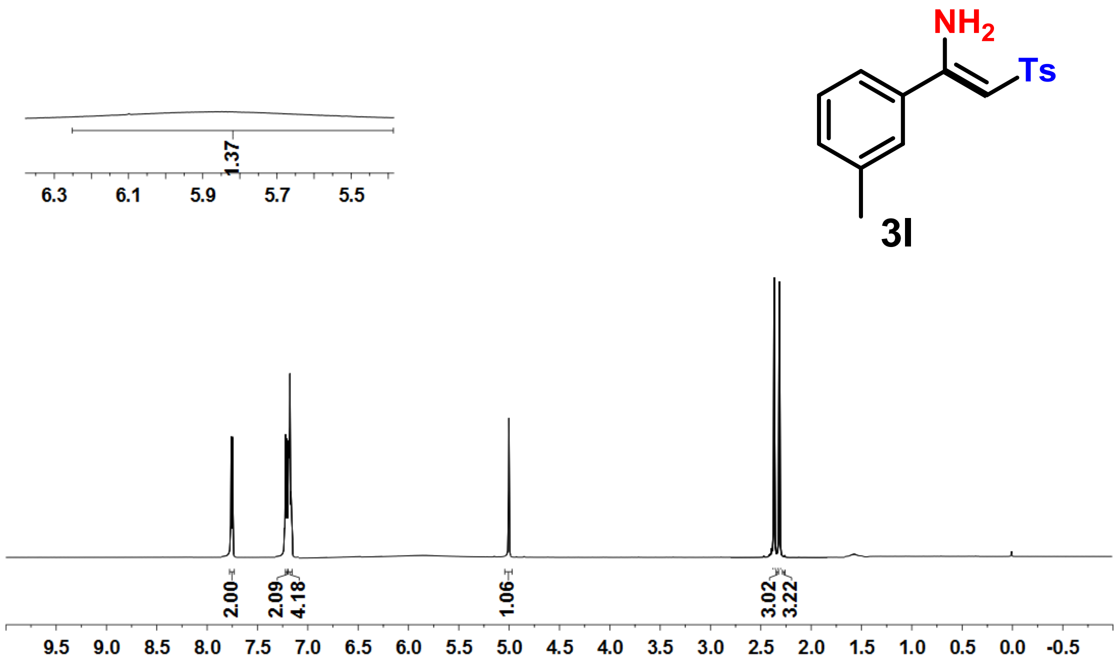
NH2
Ts
3l

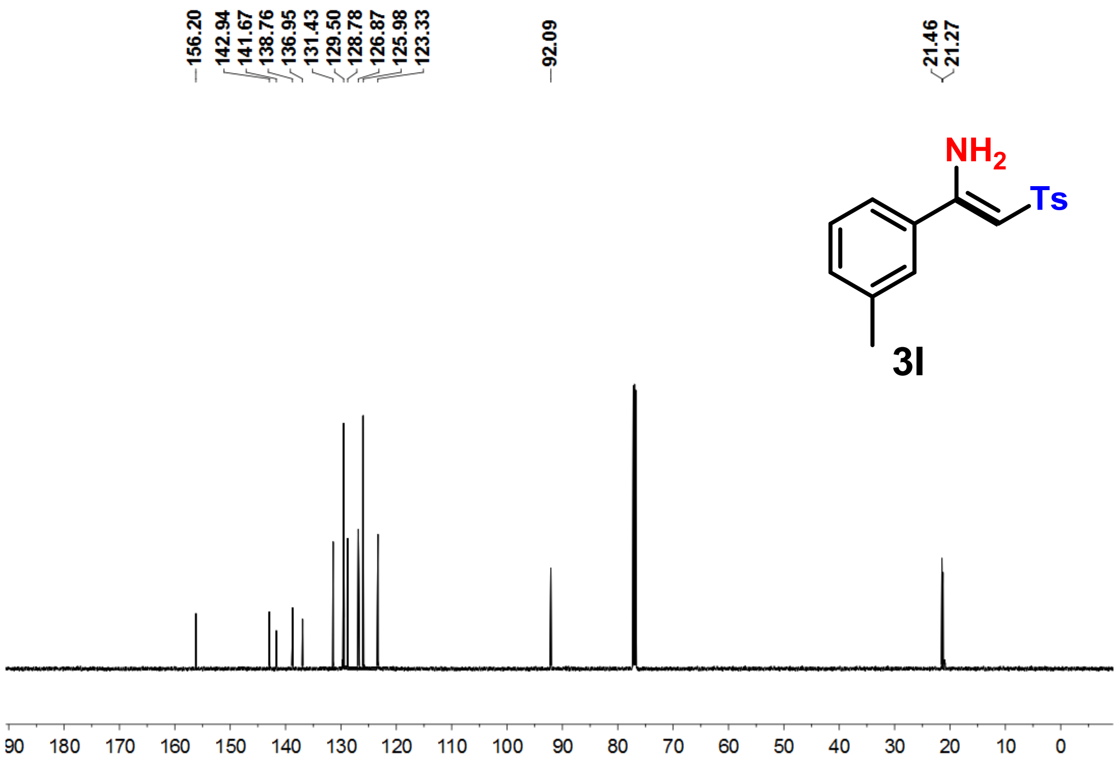
NH2
Ts
3l

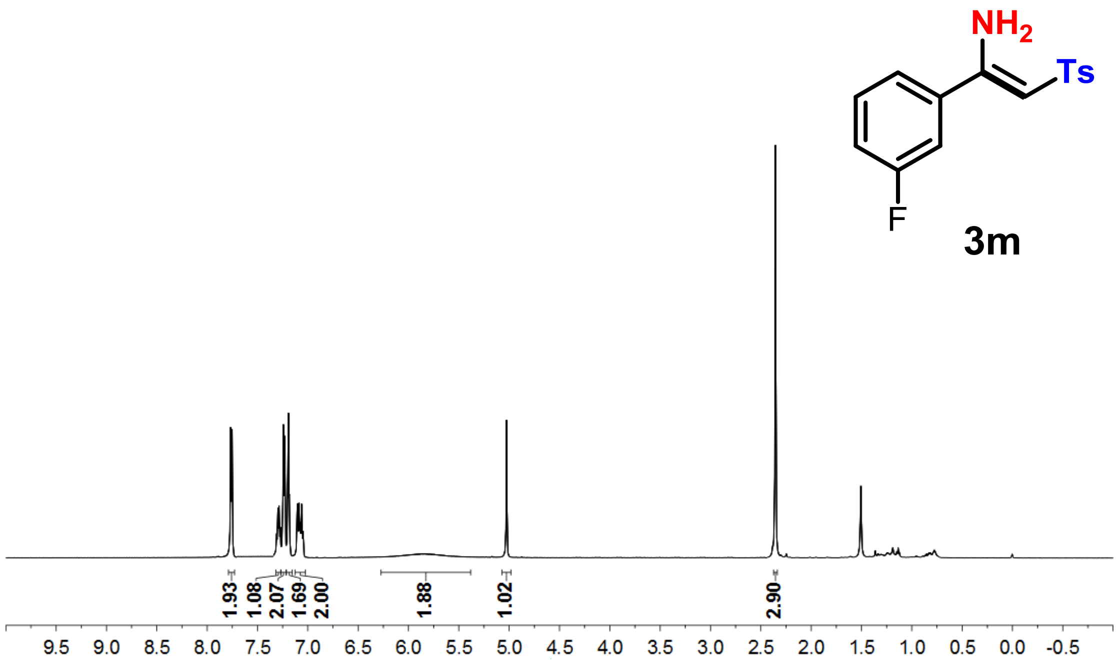

NH2
Ts
F
3m
1.93
1.08
2.07
1.69
2.00
1.88
1.02
2.90
9.5 9.0 8.5 8.0 7.5 7.0 6.5 6.0 5.5 5.0 4.5 4.0 3.5 3.0 2.5 2.0 1.5 1.0 0.5 0.0 -0.5

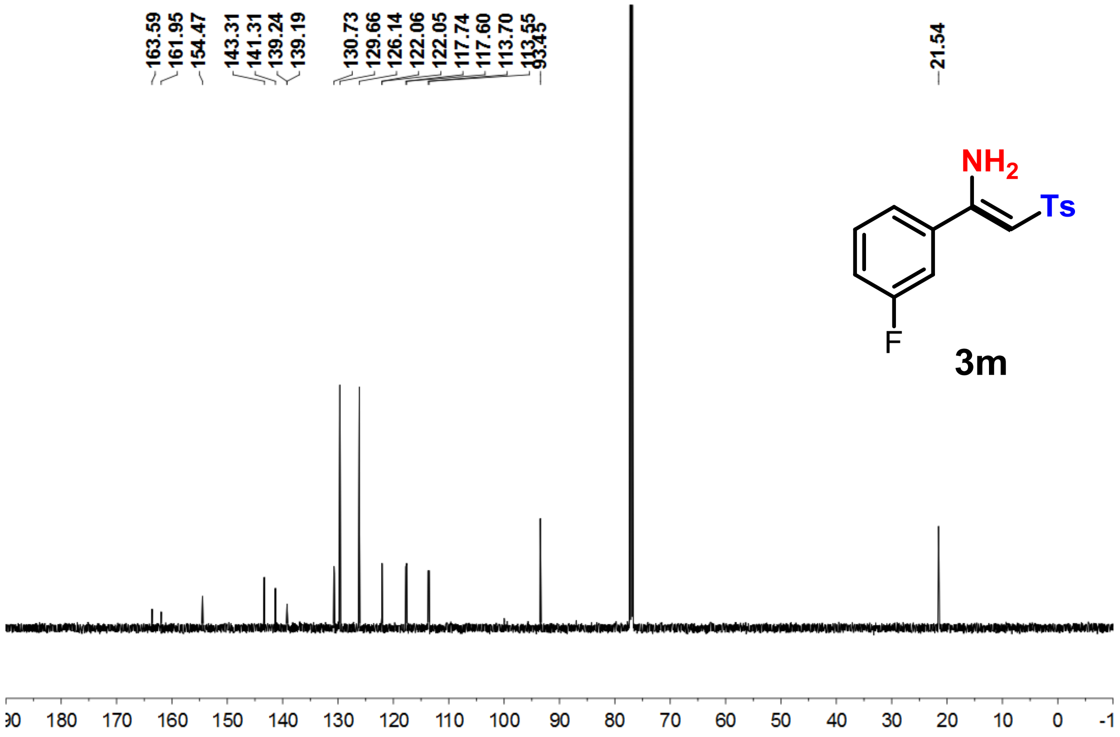

163.59
161.95
154.47
143.31
141.31
139.24
139.19
130.73
129.66
126.14
122.06
122.05
117.74
117.60
113.70
113.55
93.45
21.54
NH2
Ts
F
3m
180 170 160 150 140 130 120 110 100 90 80 70 60 50 40 30 20 10 0

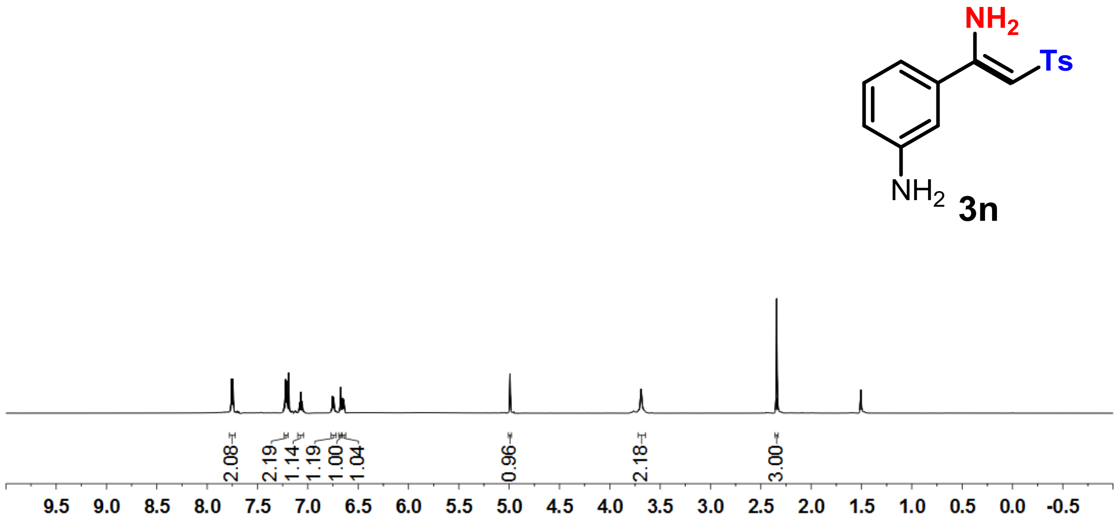
NH2
Ts
NH2
3n
2.08
2.19
1.14
1.19
1.00
1.04
0.96
2.18
3.00
9.5 9.0 8.5 8.0 7.5 7.0 6.5 6.0 5.5 5.0 4.5 4.0 3.5 3.0 2.5 2.0 1.5 1.0 0.5 0.0 -0.5

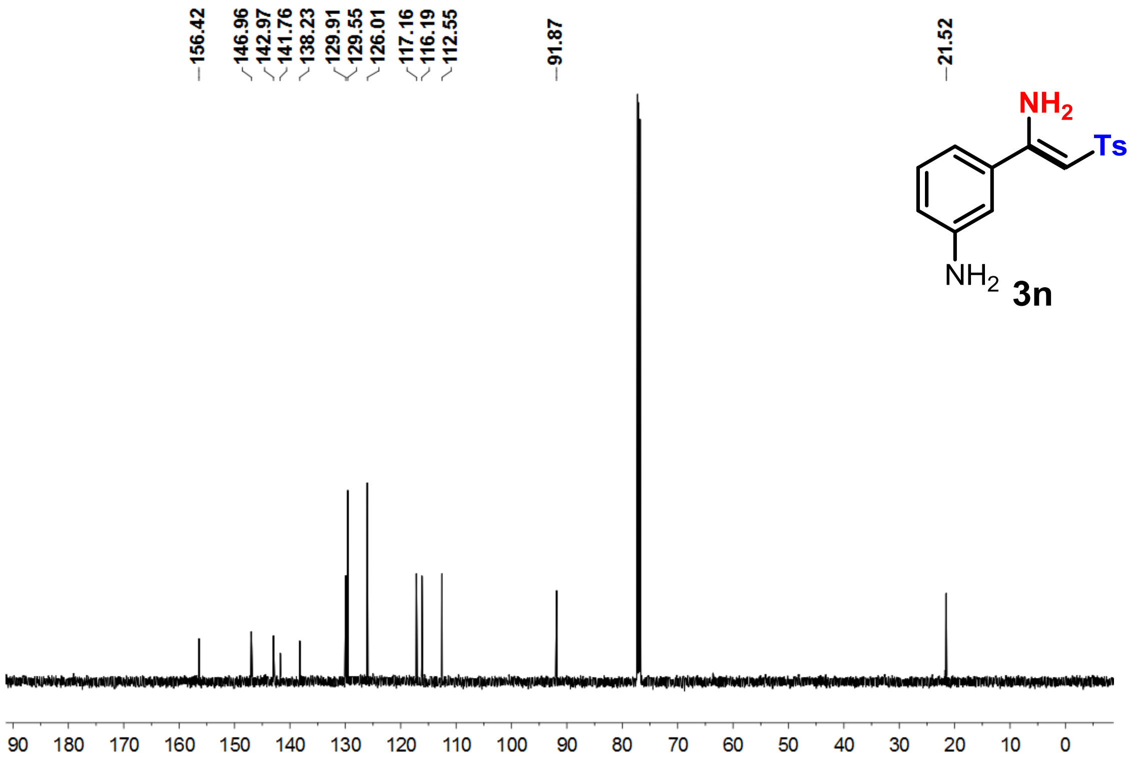
156.42
146.96
142.97
141.76
138.23
129.91
129.55
126.01
117.16
116.19
112.55
91.87
21.52
NH2
Ts
NH2
3n
90 180 170 160 150 140 130 120 110 100 90 80 70 60 50 40 30 20 10 0

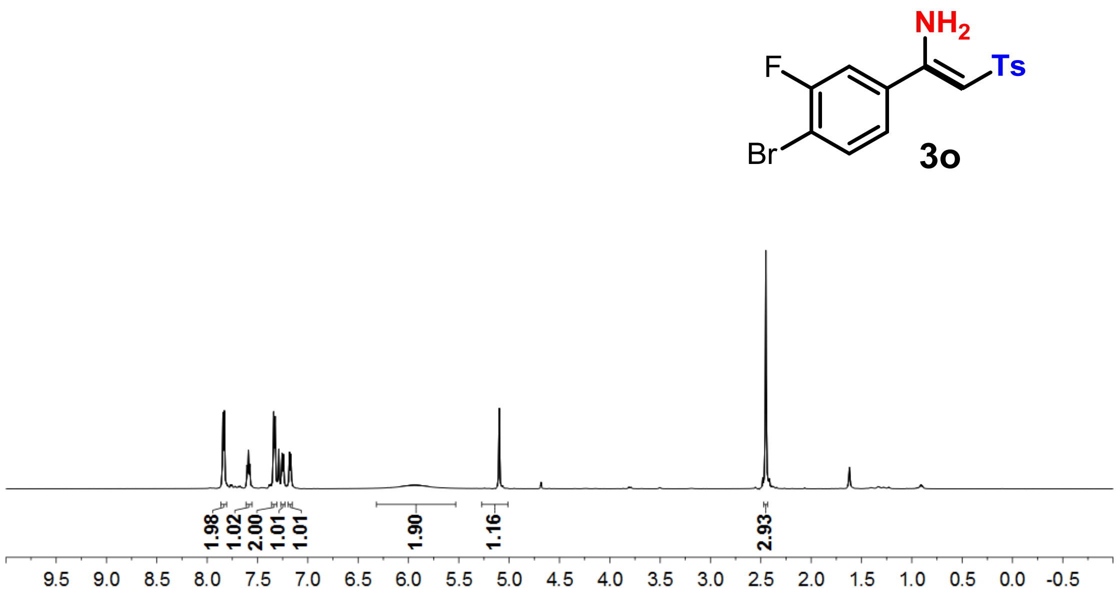
NH2
F
Ts
Br
3o
1.98
1.02
2.00
1.01
1.01
1.90
1.16
2.93
9.5 9.0 8.5 8.0 7.5 7.0 6.5 6.0 5.5 5.0 4.5 4.0 3.5 3.0 2.5 2.0 1.5 1.0 0.5 0.0 -0.5

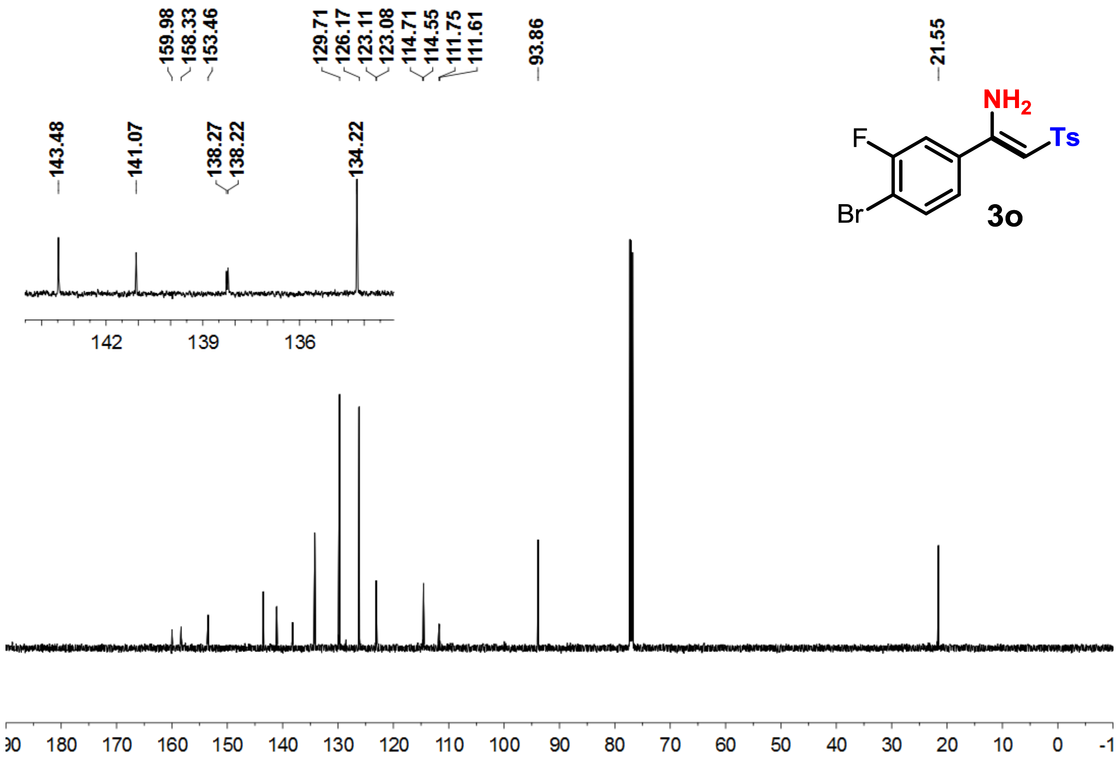
159.98
158.33
153.46
129.71
126.17
123.11
123.08
114.71
114.55
111.75
111.61
93.86
21.55
143.48
141.07
138.27
138.22
134.22
142 139 136
NH2
F
Ts
Br
3o
180 170 160 150 140 130 120 110 100 90 80 70 60 50 40 30 20 10 0 -1

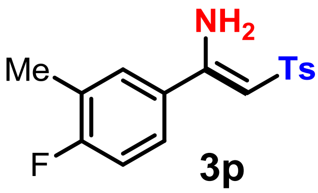
NH2
Ts
Me
F

3p

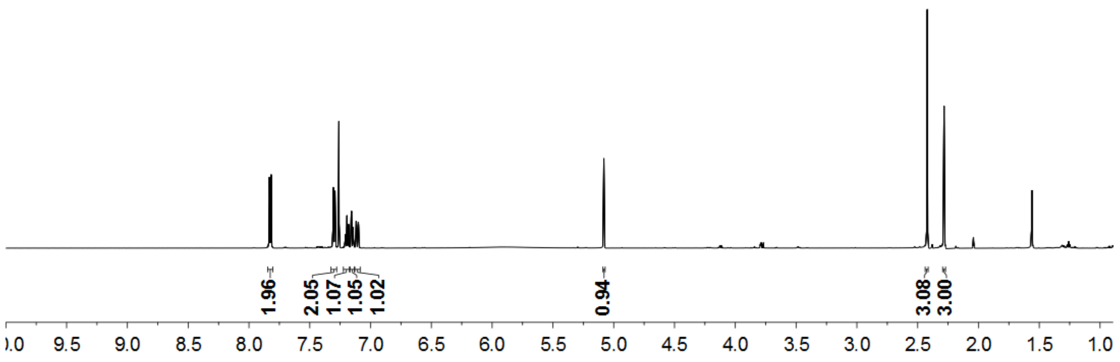
1.96
2.05
1.07
1.05
1.02
0.94
3.08
3.00
9.5 9.0 8.5 8.0 7.5 7.0 6.5 6.0 5.5 5.0 4.5 4.0 3.5 3.0 2.5 2.0 1.5 1.0

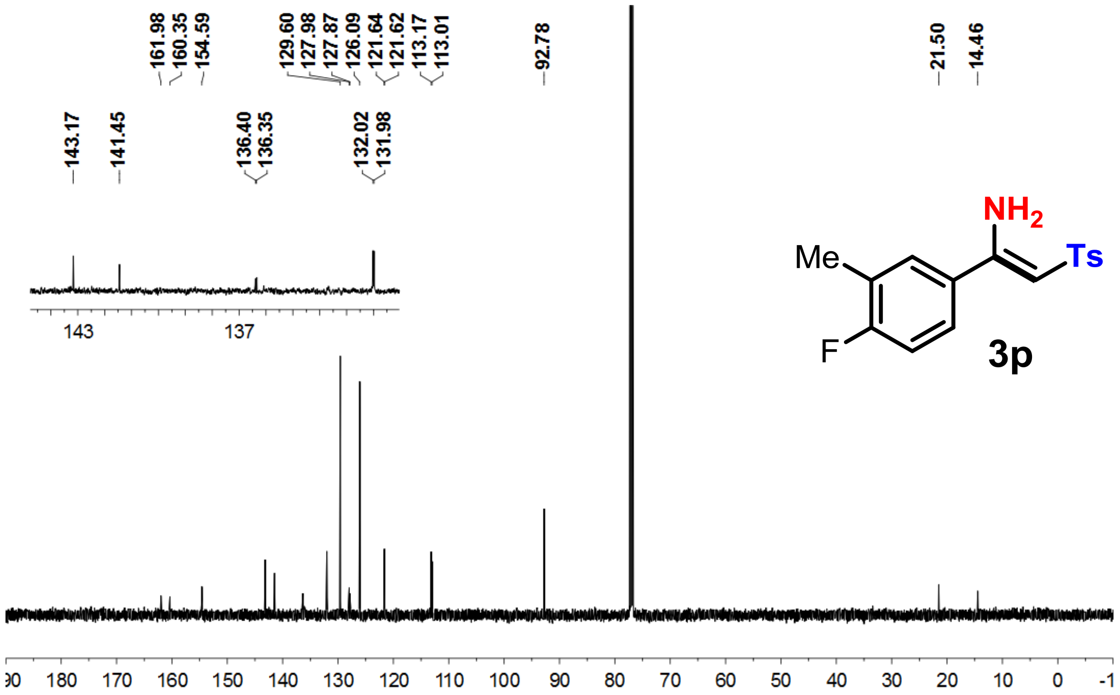
161.98
160.35
154.59
129.60
127.98
127.87
126.09
121.64
121.62
113.17
113.01
92.78
21.50
14.46
143.17
141.45
136.40
136.35
132.02
131.98
143
137
NH2
Ts
Me
F
3p
180 170 160 150 140 130 120 110 100 90 80 70 60 50 40 30 20 10 0

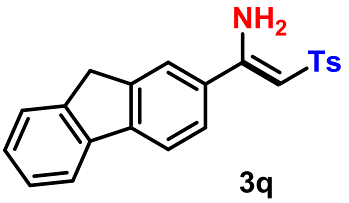
NH2
Ts

3q

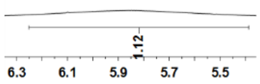
1.12
6.3 6.1 5.9 5.7 5.5

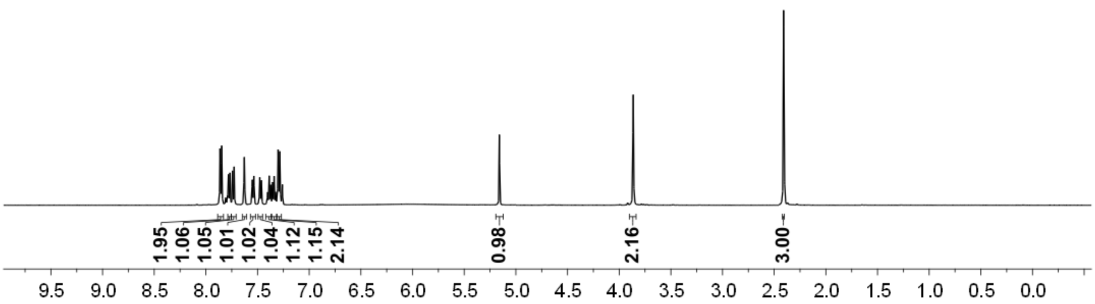
1.95 1.06 1.05 1.01 1.02 1.04 1.12 1.15 2.14
0.98
2.16
3.00
9.5 9.0 8.5 8.0 7.5 7.0 6.5 6.0 5.5 5.0 4.5 4.0 3.5 3.0 2.5 2.0 1.5 1.0 0.5 0.0

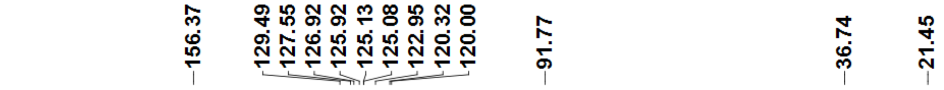
156.37
129.49 127.55 126.92 125.92 125.13 125.08 122.95 120.32 120.00
91.77
36.74
21.45

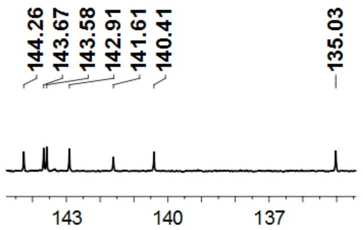
144.26 143.67 143.58 142.91 141.61 140.41
135.03
143 140 137

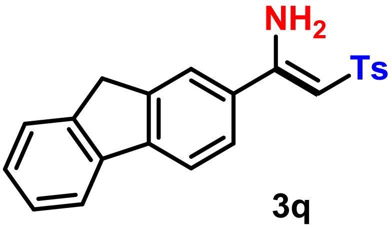
NH2
Ts

3q

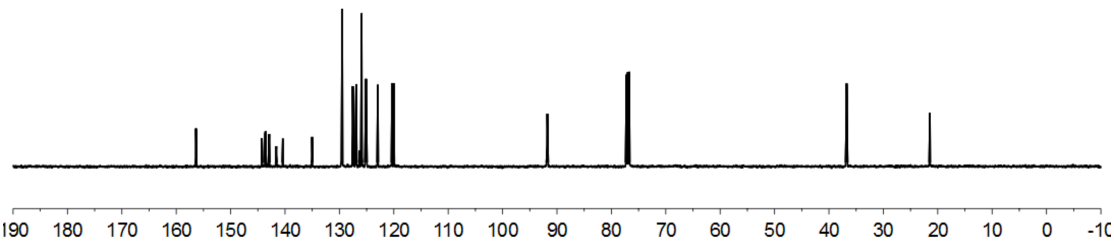
190 180 170 160 150 140 130 120 110 100 90 80 70 60 50 40 30 20 10 0 -10

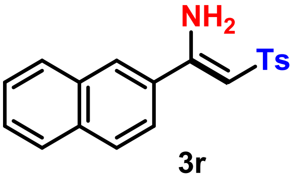
NH2
Ts

3r

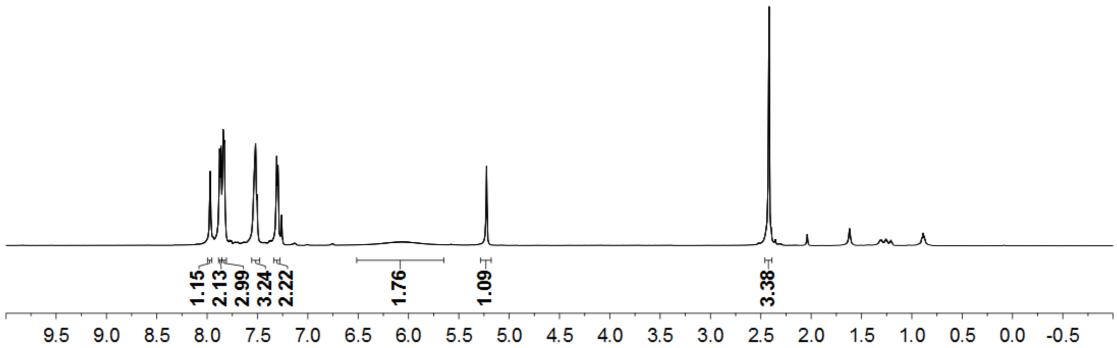
1.15
2.13
2.99
3.24
2.22
1.76
1.09
3.38
9.5 9.0 8.5 8.0 7.5 7.0 6.5 6.0 5.5 5.0 4.5 4.0 3.5 3.0 2.5 2.0 1.5 1.0 0.5 0.0 -0.5

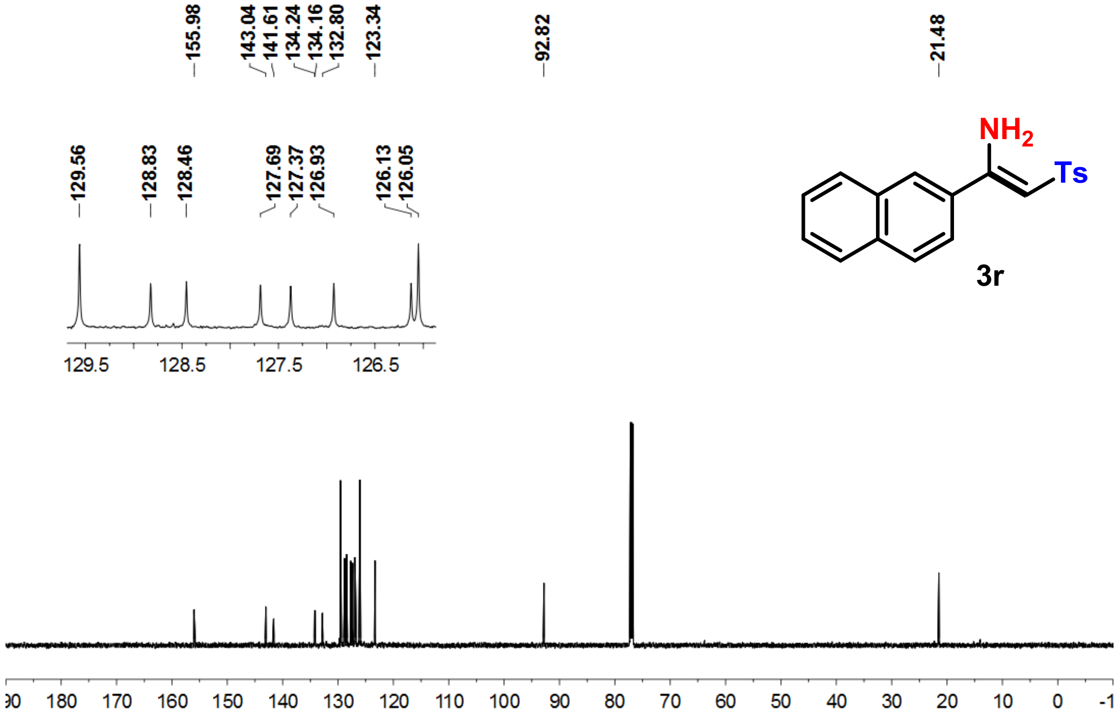
155.98
143.04
141.61
134.24
134.16
132.80
123.34
92.82
21.48
129.56
128.83
128.46
127.69
127.37
126.93
126.13
126.05
129.5 128.5 127.5 126.5
NH2
Ts
3r
180 170 160 150 140 130 120 110 100 90 80 70 60 50 40 30 20 10 0

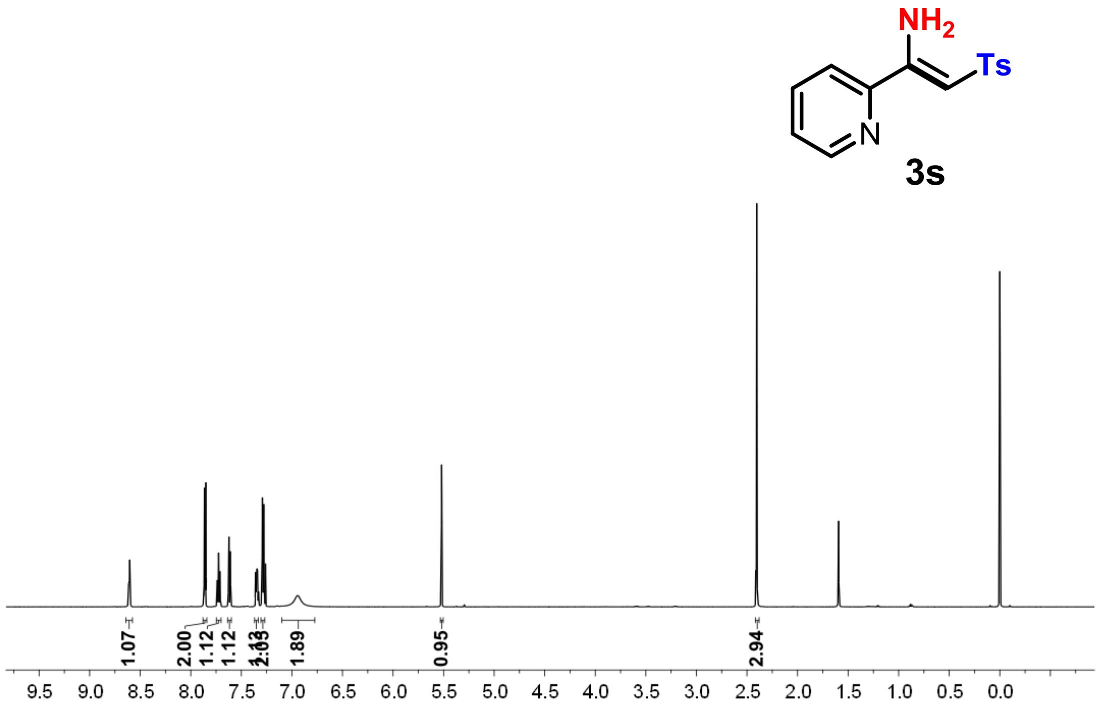
NH2
Ts
N
3s
1.07
2.00
1.12
1.12
1.89
0.95
2.94
9.5
9.0
8.5
8.0
7.5
7.0
6.5
6.0
5.5
5.0
4.5
4.0
3.5
3.0
2.5
2.0
1.5
1.0
0.5
0.0

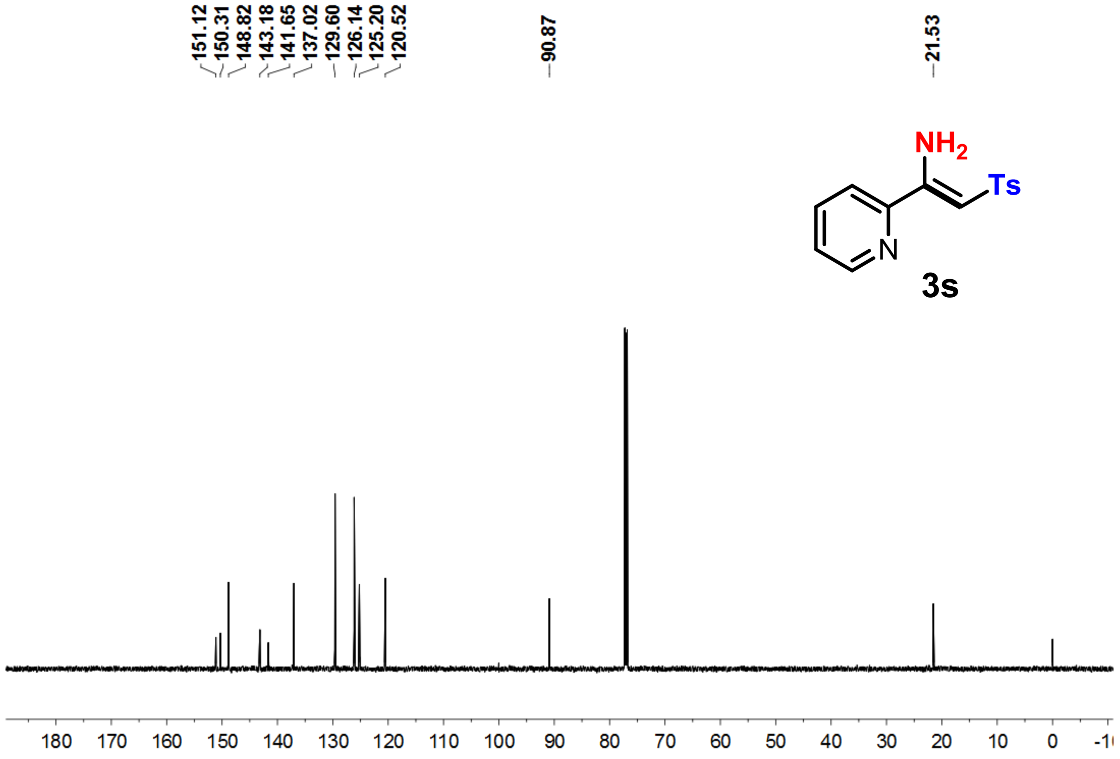
151.12
150.31
148.82
143.18
141.65
137.02
129.60
126.14
125.20
120.52
90.87
21.53
NH2
Ts
N
3s
180
170
160
150
140
130
120
110
100
90
80
70
60
50
40
30
20
10
0

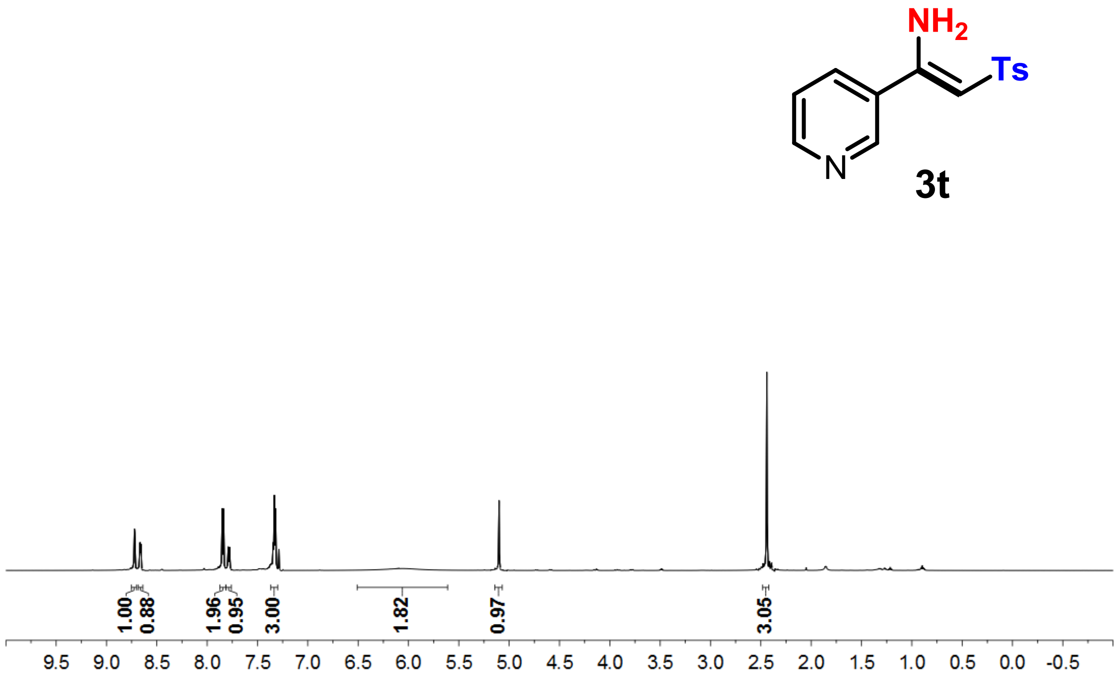
NH2
Ts
N
3t
1.00
0.88
1.96
0.95
3.00
1.82
0.97
3.05
9.5 9.0 8.5 8.0 7.5 7.0 6.5 6.0 5.5 5.0 4.5 4.0 3.5 3.0 2.5 2.0 1.5 1.0 0.5 0.0 -0.5

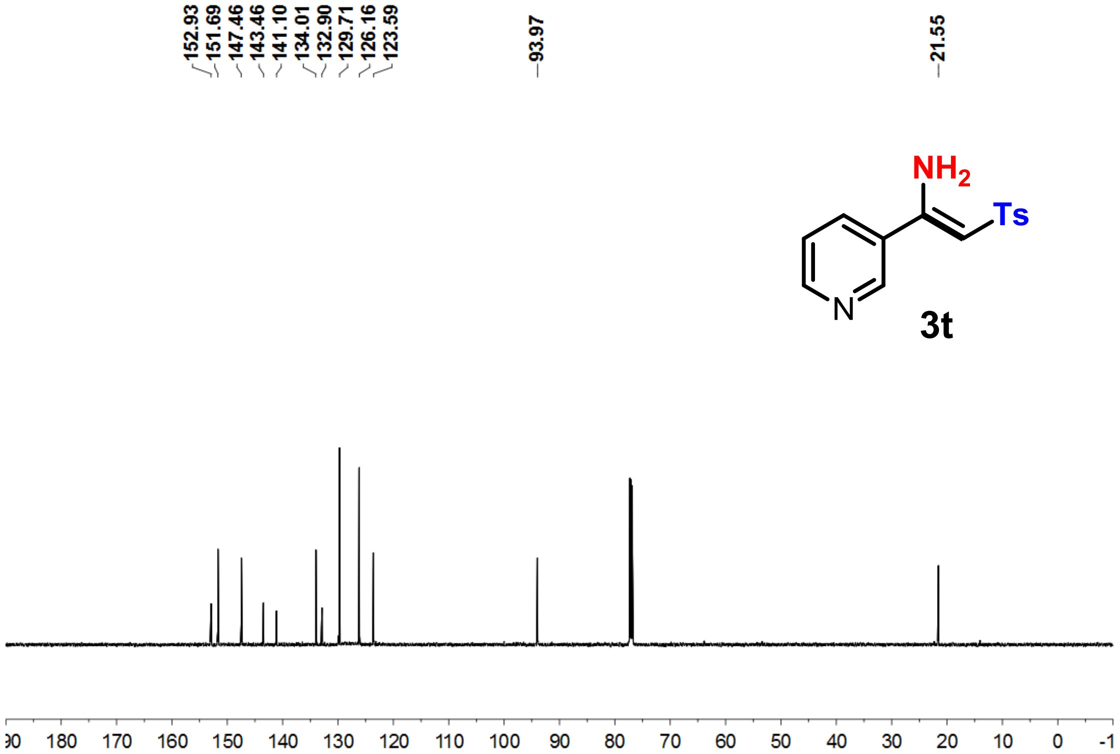
152.93
151.69
147.46
143.46
141.10
134.01
132.90
129.71
126.16
123.59
93.97
21.55
NH2
Ts
N
3t
180 170 160 150 140 130 120 110 100 90 80 70 60 50 40 30 20 10 0

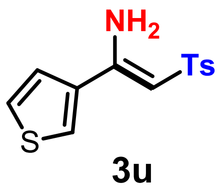
NH2
Ts
S

3u

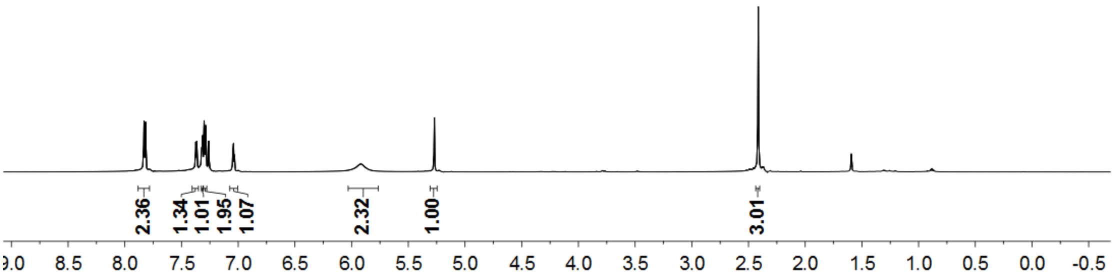
2.36
1.34
1.01
1.95
1.07
2.32
1.00
3.01
9.0 8.5 8.0 7.5 7.0 6.5 6.0 5.5 5.0 4.5 4.0 3.5 3.0 2.5 2.0 1.5 1.0 0.5 0.0 -0.5

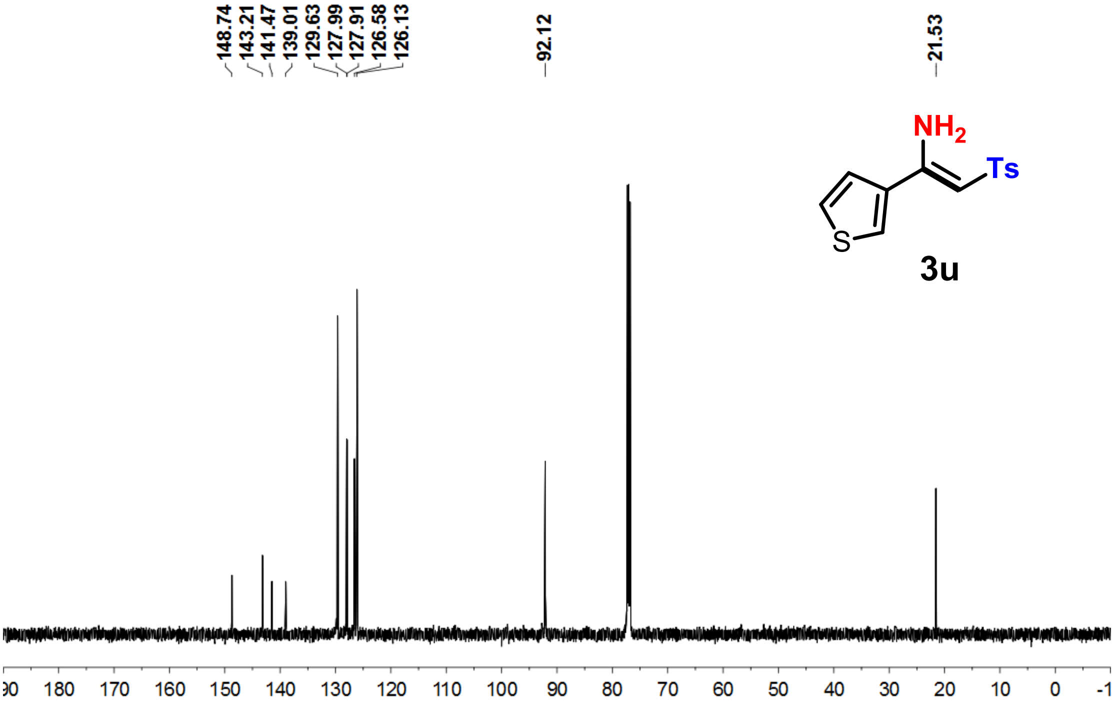
148.74
143.21
141.47
139.01
129.63
127.99
127.91
126.58
126.13
92.12
21.53
NH2
Ts
S
3u
190 180 170 160 150 140 130 120 110 100 90 80 70 60 50 40 30 20 10 0 -1

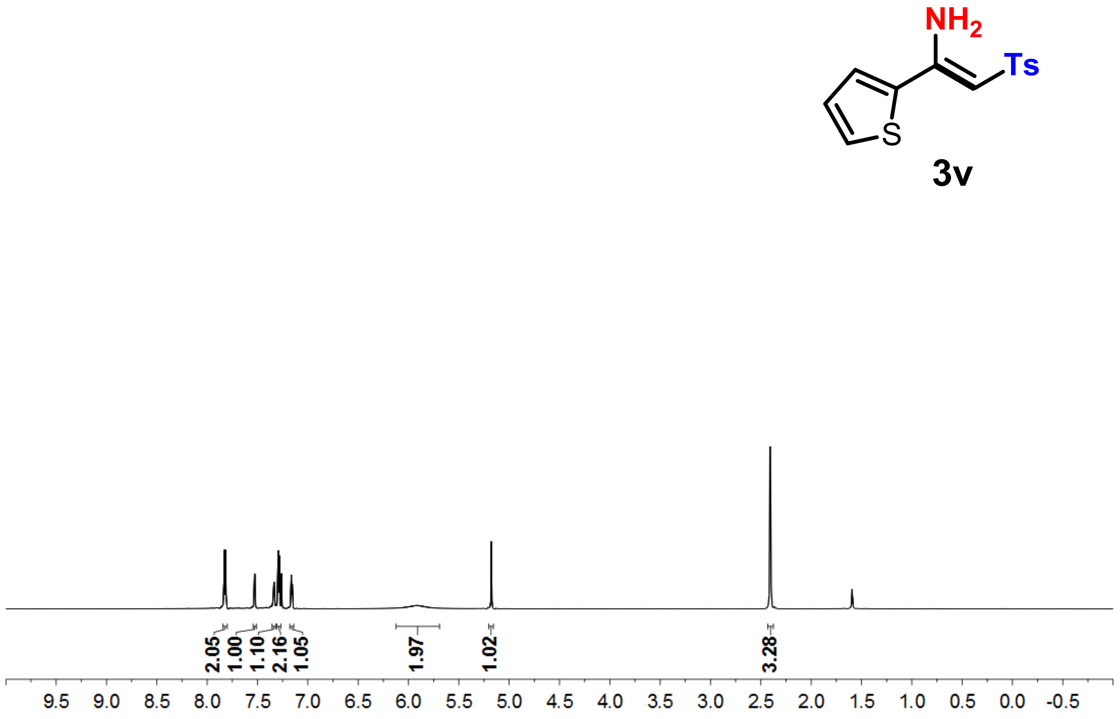
NH2
Ts
S
3v
2.05
1.00
1.10
2.16
1.05
1.97
1.02
3.28

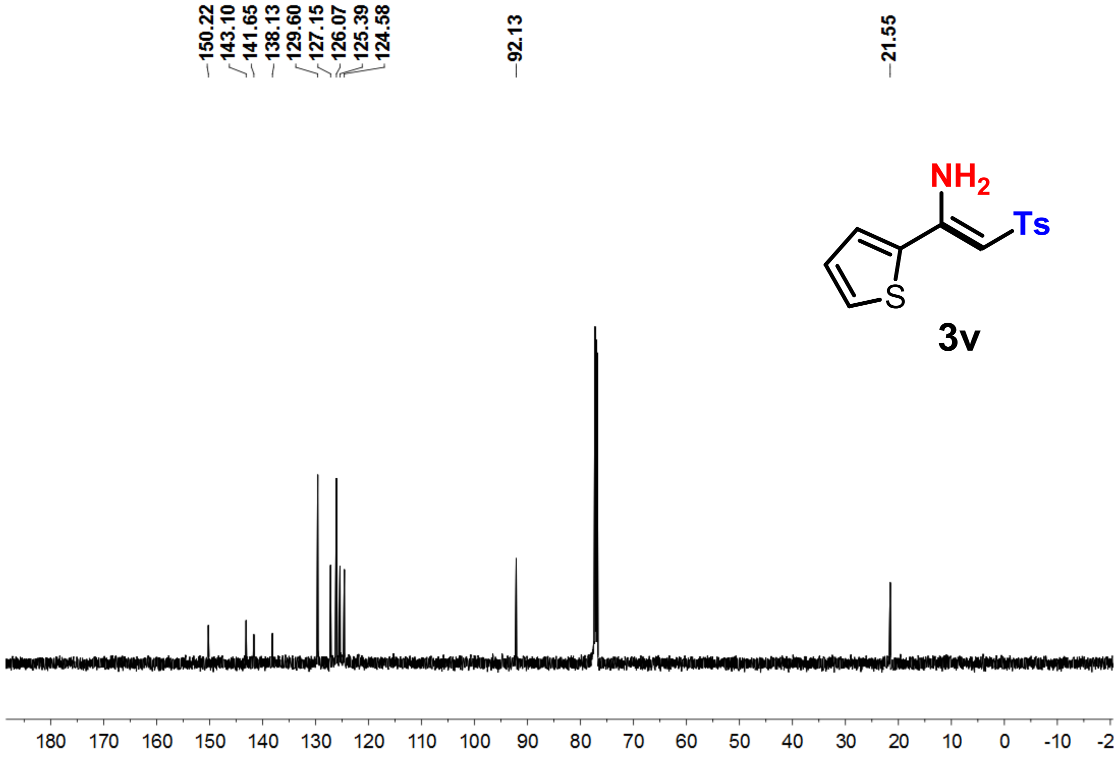
150.22
143.10
141.65
138.13
129.60
127.15
126.07
125.39
124.58
92.13
21.55
NH2
Ts
S
3v

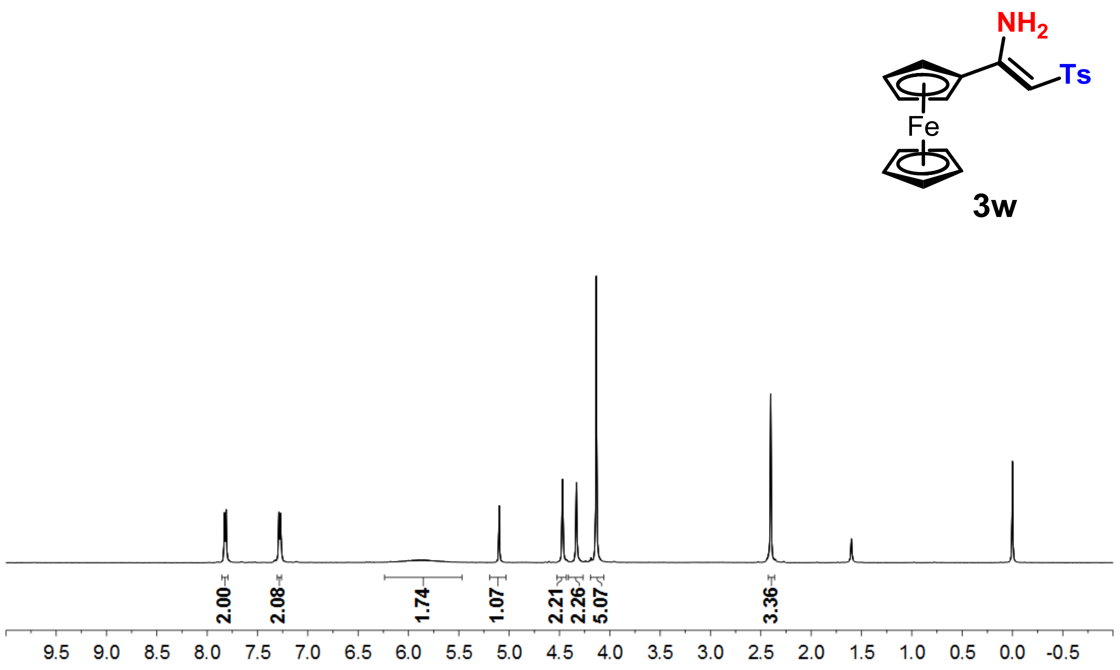
NH2
Ts
Fe
3w
2.00
2.08
1.74
1.07
2.21
2.26
5.07
3.36
9.5 9.0 8.5 8.0 7.5 7.0 6.5 6.0 5.5 5.0 4.5 4.0 3.5 3.0 2.5 2.0 1.5 1.0 0.5 0.0 -0.5

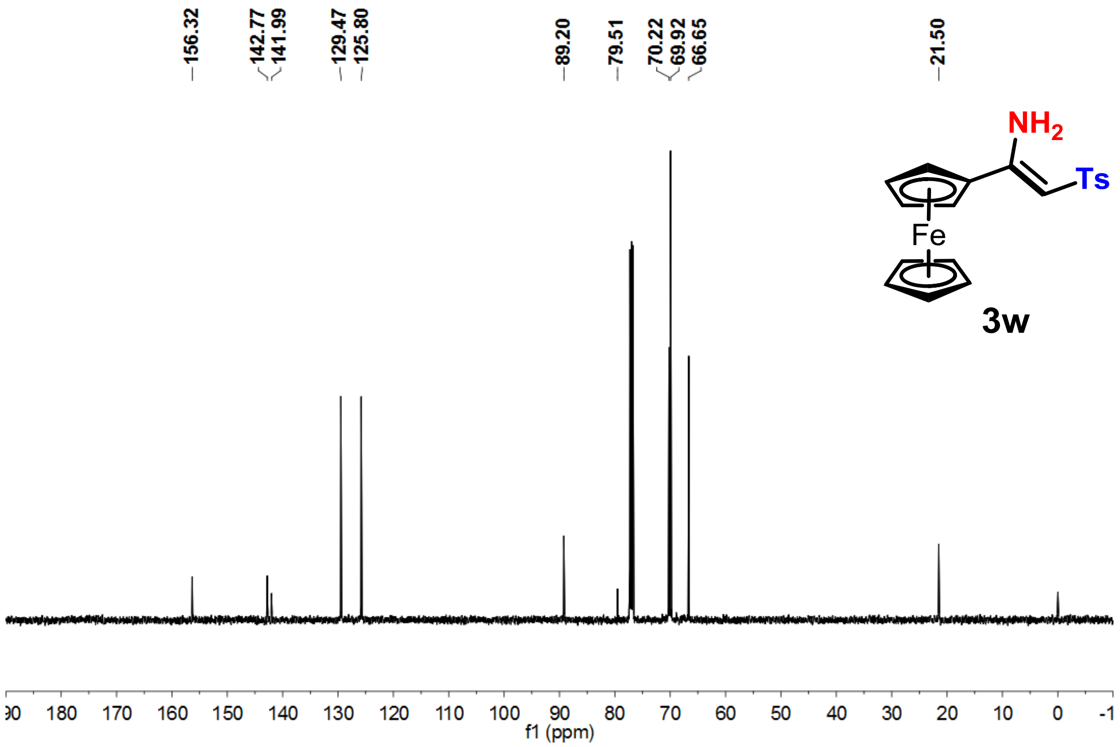
156.32
142.77
141.99
129.47
125.80
89.20
79.51
70.22
69.92
66.65
21.50
NH2
Ts
Fe
3w
180 170 160 150 140 130 120 110 100 90 80 70 60 50 40 30 20 10 0
f1 (ppm)

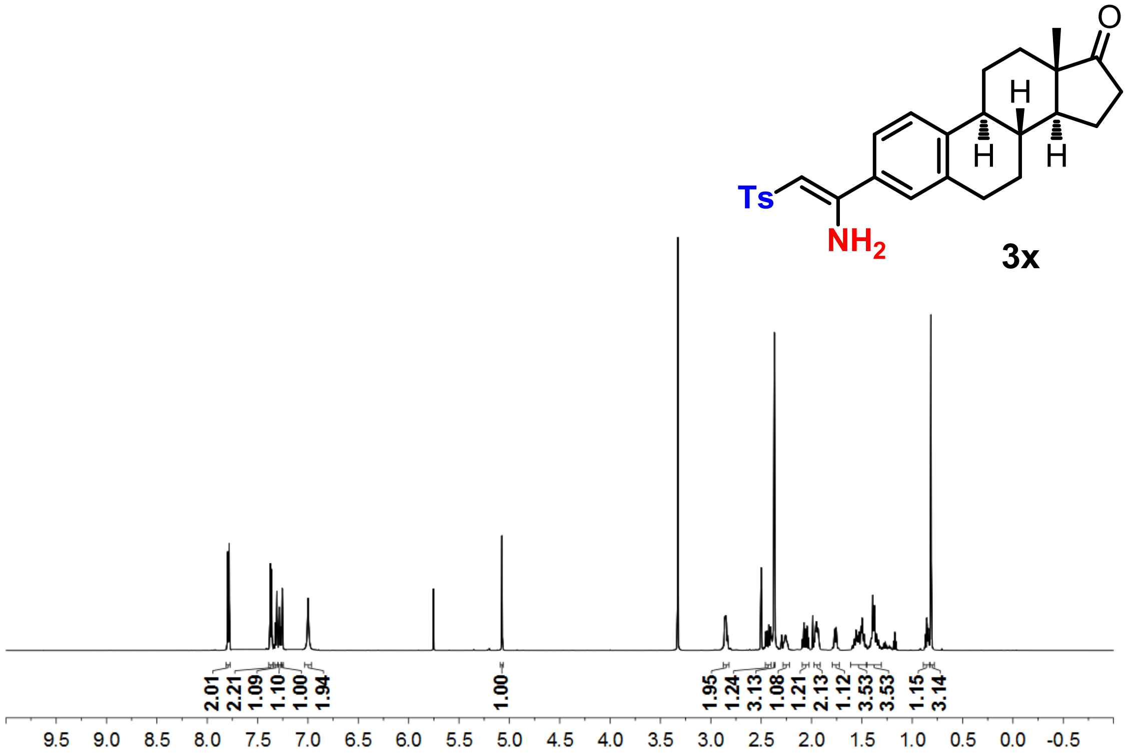
Ts
NH2
3x
2.01
2.21
1.09
1.10
1.00
1.94
1.00
1.95
1.24
3.13
1.08
1.21
2.13
1.12
3.53
3.53
1.15
3.14
9.5 9.0 8.5 8.0 7.5 7.0 6.5 6.0 5.5 5.0 4.5 4.0 3.5 3.0 2.5 2.0 1.5 1.0 0.5 0.0 -0.5

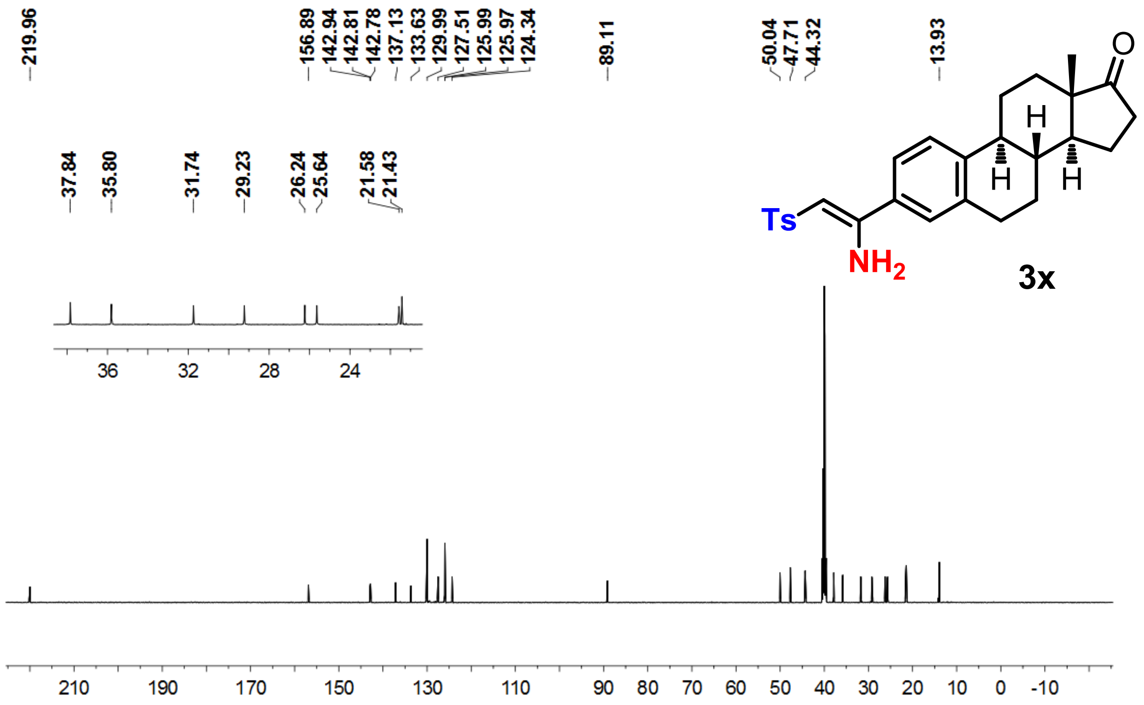
219.96
156.89
142.94
142.81
142.78
137.13
133.63
129.99
127.51
125.99
125.97
124.34
89.11
50.04
47.71
44.32
13.93
37.84
35.80
31.74
29.23
26.24
25.64
21.58
21.43
36 32 28 24
Ts
NH2
3x
210 190 170 150 130 110 90 80 70 60 50 40 30 20 10 0 -10

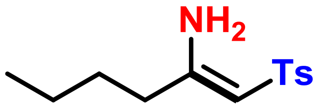

NH2
Ts

4a

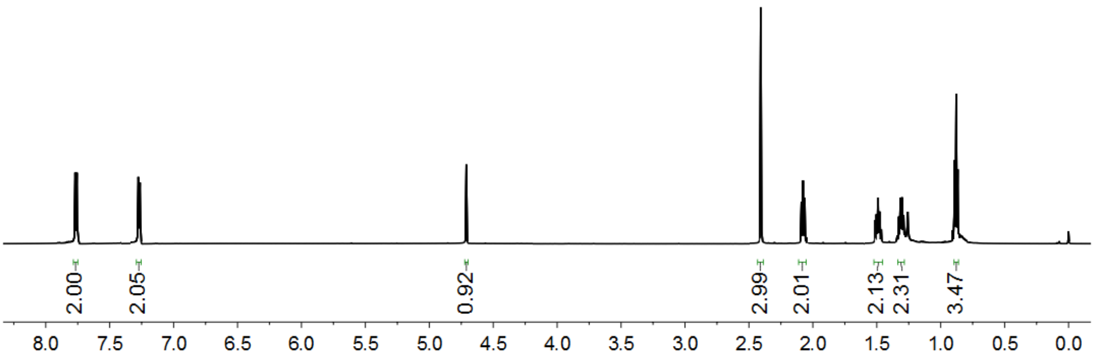

2.00
2.05
0.92
2.99
2.01
2.13
2.31
3.47
8.0 7.5 7.0 6.5 6.0 5.5 5.0 4.5 4.0 3.5 3.0 2.5 2.0 1.5 1.0 0.5 0.0

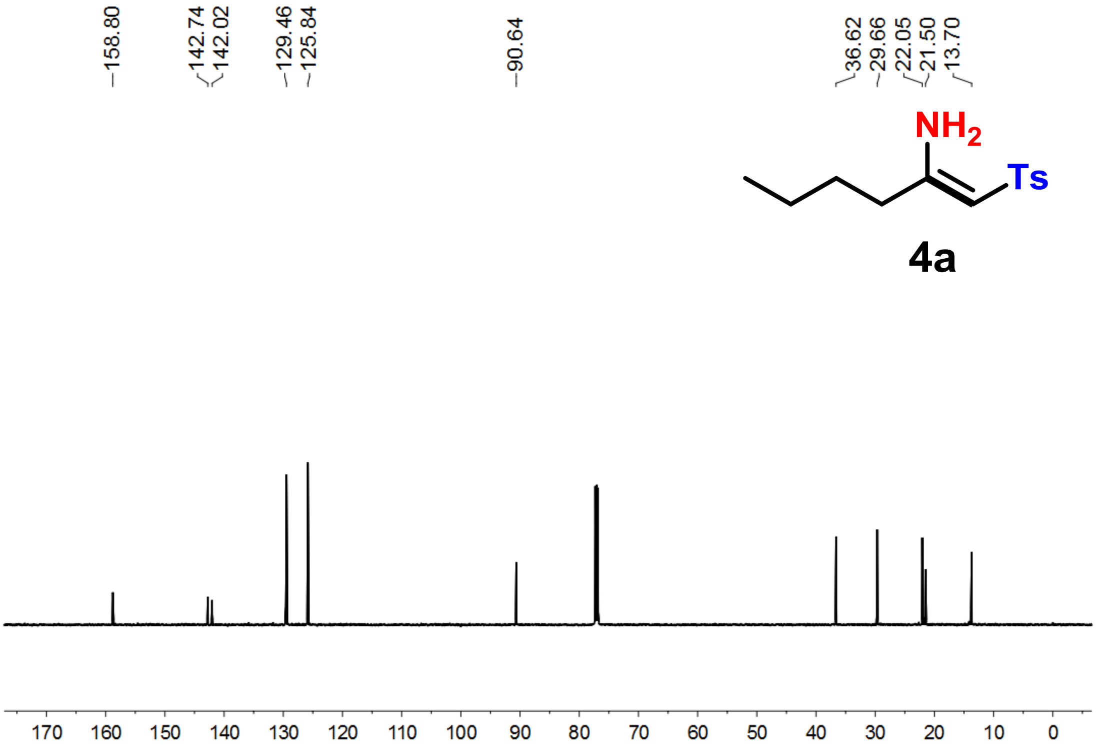

158.80
142.74
142.02
129.46
125.84
90.64
36.62
29.66
22.05
21.50
13.70
NH2
Ts
4a
170 160 150 140 130 120 110 100 90 80 70 60 50 40 30 20 10 0

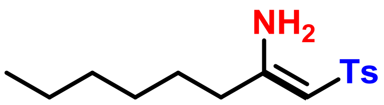
NH2
Ts

4b

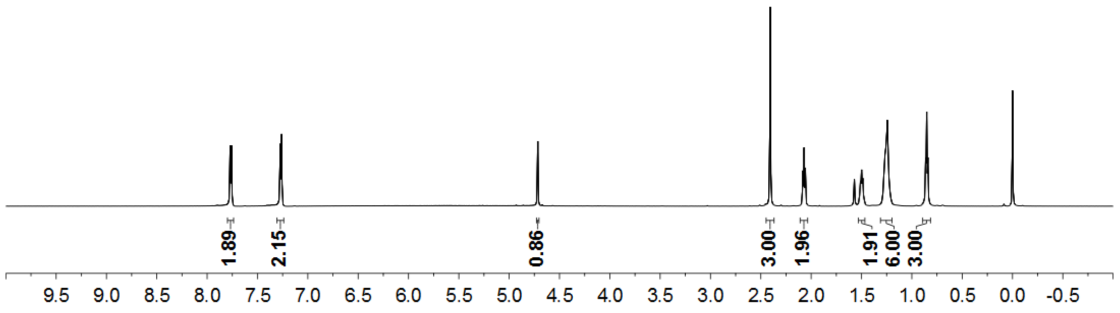
1.89
2.15
0.86
3.00
1.96
1.91
6.00
3.00
9.5 9.0 8.5 8.0 7.5 7.0 6.5 6.0 5.5 5.0 4.5 4.0 3.5 3.0 2.5 2.0 1.5 1.0 0.5 0.0 -0.5

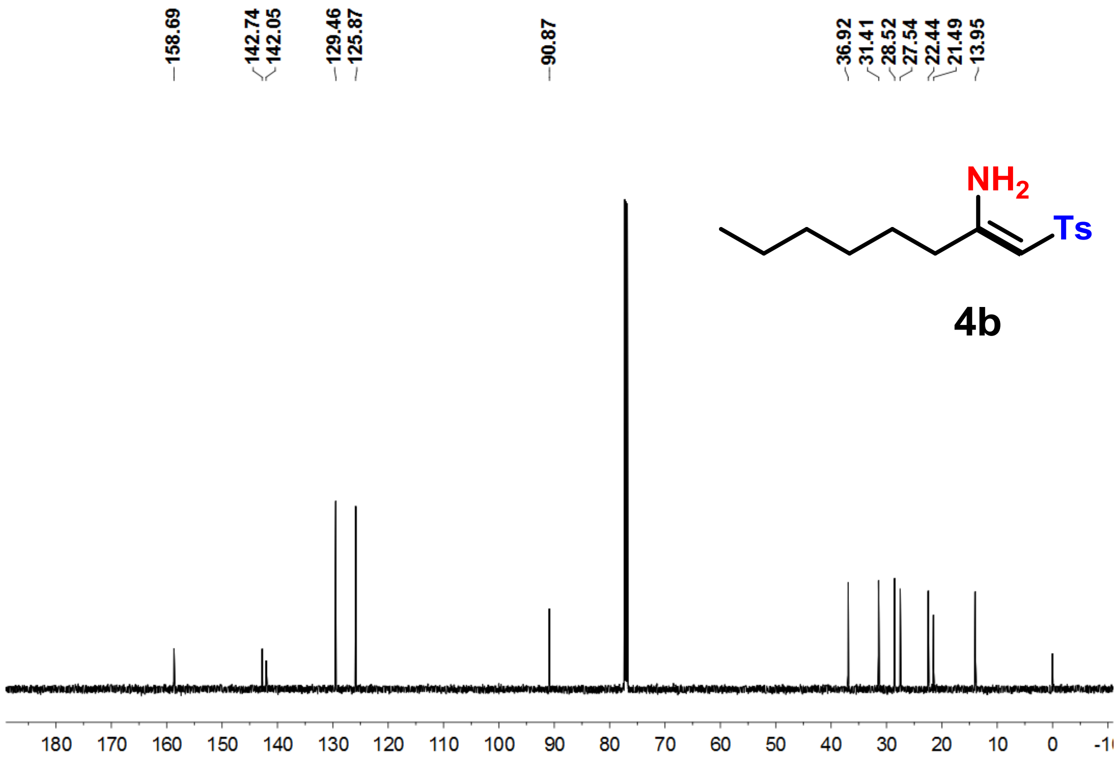
158.69
142.74
142.05
129.46
125.87
90.87
36.92
31.41
28.52
27.54
22.44
21.49
13.95
NH2
Ts
4b
180 170 160 150 140 130 120 110 100 90 80 70 60 50 40 30 20 10 0

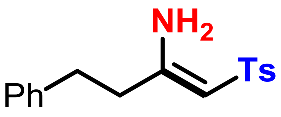
NH2
Ts
Ph

4c

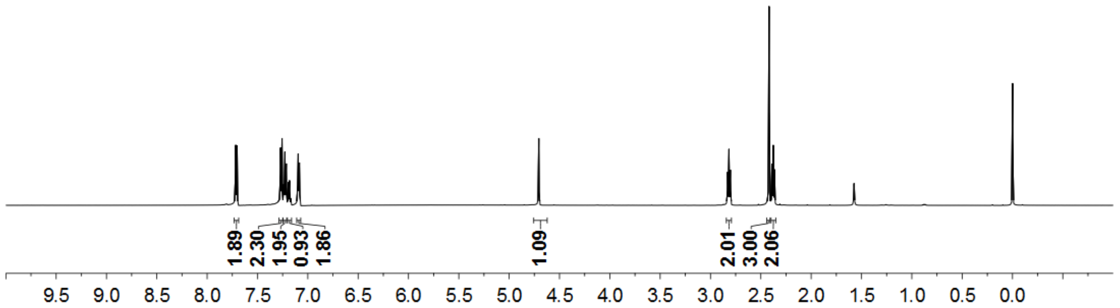
1.89
2.30
1.95
0.93
1.86
1.09
2.01
3.00
2.06
9.5 9.0 8.5 8.0 7.5 7.0 6.5 6.0 5.5 5.0 4.5 4.0 3.5 3.0 2.5 2.0 1.5 1.0 0.5 0.0

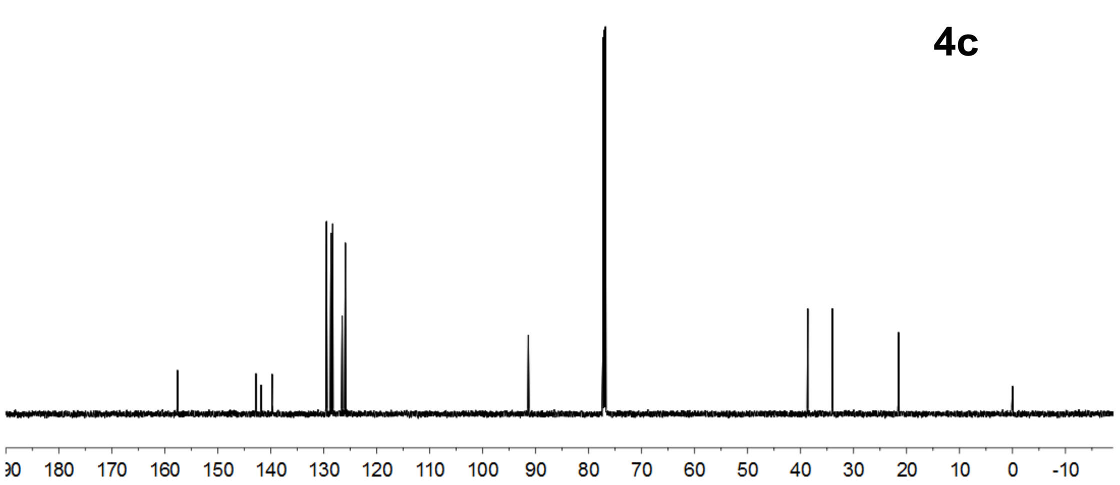
157.60
142.80
141.82
139.74
129.47
128.63
128.29
126.53
125.92
91.38
38.65
33.98
21.52
180 170 160 150 140 130 120 110 100 90 80 70 60 50 40 30 20 10 0 -10

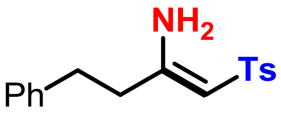
NH2
Ts
Ph

4c

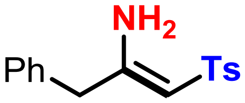

NH2
Ph
Ts

4d

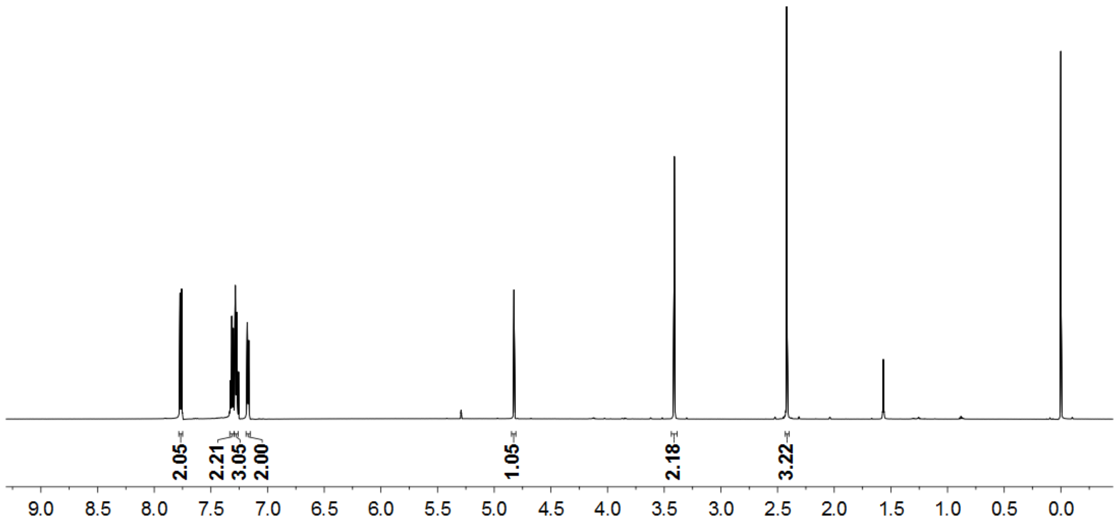

2.05
2.21
3.05
2.00
1.05
2.18
3.22
9.0 8.5 8.0 7.5 7.0 6.5 6.0 5.5 5.0 4.5 4.0 3.5 3.0 2.5 2.0 1.5 1.0 0.5 0.0

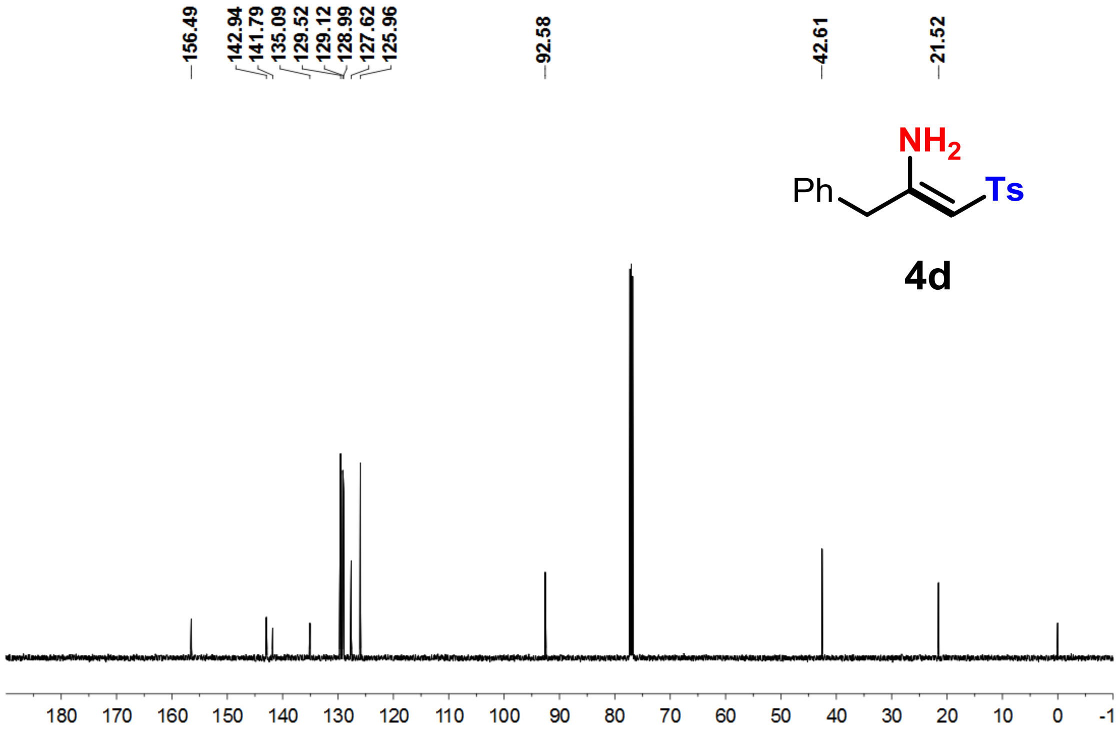

156.49
142.94
141.79
135.09
129.52
129.12
128.99
127.62
125.96
92.58
42.61
21.52
180 170 160 150 140 130 120 110 100 90 80 70 60 50 40 30 20 10 0 -1

4d

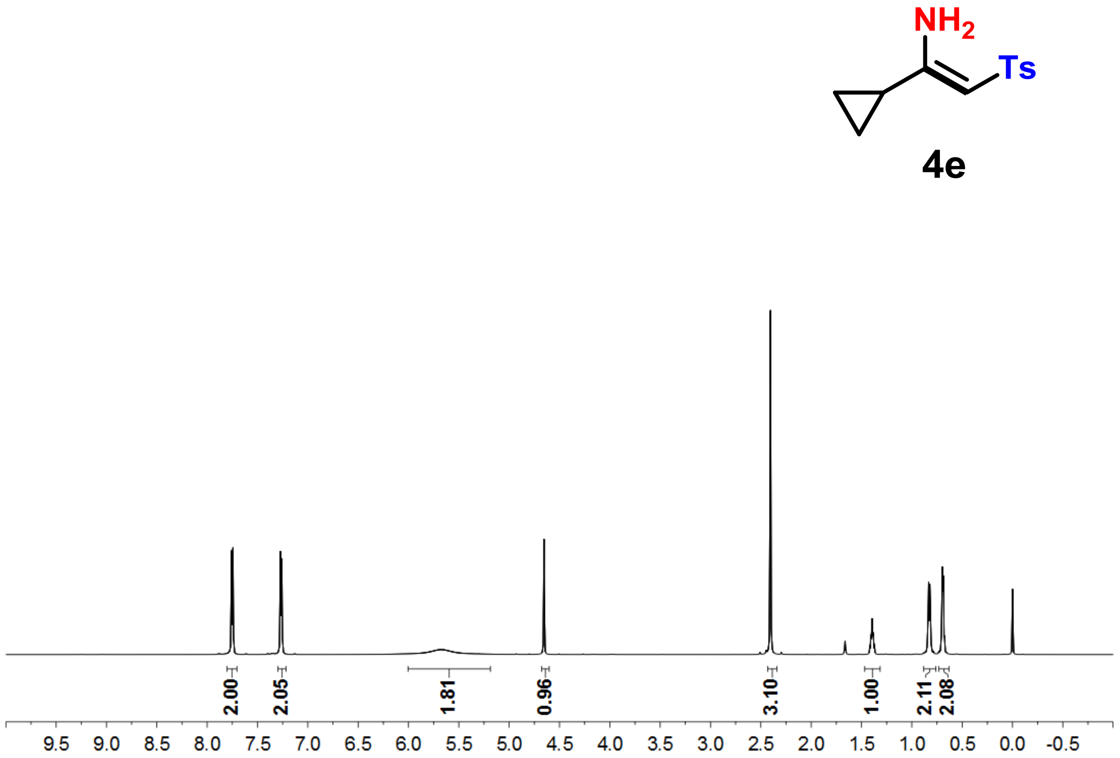
NH2
Ts
4e
2.00
2.05
1.81
0.96
3.10
1.00
2.11
2.08
9.5 9.0 8.5 8.0 7.5 7.0 6.5 6.0 5.5 5.0 4.5 4.0 3.5 3.0 2.5 2.0 1.5 1.0 0.5 0.0 -0.5

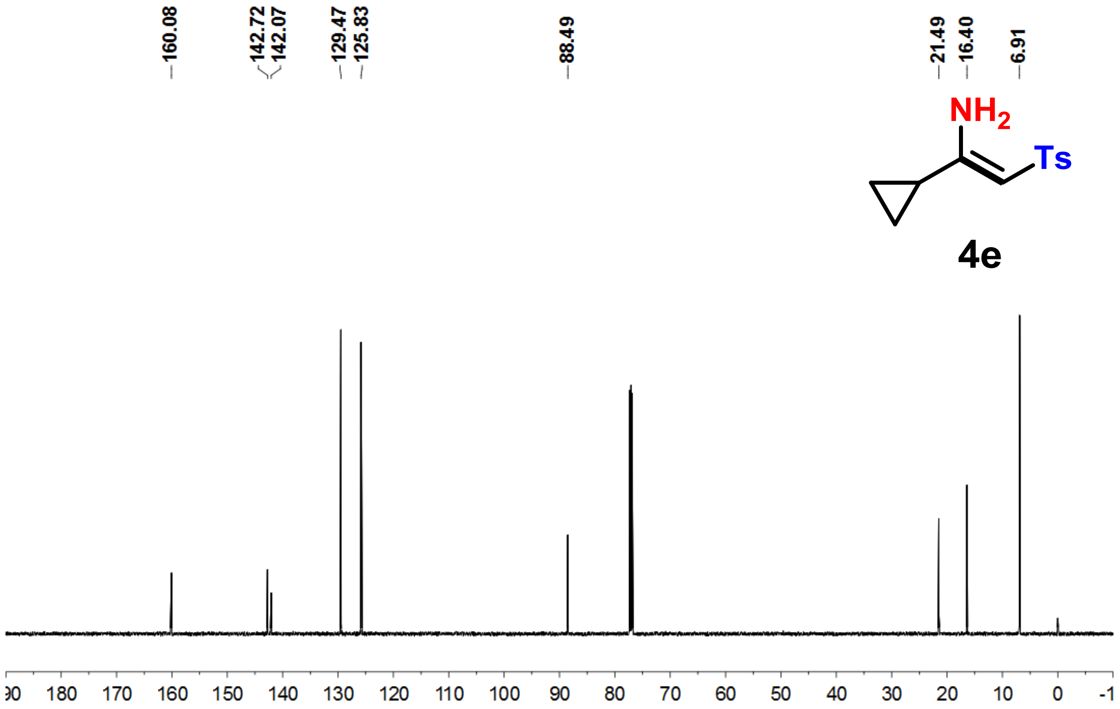
160.08
142.72
142.07
129.47
125.83
88.49
21.49
16.40
6.91
NH2
Ts
4e
180 170 160 150 140 130 120 110 100 90 80 70 60 50 40 30 20 10 0

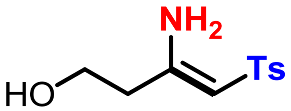
NH2
Ts
HO

4f

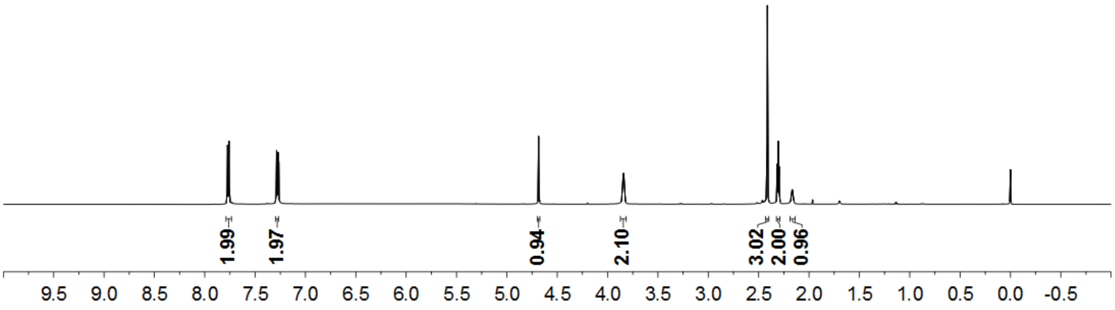
1.99
1.97
0.94
2.10
3.02
2.00
0.96
9.5 9.0 8.5 8.0 7.5 7.0 6.5 6.0 5.5 5.0 4.5 4.0 3.5 3.0 2.5 2.0 1.5 1.0 0.5 0.0 -0.5

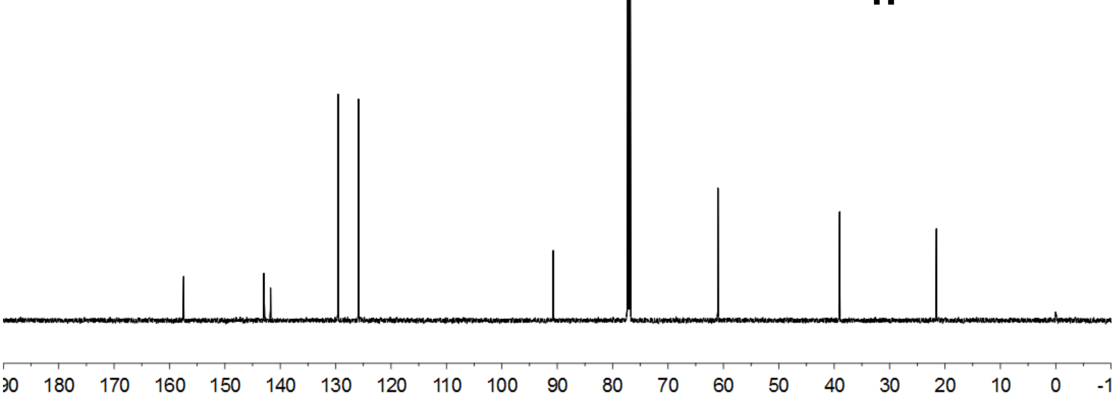
157.44
142.96
141.76
129.56
125.87
90.72
61.00
39.00
21.53
180 170 160 150 140 130 120 110 100 90 80 70 60 50 40 30 20 10 0

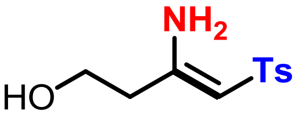
NH2
Ts
HO

4f

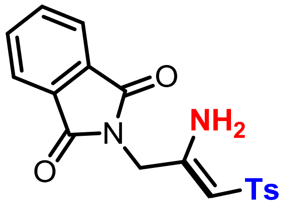
O
O
N
NH2
Ts

4g

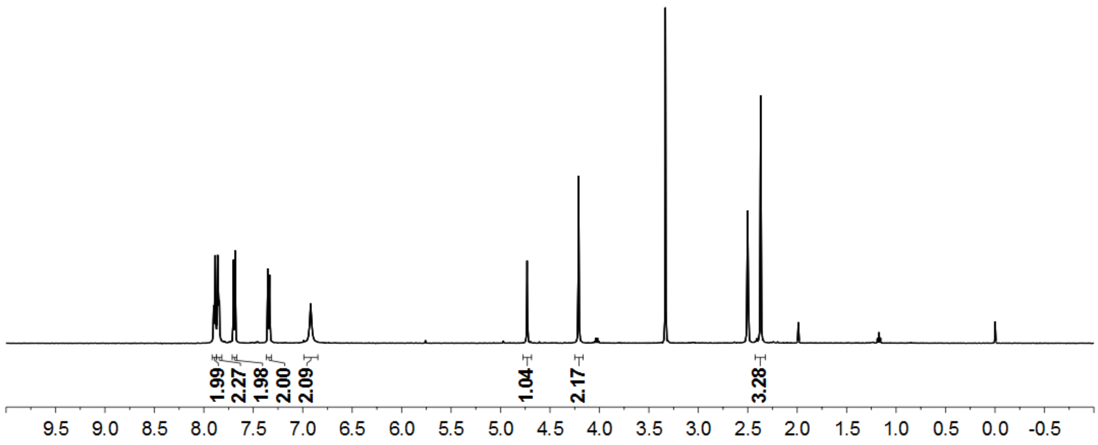
1.99
2.27
1.98
2.00
2.09
1.04
2.17
3.28
9.5 9.0 8.5 8.0 7.5 7.0 6.5 6.0 5.5 5.0 4.5 4.0 3.5 3.0 2.5 2.0 1.5 1.0 0.5 0.0 -0.5

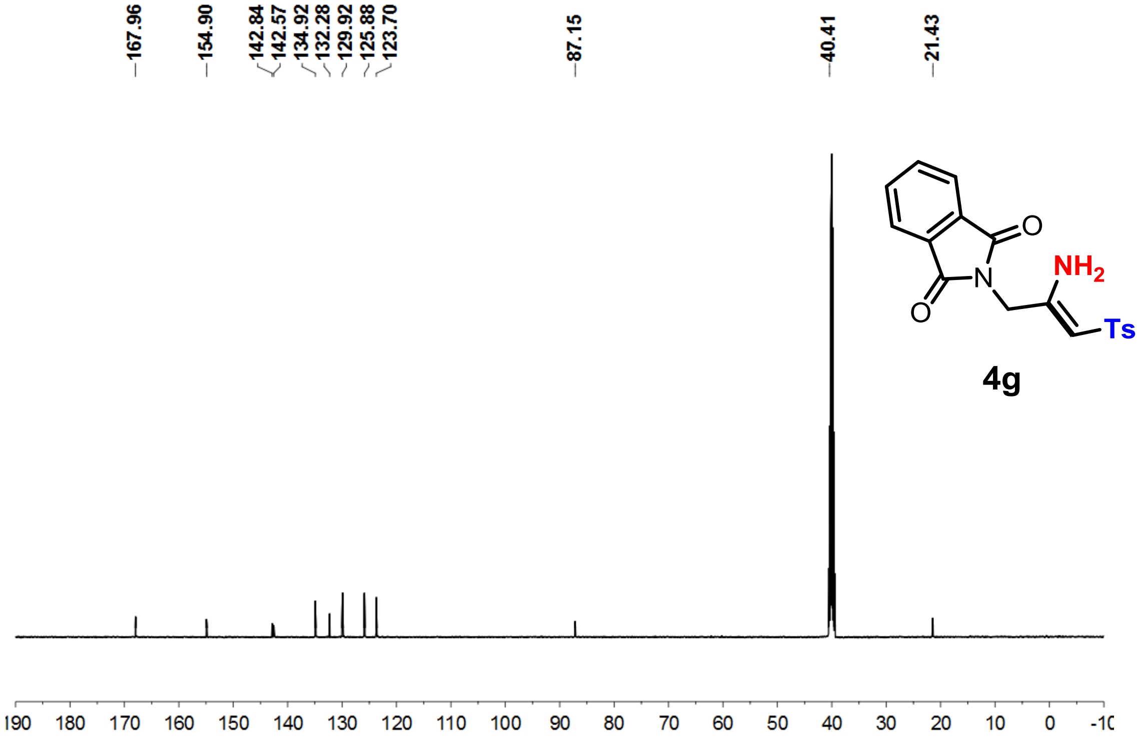
167.96
154.90
142.84
142.57
134.92
132.28
129.92
125.88
123.70
87.15
40.41
21.43
190 180 170 160 150 140 130 120 110 100 90 80 70 60 50 40 30 20 10 0 -10
O
O
N
NH2
Ts

4g

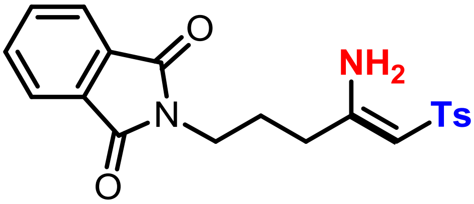
O
N
O
NH2
Ts

4h

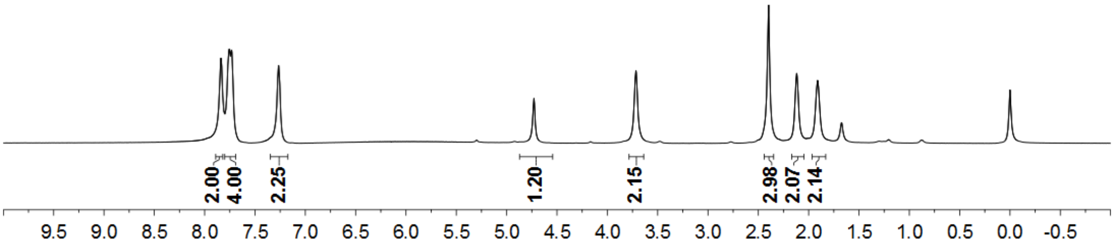
2.00
4.00
2.25
1.20
2.15
2.98
2.07
2.14
9.5 9.0 8.5 8.0 7.5 7.0 6.5 6.0 5.5 5.0 4.5 4.0 3.5 3.0 2.5 2.0 1.5 1.0 0.5 0.0 -0.5

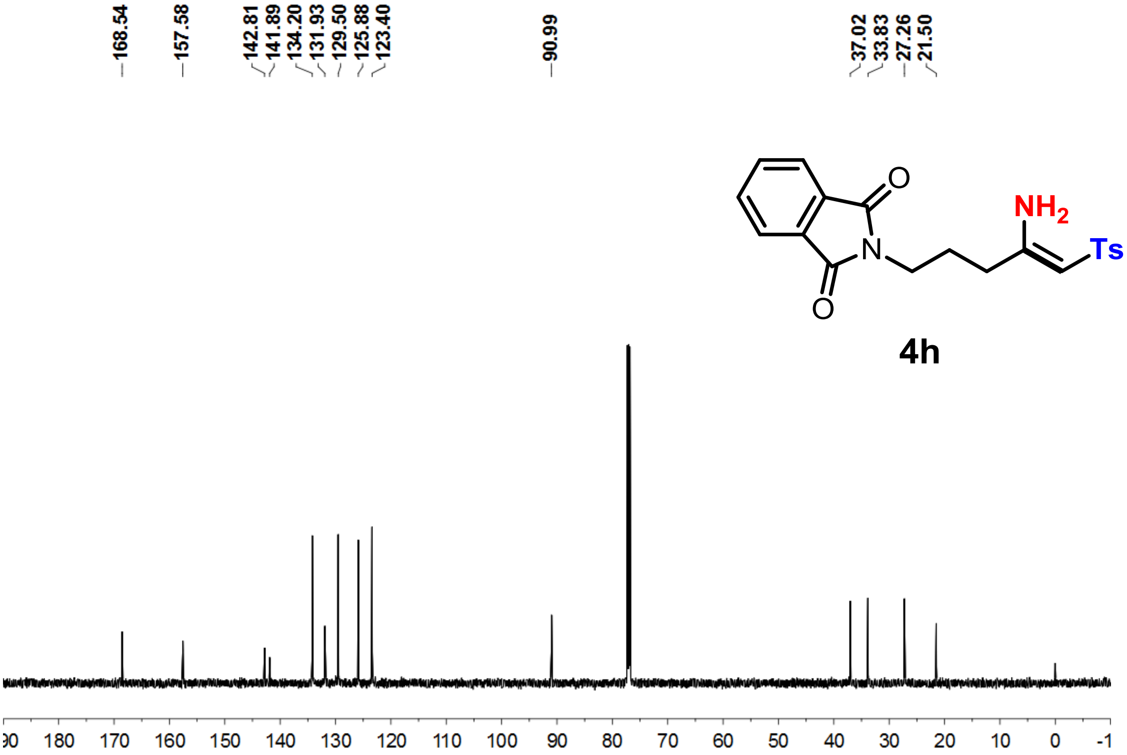
168.54
157.58
142.81
141.89
134.20
131.93
129.50
125.88
123.40
90.99
37.02
33.83
27.26
21.50
O
N
O
NH2
Ts
4h
180 170 160 150 140 130 120 110 100 90 80 70 60 50 40 30 20 10 0 -1

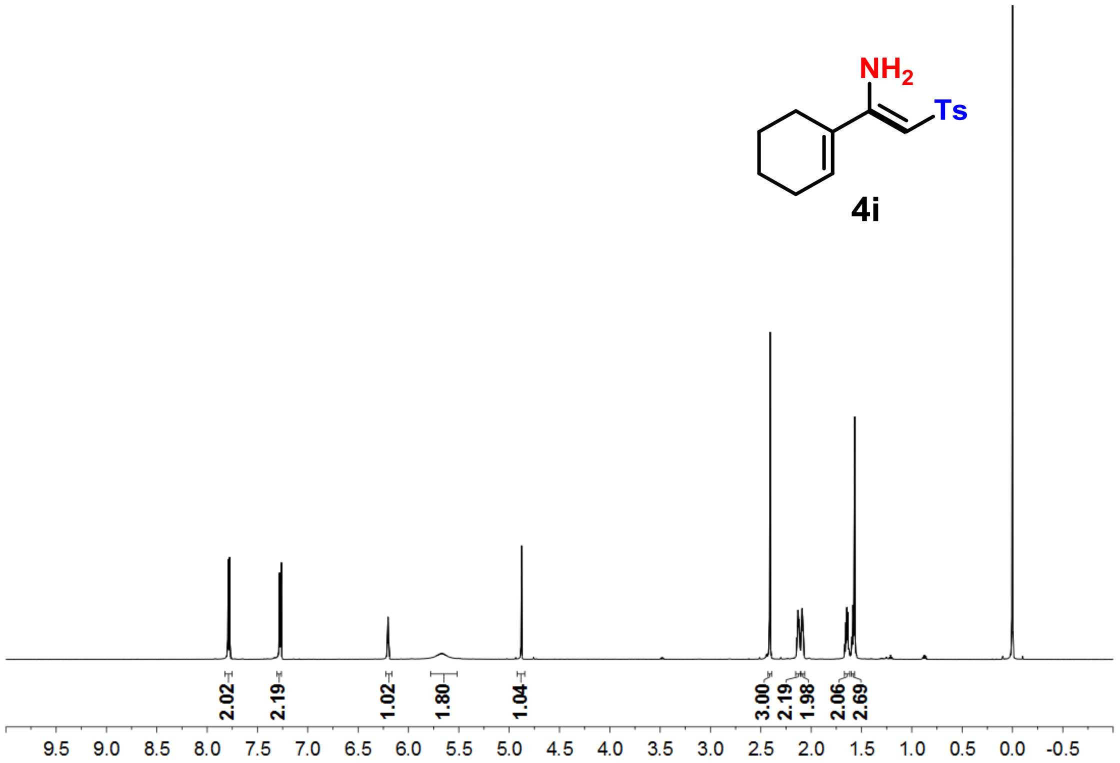
NH2
Ts
4i
2.02
2.19
1.02
1.80
1.04
3.00
2.19
1.98
2.06
2.69
9.5 9.0 8.5 8.0 7.5 7.0 6.5 6.0 5.5 5.0 4.5 4.0 3.5 3.0 2.5 2.0 1.5 1.0 0.5 0.0 -0.5

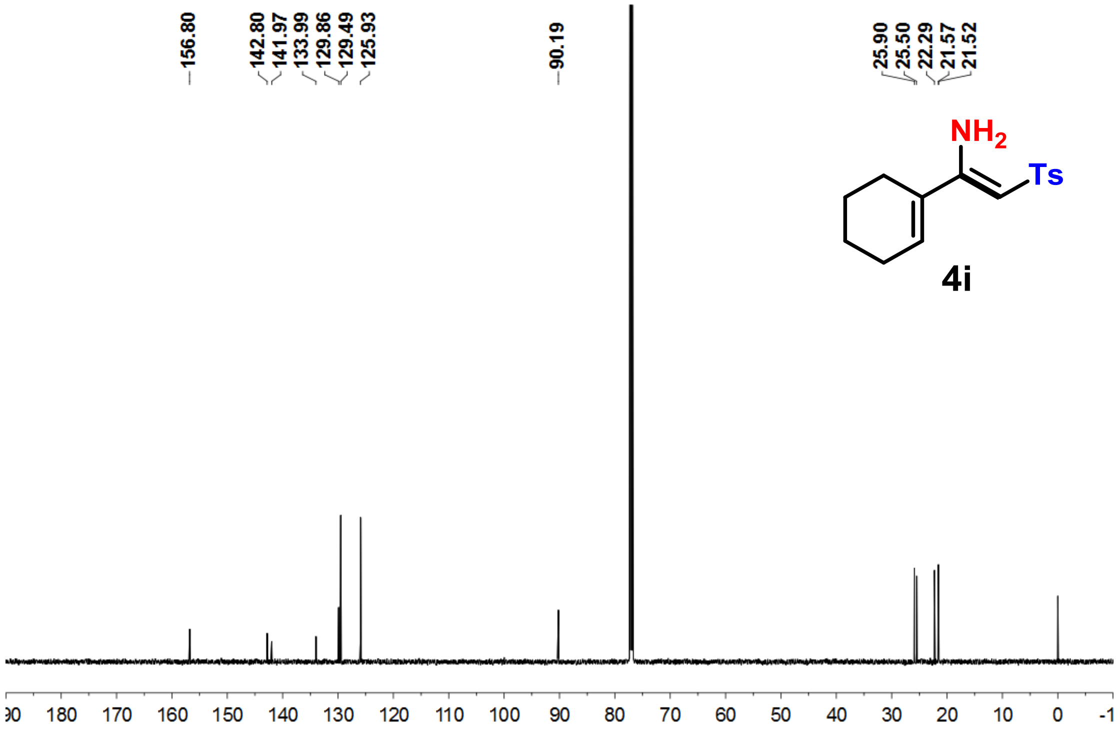
156.80
142.80
141.97
133.99
129.86
129.49
125.93
90.19
25.90
25.50
22.29
21.57
21.52
NH2
Ts
4i
180 170 160 150 140 130 120 110 100 90 80 70 60 50 40 30 20 10 0

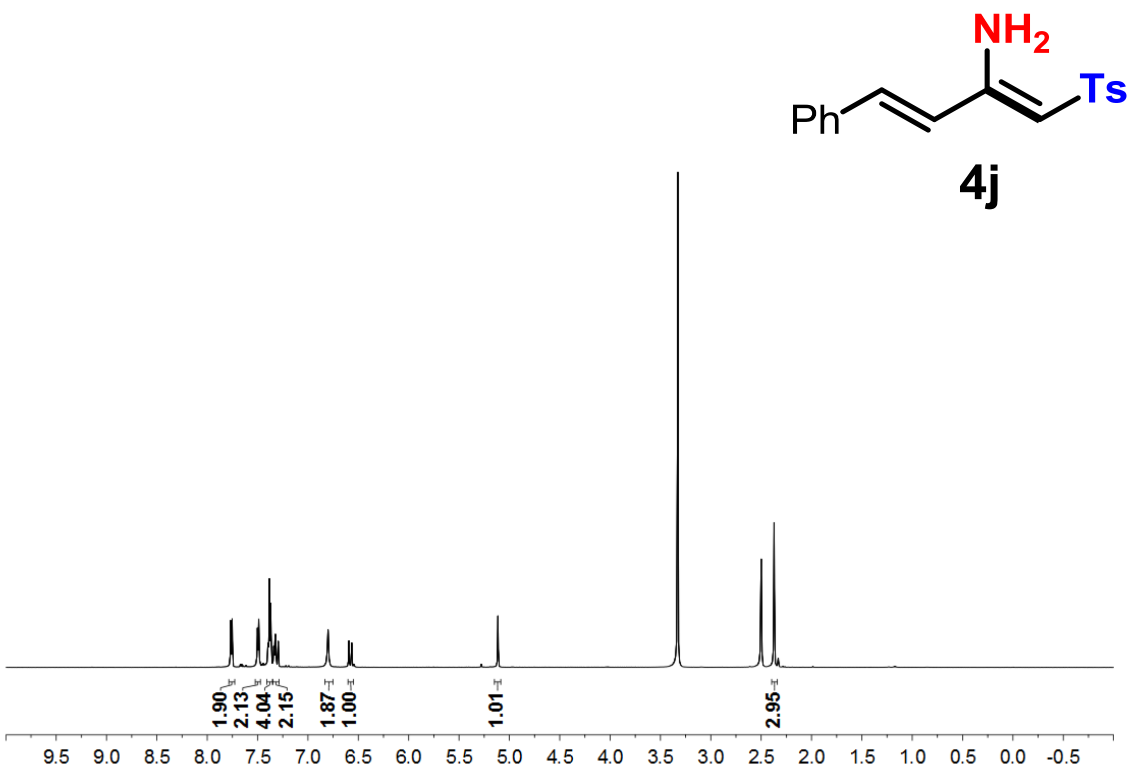
NH2
Ts
Ph
4j
1.90
2.13
4.04
2.15
1.87
1.00
1.01
2.95
9.5
9.0
8.5
8.0
7.5
7.0
6.5
6.0
5.5
5.0
4.5
4.0
3.5
3.0
2.5
2.0
1.5
1.0
0.5
0.0
-0.5

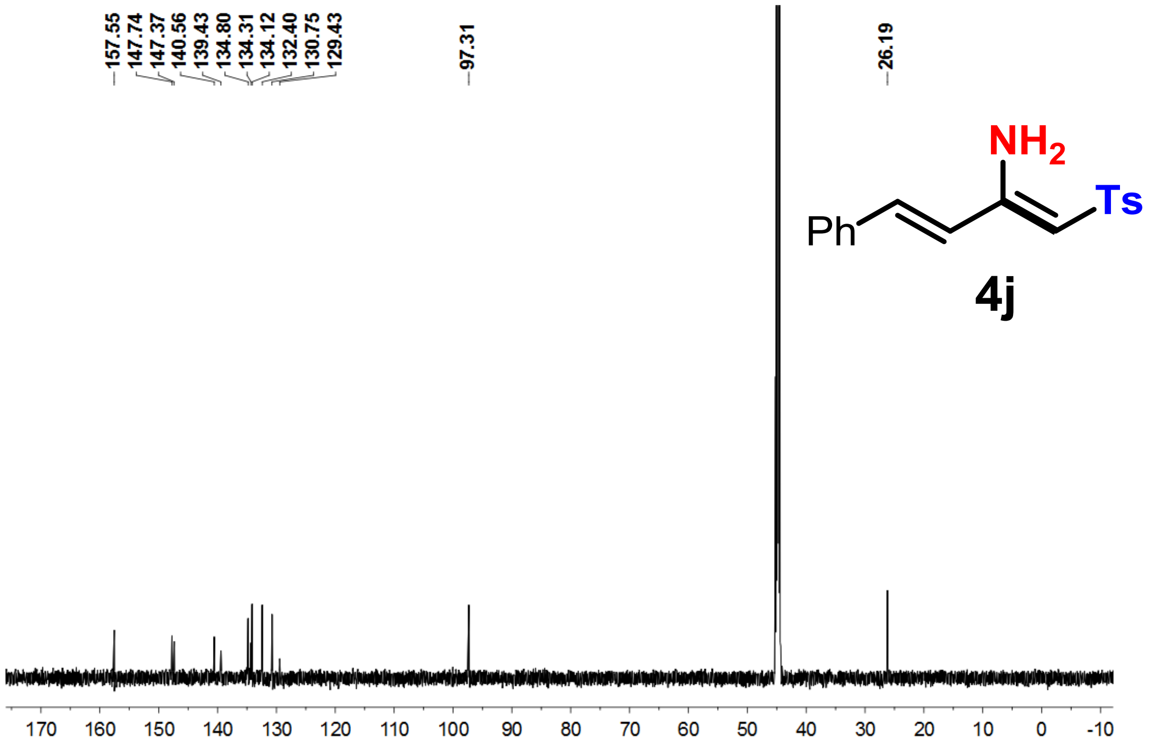
157.55
147.74
147.37
140.56
139.43
134.80
134.31
134.12
132.40
130.75
129.43
97.31
26.19
NH2
Ts
Ph
4j
170
160
150
140
130
120
110
100
90
80
70
60
50
40
30
20
10
0
-10

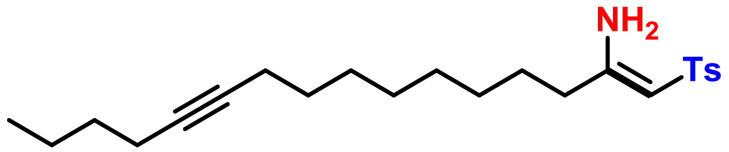
NH2
Ts

4k

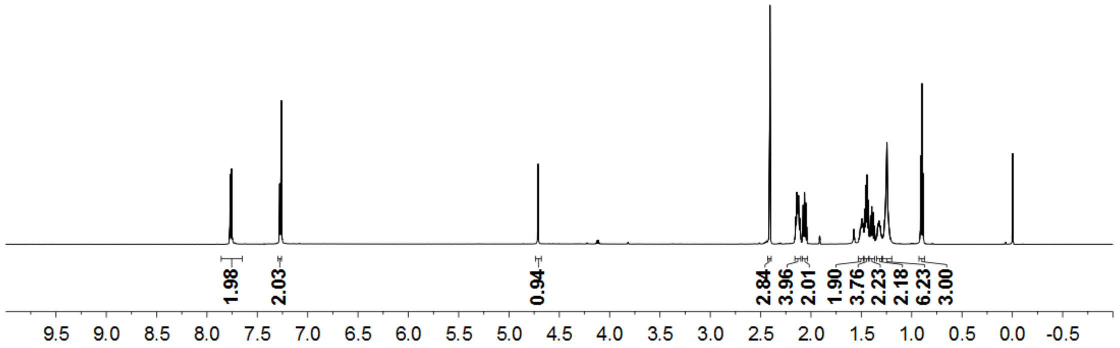
1.98
2.03
0.94
2.84
3.96
2.01
1.90
3.76
2.23
2.18
6.23
3.00
9.5 9.0 8.5 8.0 7.5 7.0 6.5 6.0 5.5 5.0 4.5 4.0 3.5 3.0 2.5 2.0 1.5 1.0 0.5 0.0 -0.5

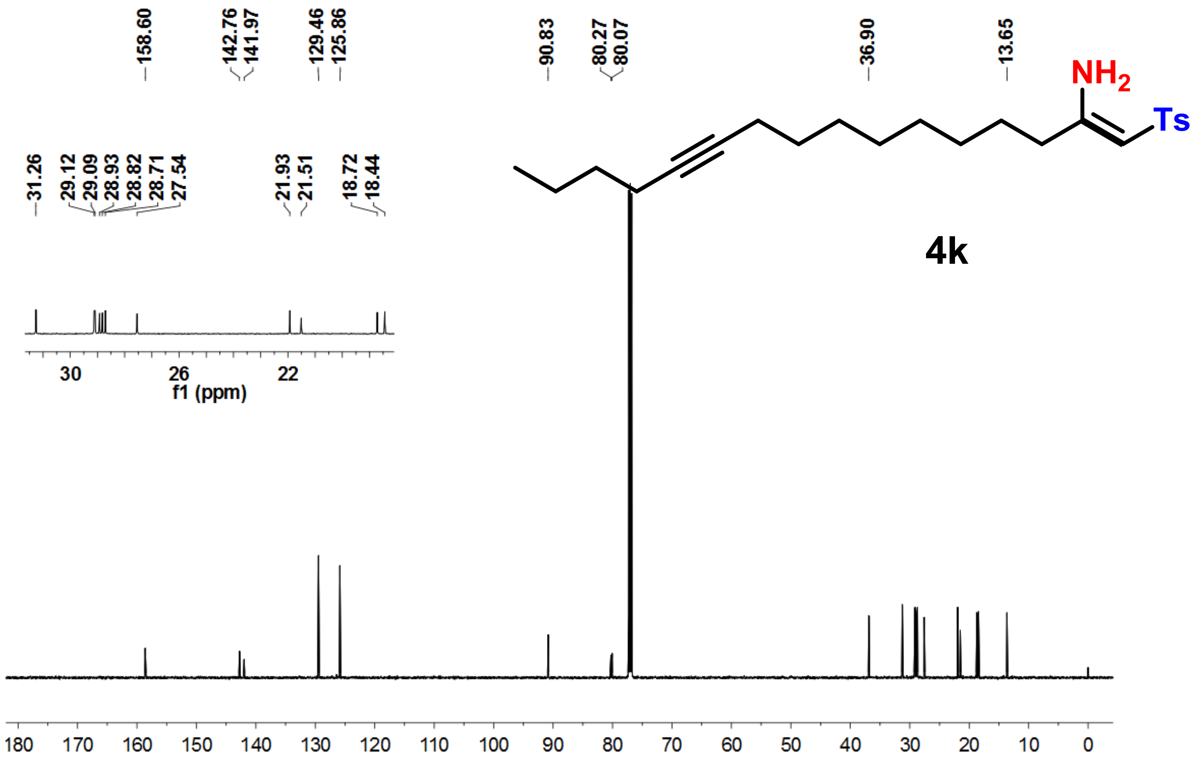
158.60
142.76
141.97
129.46
125.86
90.83
80.27
80.07
36.90
13.65
31.26
29.12
29.09
28.93
28.82
28.71
27.54
21.93
21.51
18.72
18.44
30
26
22
f1 (ppm)
NH2
Ts
4k
180 170 160 150 140 130 120 110 100 90 80 70 60 50 40 30 20 10 0

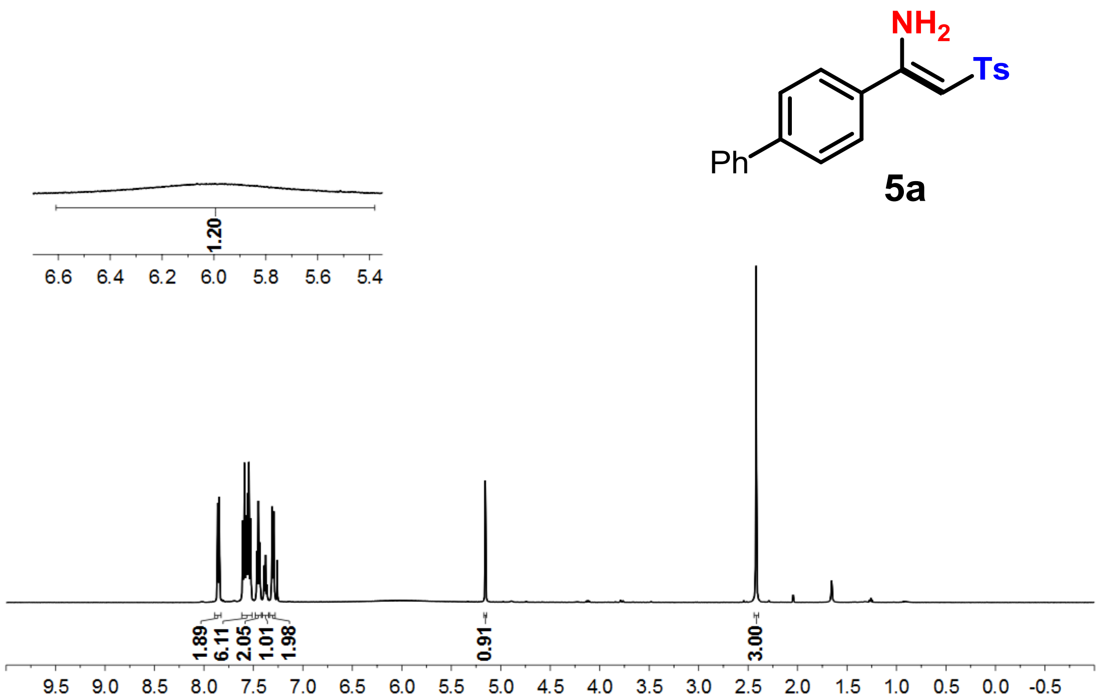
NH2
Ts
Ph
5a
1.20
6.6 6.4 6.2 6.0 5.8 5.6 5.4
1.89
6.11
2.05
1.01
1.98
0.91
3.00
9.5 9.0 8.5 8.0 7.5 7.0 6.5 6.0 5.5 5.0 4.5 4.0 3.5 3.0 2.5 2.0 1.5 1.0 0.5 0.0 -0.5

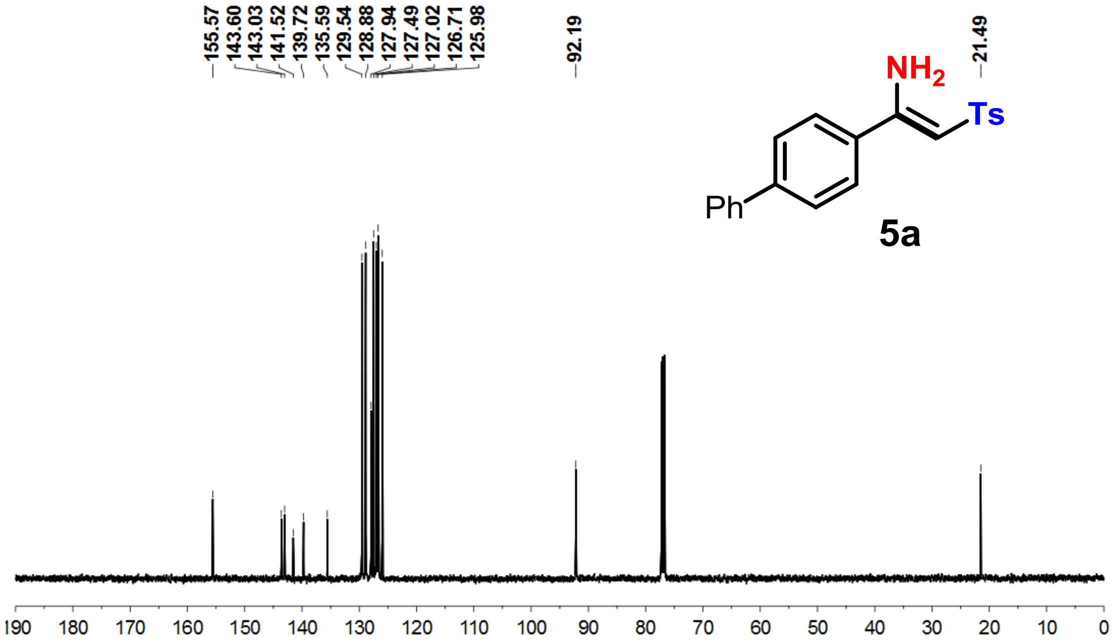
155.57
143.60
143.03
141.52
139.72
135.59
129.54
128.88
127.94
127.49
127.02
126.71
125.98
92.19
21.49
NH2
Ts
Ph
5a
190 180 170 160 150 140 130 120 110 100 90 80 70 60 50 40 30 20 10 0

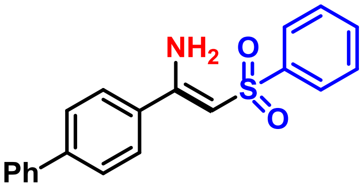
NH2
O
S
O
Ph

5b

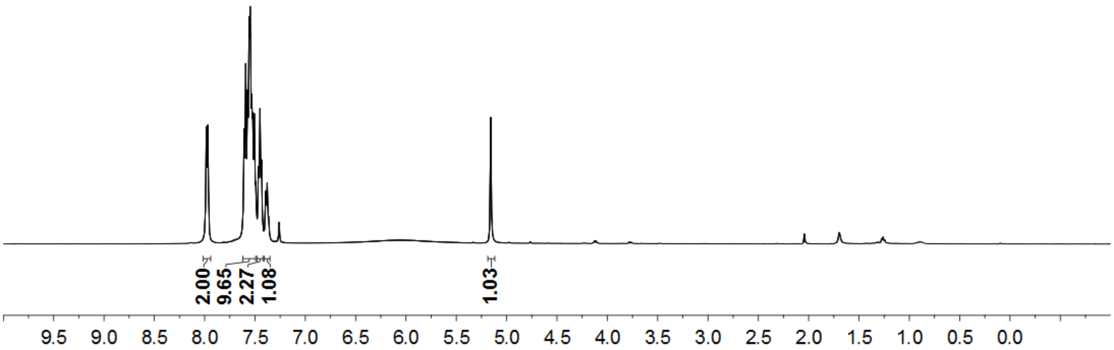
2.00
9.65
2.27
1.08
1.03
9.5 9.0 8.5 8.0 7.5 7.0 6.5 6.0 5.5 5.0 4.5 4.0 3.5 3.0 2.5 2.0 1.5 1.0 0.5 0.0

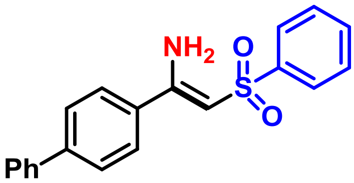
NH2
O
S
O
Ph

5b

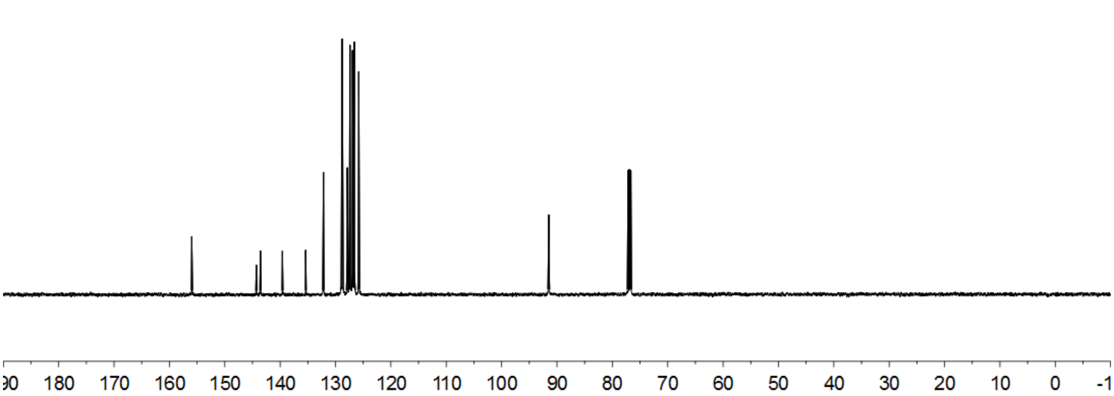
155.96
144.31
143.58
139.59
135.38
132.19
128.83
128.78
127.86
127.40
126.92
126.63
125.80
91.48
180 170 160 150 140 130 120 110 100 90 80 70 60 50 40 30 20 10 0

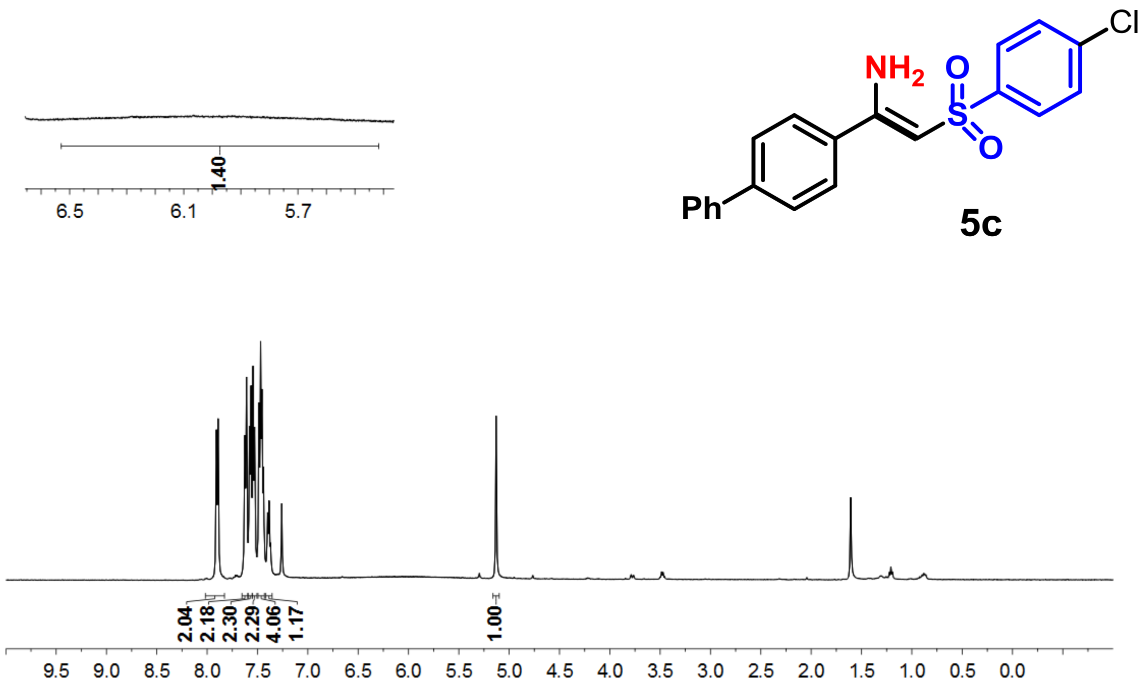
NH2
Cl
O
S
O
Ph
5c
1.40
6.5
6.1
5.7
2.04
2.18
2.30
2.29
4.06
1.17
1.00
9.5 9.0 8.5 8.0 7.5 7.0 6.5 6.0 5.5 5.0 4.5 4.0 3.5 3.0 2.5 2.0 1.5 1.0 0.5 0.0

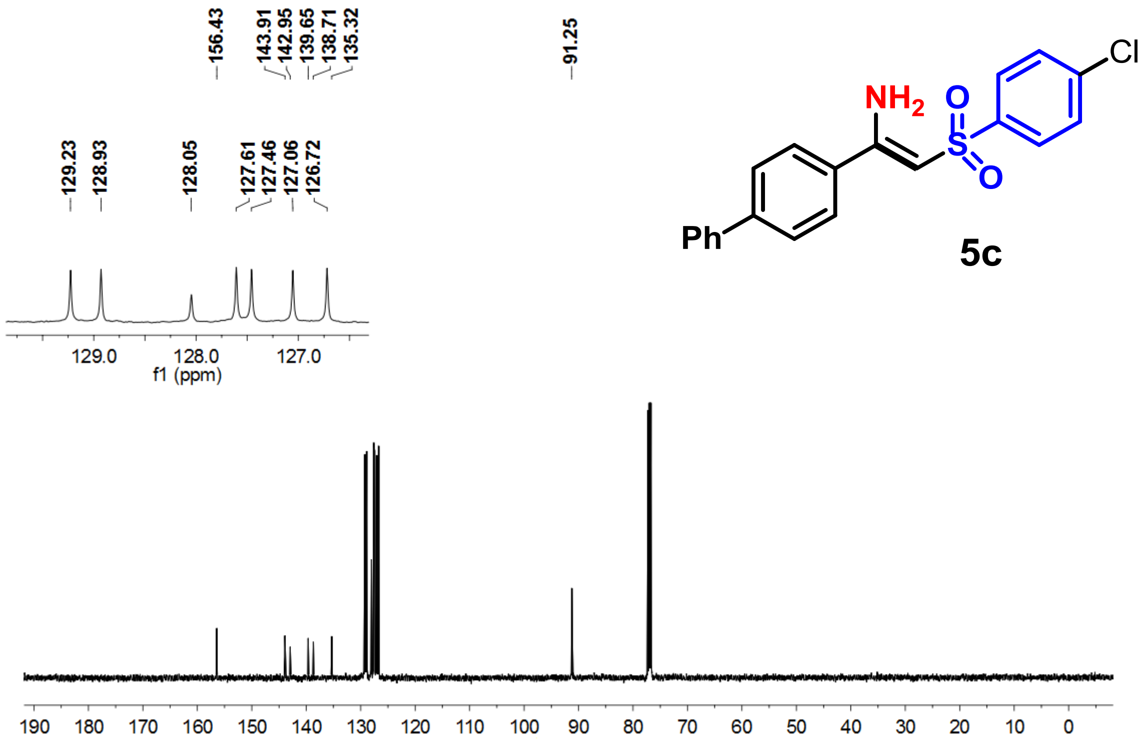
156.43
143.91
142.95
139.65
138.71
135.32
91.25
129.23
128.93
128.05
127.61
127.46
127.06
126.72
129.0
128.0
127.0
f1 (ppm)
NH2
Cl
O
S
O
Ph
5c
190 180 170 160 150 140 130 120 110 100 90 80 70 60 50 40 30 20 10 0

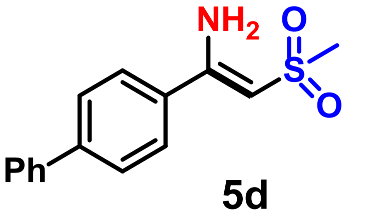
NH2
O
S
O
Ph

5d

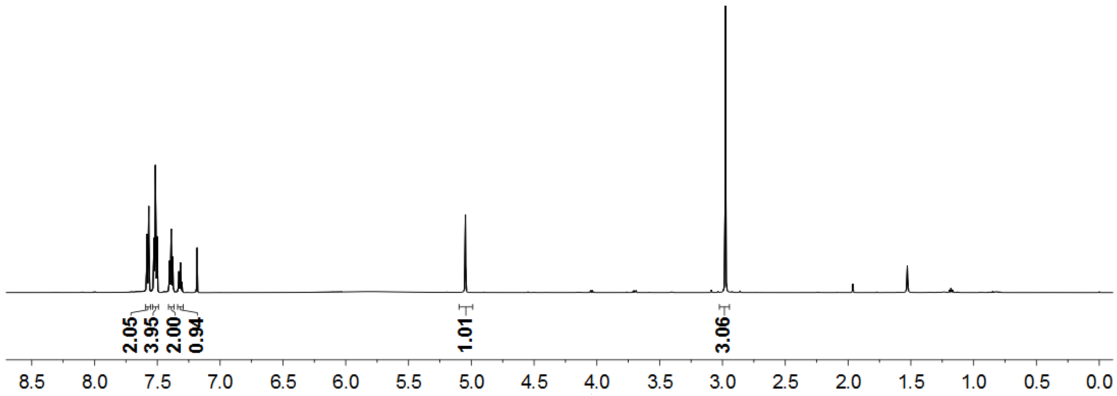
2.05
3.95
2.00
0.94
1.01
3.06
8.5 8.0 7.5 7.0 6.5 6.0 5.5 5.0 4.5 4.0 3.5 3.0 2.5 2.0 1.5 1.0 0.5 0.0

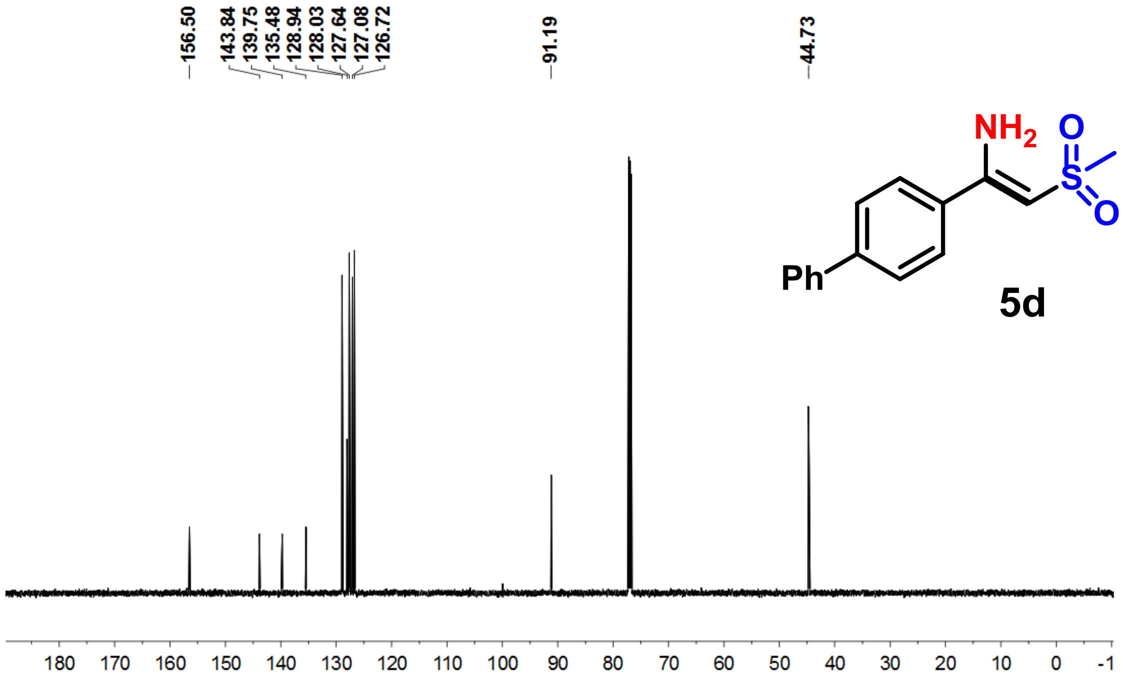
156.50
143.84
139.75
135.48
128.94
128.03
127.64
127.08
126.72
91.19
44.73
180 170 160 150 140 130 120 110 100 90 80 70 60 50 40 30 20 10 0 -1
NH2
O
S
O
Ph

5d

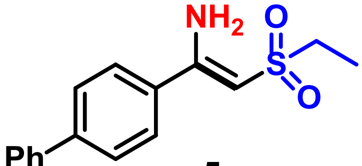

NH2
O
S
O
Ph

5e

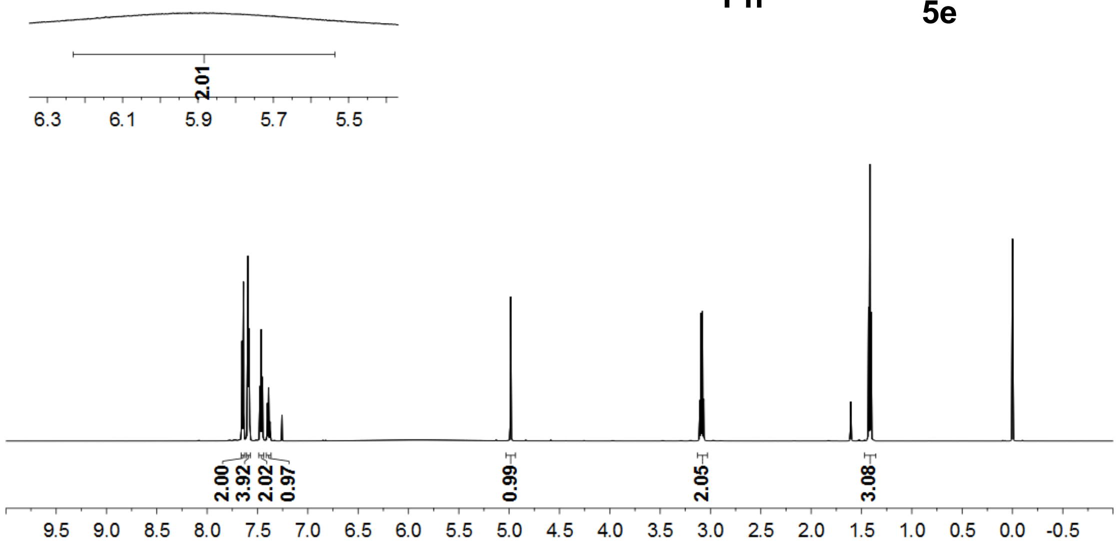

2.01
6.3
6.1
5.9
5.7
5.5
2.00
3.92
2.02
0.97
0.99
2.05
3.08
9.5
9.0
8.5
8.0
7.5
7.0
6.5
6.0
5.5
5.0
4.5
4.0
3.5
3.0
2.5
2.0
1.5
1.0
0.5
0.0
-0.5

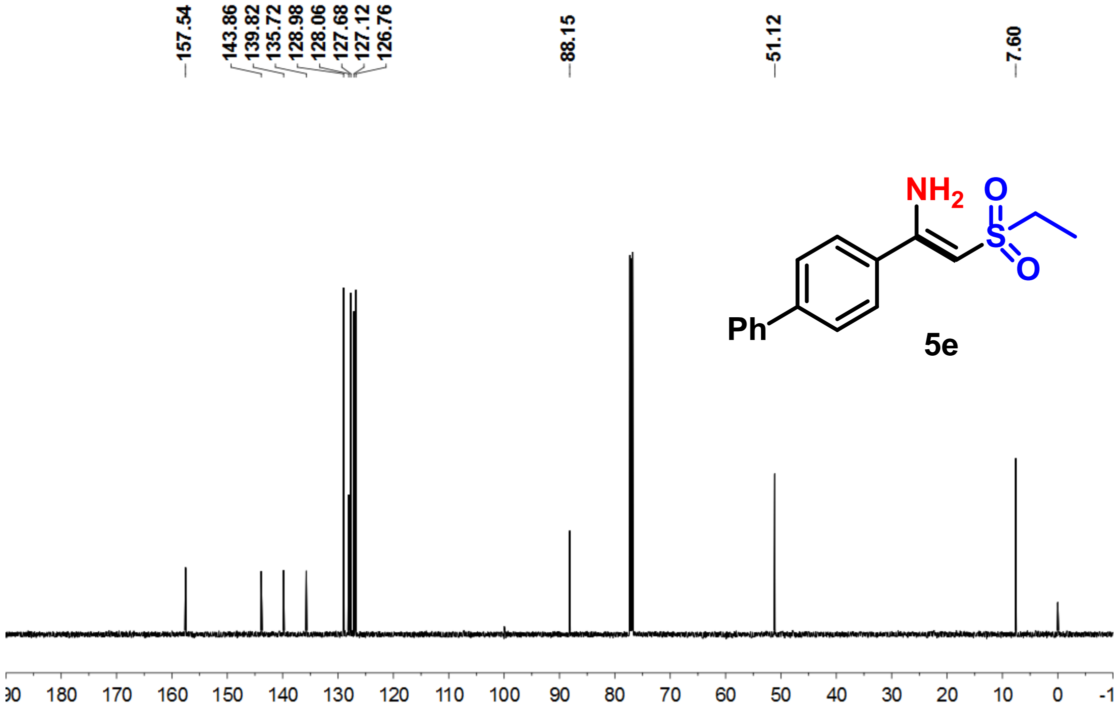

157.54
143.86
139.82
135.72
128.98
128.06
127.68
127.12
126.76
88.15
51.12
7.60
180
170
160
150
140
130
120
110
100
90
80
70
60
50
40
30
20
10
0
NH2
O
S
O
Ph

5e

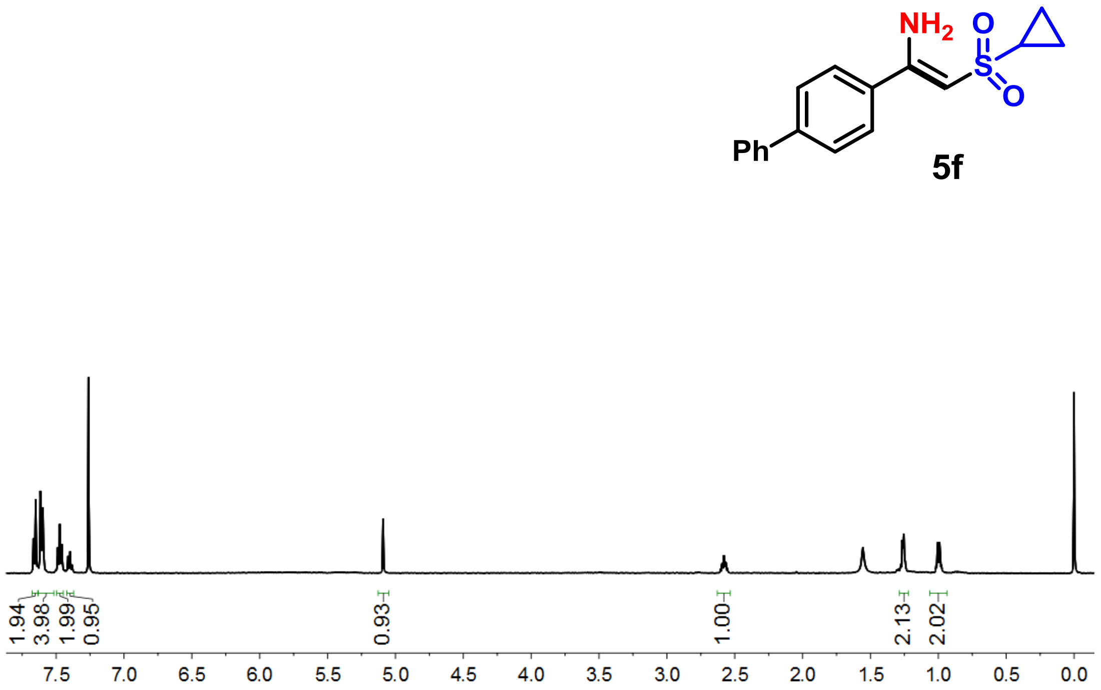
NH2
Ph
5f
1.94
3.98
1.99
0.95
0.93
1.00
2.13
2.02
7.5 7.0 6.5 6.0 5.5 5.0 4.5 4.0 3.5 3.0 2.5 2.0 1.5 1.0 0.5 0.0

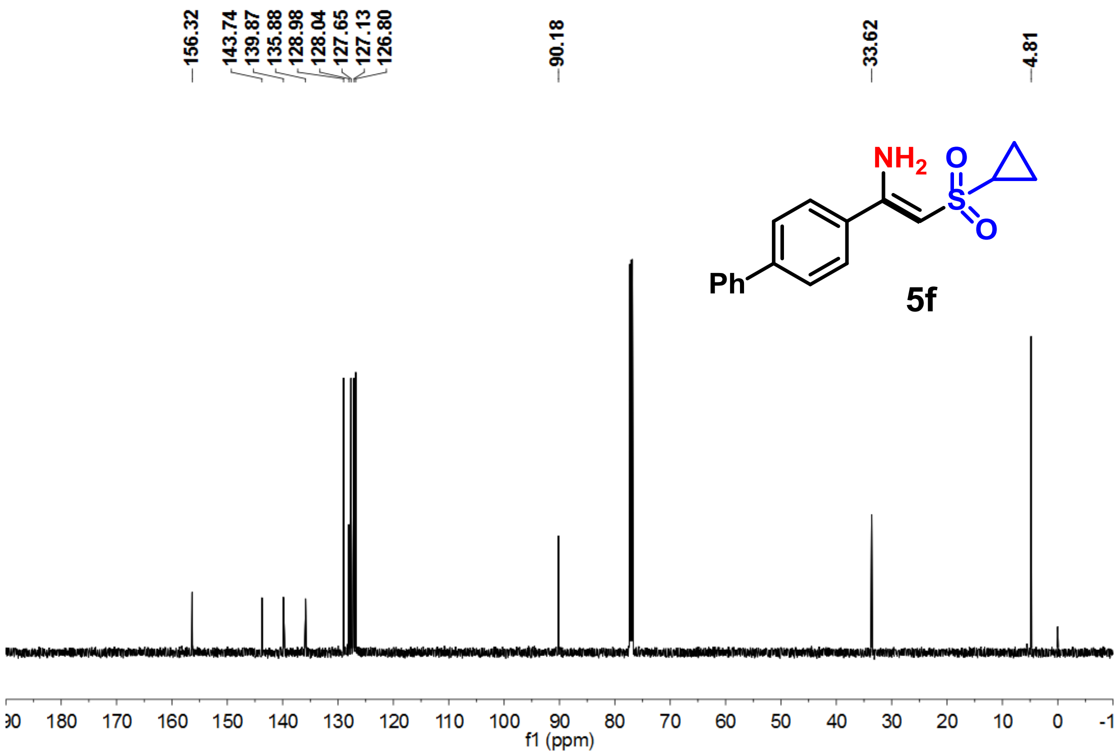
156.32
143.74
139.87
135.88
128.98
128.04
127.65
127.13
126.80
90.18
33.62
4.81
NH2
Ph
5f
180 170 160 150 140 130 120 110 100 90 80 70 60 50 40 30 20 10 0 -1
f1 (ppm)

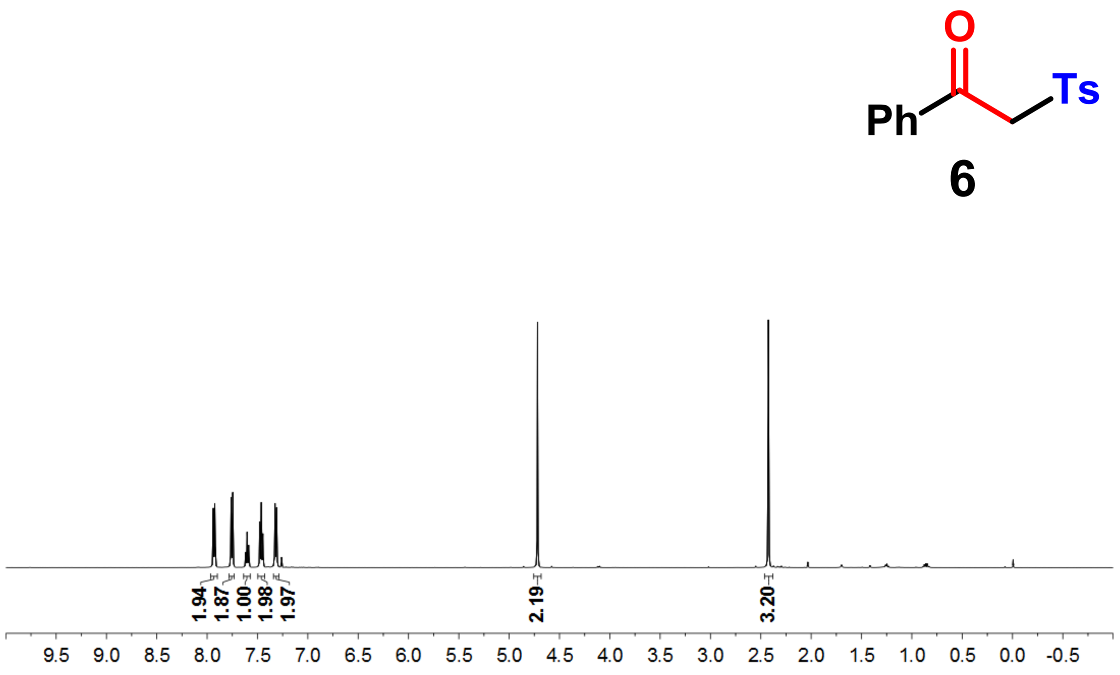
O
Ph
Ts
6
1.94
1.87
1.00
1.98
1.97
2.19
3.20
9.5 9.0 8.5 8.0 7.5 7.0 6.5 6.0 5.5 5.0 4.5 4.0 3.5 3.0 2.5 2.0 1.5 1.0 0.5 0.0 -0.5

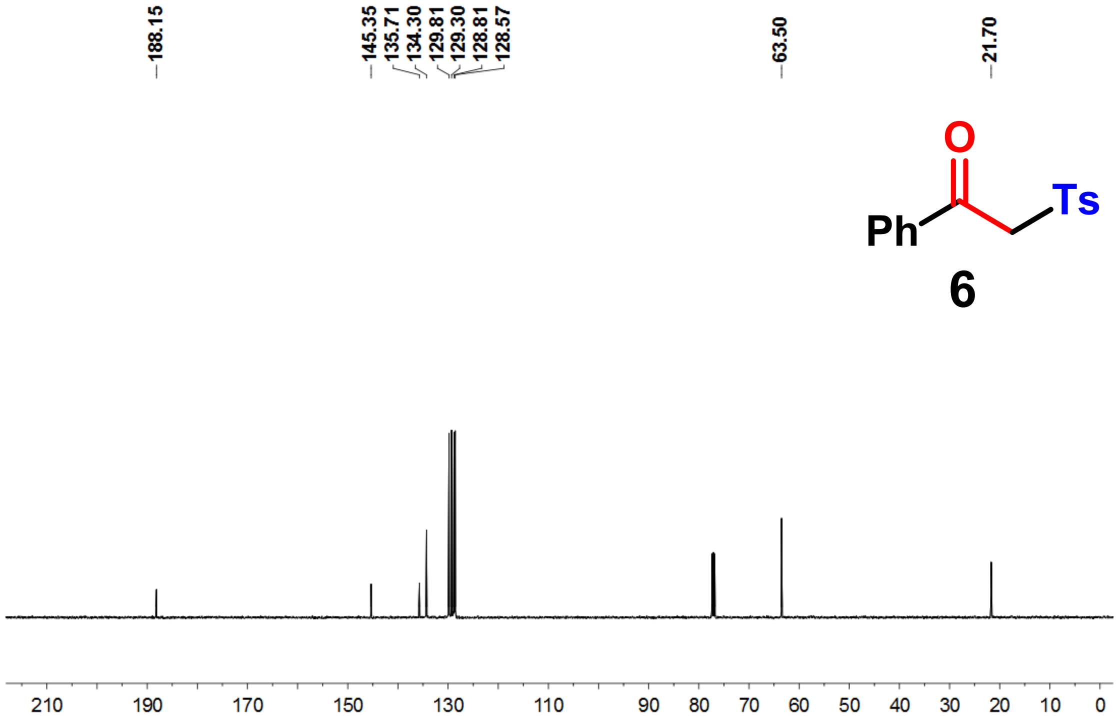
188.15
145.35
135.71
134.30
129.81
129.30
128.81
128.57
63.50
21.70
O
Ph
Ts
6
210 190 170 150 130 110 90 80 70 60 50 40 30 20 10 0

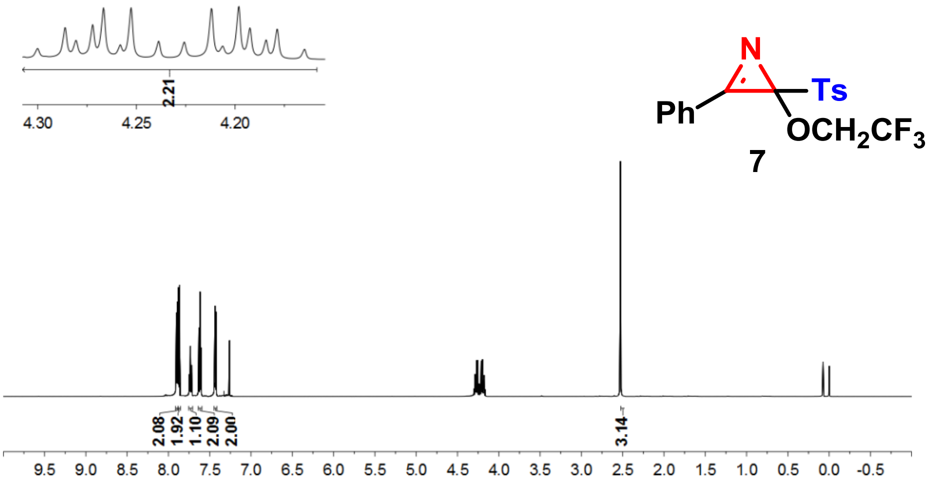

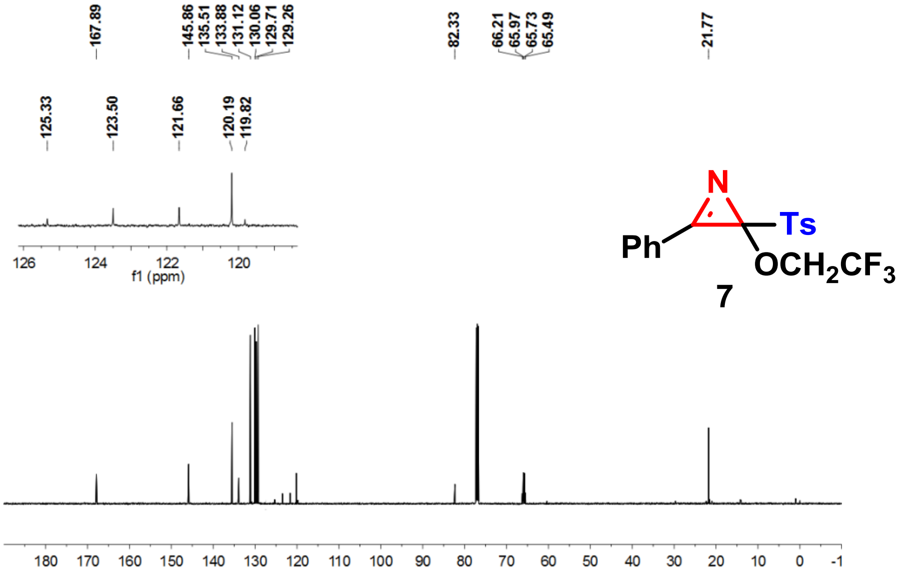

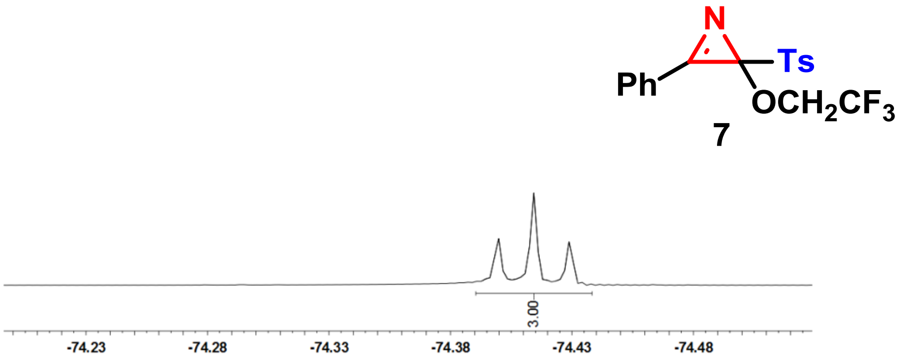

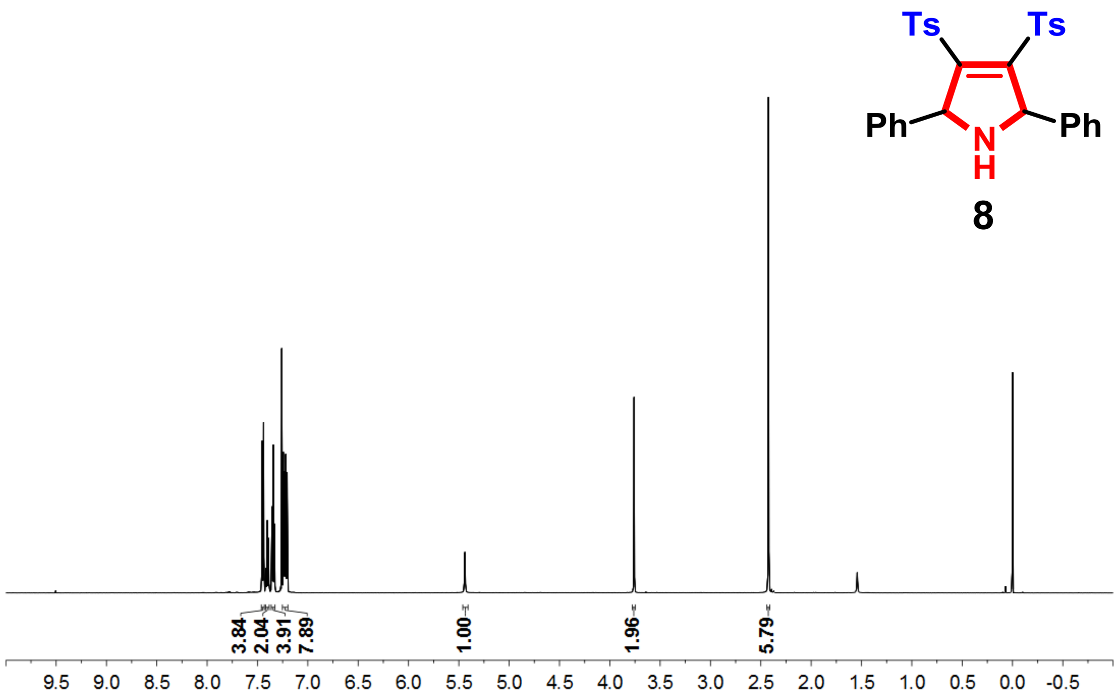

Ts
Ts
Ph
Ph
N
H
8
3.84
2.04
3.91
7.89
1.00
1.96
5.79
9.5 9.0 8.5 8.0 7.5 7.0 6.5 6.0 5.5 5.0 4.5 4.0 3.5 3.0 2.5 2.0 1.5 1.0 0.5 0.0 -0.5

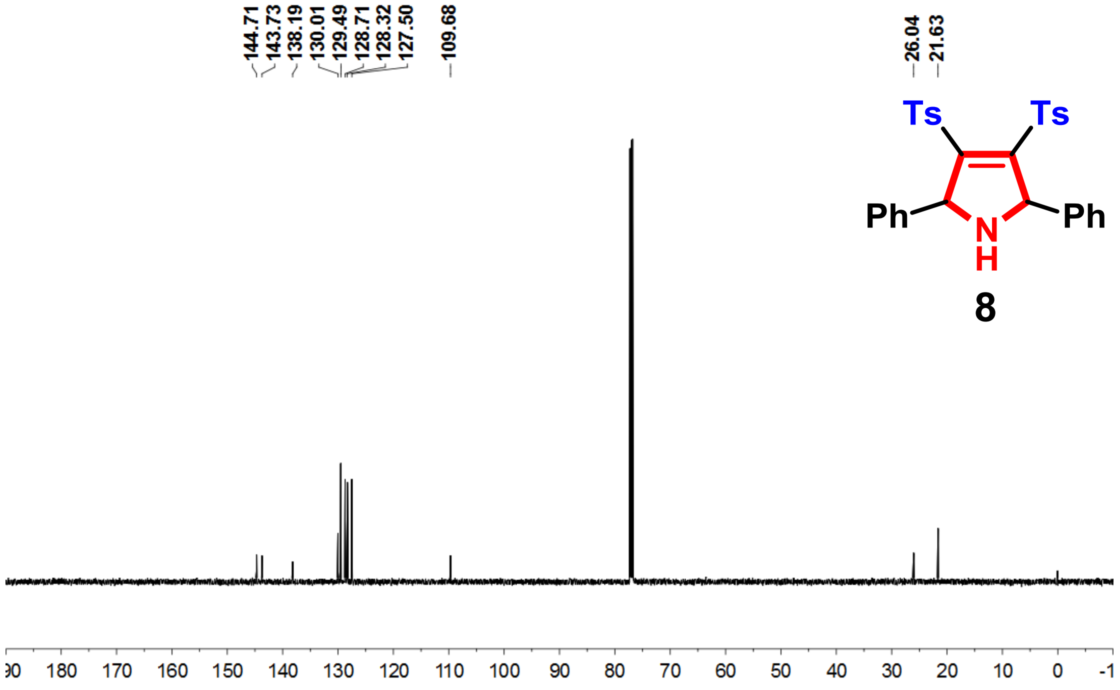

144.71
143.73
138.19
130.01
129.49
128.71
128.32
127.50
109.68
26.04
21.63
Ts
Ts
Ph
Ph
N
H
8
180 170 160 150 140 130 120 110 100 90 80 70 60 50 40 30 20 10 0

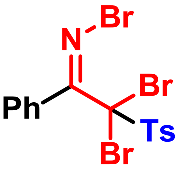
N
Br
Ph
Br
Ts
Br

9

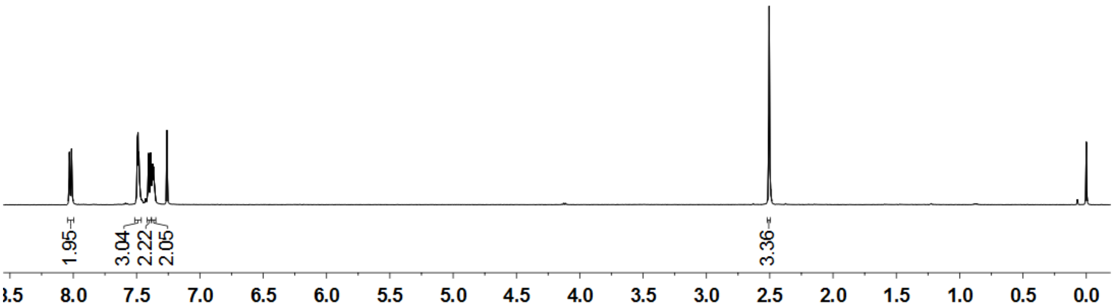
1.95
3.04
2.22
2.05
3.36
8.0 7.5 7.0 6.5 6.0 5.5 5.0 4.5 4.0 3.5 3.0 2.5 2.0 1.5 1.0 0.5 0.0

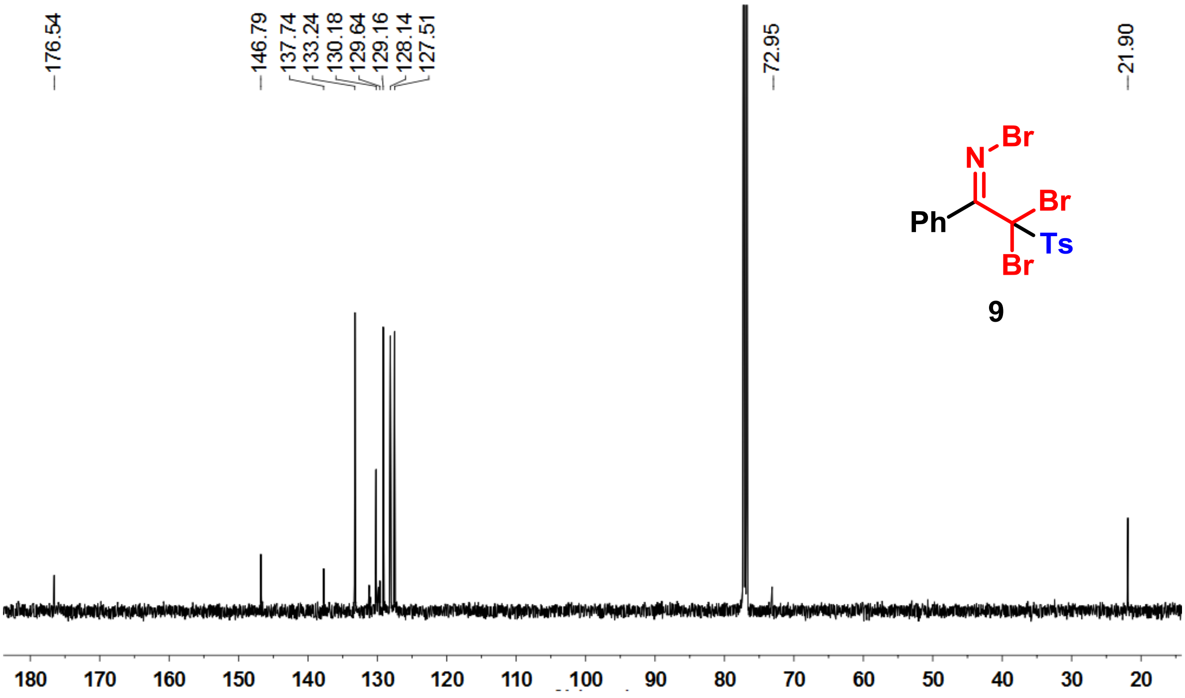
176.54
146.79
137.74
133.24
130.18
129.64
129.16
128.14
127.51
72.95
21.90
180 170 160 150 140 130 120 110 100 90 80 70 60 50 40 30 20
N
Br
Ph
Br
Ts
Br

9
